# Dual-target ligand discovery for Alzheimer’s disease: triphenylphosphoranylidene derivatives as inhibitors of acetylcholinesterase and β-amyloid aggregation

**DOI:** 10.1080/14756366.2023.2166040

**Published:** 2023-01-24

**Authors:** Marwa El-Hussieny, Mansoura A. Abd-El-Maksoud, Fouad M. Soliman, Marwa A. Fouad, Mohamed K. El-Ashrey

**Affiliations:** a Organometallic and Organometalloid Chemistry Department, National Research Centre, Giza, Egypt; b Pharmaceutical Chemistry Department, Faculty of Pharmacy, Cairo University, Cairo, Egypt; c Pharmaceutical Chemistry Department, School of Pharmacy, NewGiza University, Cairo, Egypt

**Keywords:** Triphenylphosphoranylidene, silane, acetylcholinesterase, β-amyloid, Alzheimer’s disease

## Abstract

Alzheimer disease (AD) is one of the major neurodegenerative diseases that could not be prevented or completely cured and may lead to death. Here, we target AChE and β-amyloid proteins. Synthesising new triphenylphosphporanylidene derivatives based on the surveyed literature and testing their biological activity revealed promising results especially for the acetyl triphenylphosphoranylidene derivative **8c**, which showed good inhibitor activity against AChE enzyme with IC_50_ in the nanomolar range (97.04 nM); on the other hand, it showed poor selectivity for AChE versus butyrylcholinesterase but with some futural structural modification, this selectivity can be improved. **8c** showed MMP-2 IC_50_ of 724.19 nM and Aβ_1-42_ aggregation IC_50_ of 302.36 nM. A kinetic study demonstrated that compound **8c** uncompetitively inhibited AChE. Moreover, derivative **8c** showed low cytotoxicity, good *in vivo* behavioural studies including Y-maze and passive avoidance tests with activity similar to that of donepezil. Finally, *in silico* studies for **8c** predict its good penetration into BBB and good binding affinity in the AChE binding site.

## GRAPHICAL ABSTRACT

**Figure:**
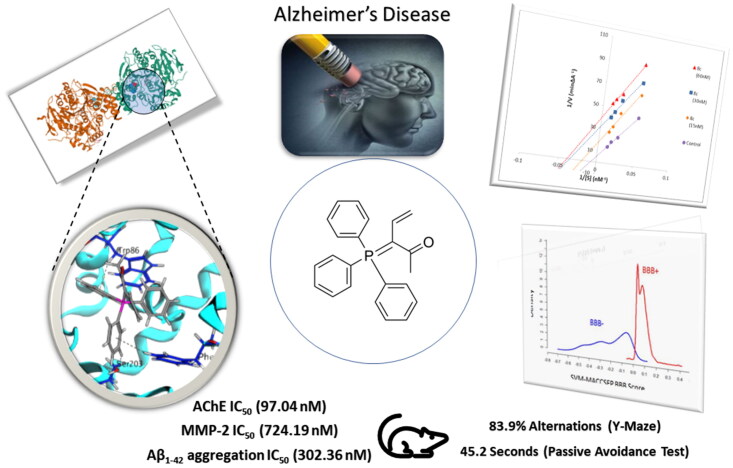

## Introduction

Alzheimer disease (AD) is one of the neurodegenerative disorders that affects not only the patient but also the family and the caregiver^1^. There are numerous theories that have been put forth to explain the causes, including the aggravation of ageing, the degeneration of cholinergic and cortico-cortical pathways, environmental factors like aluminium exposure, head injuries, malnutrition, mutations of the amyloid precursor protein (APP) and PSEN genes, allelic variation in apolipoprotein E, mitochondrial dysfunction, immune system dysfunction, and infectious agents^2^. AD is categorised as the third cause of death in the world after heart and cancer diseases and the affected people are expected to be more than ten million people by the next 30 years^3^.

**Figure 1.**
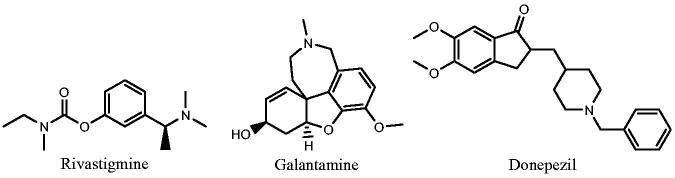
Examples of approved AChE inhibitors used for AD.

Acetylcholinesterase (AChE) enzyme is the primary cholinesterase in the human body, it catalyses the hydrolysis of the neurotransmitters, acetylcholine (ACh) and other choline esters^4^. Each molecule of AChE hydrolyses about 25 000 of ACh per second revealing its very high catalytic activity^5^. Drug or toxin that inhibits AChE leads to persistent high level of ACh within the synapses with increasing cholinergic activity. Irreversible AChE inhibitors are used as insecticides while reversible inhibitors are useful for several diseases such as Alzheimer’s disease^6^. Many chemical derivatives showed good inhibitory activity against AChE^7^^,^^8^, rivastigmine, galantamine, and donepezil are examples of approved AChE inhibitor drugs^9–11^ (Figure 1).

Butyrylcholinesterase (BuChE) is a non-specific cholinesterase enzyme that hydrolyses many choline-based esters and found mainly in blood plasma^12^. Inhibition of BuChE in the periphery causes severe side effects such as the known AChE inhibitor Tacrine^13^. Also, this enzyme shows a compensatory effect in response to a greatly decreased AChE activity in the central nervous system (CNS) during AD progression^14^. So, it is valuable to make a selective AChE inhibitor ligand with minimum effect on the peripheral BuChE^15^.

On the other hand, amyloids are aggregates of proteins, where β-amyloids (Aβ) are the main components of amyloid plaques found in patients with AD^16^^,^^17^. Aβ are formed by the action of β- and γ-secretase enzymes on the precursor protein^18^. Aβ molecules can aggregate to form some oligomers, which are toxic to nerve cells^19^. The other protein implicated in AD is the tau (*τ*) protein, which also forms misfolded oligomers^20^. Aβ_1–40_ and Aβ_1–42_ are two isoforms of Aβ, Aβ_1–42_ is less soluble and so more susceptible to aggregation^21^. It was also found that amyloid monomers interact with the peripheral active site (PAS) of AChE promoting the conformational changes that occur at amyloid proteins leading to an increase in their aggregation^22^. So, inhibiting the formation of Aβ plaques is a powerful way to counteract the central degeneration that occurs in AD.

Moreover, matrix metalloproteinases (MMPs); which are present normally in low level and play an important role in tissue remodelling associated with various biological processes^23^ are thought to degrade APP, which causes aggregation of Aβ. Additionally, it was noted that post-mortem brain tissue from AD patients had an enhanced expression of MMPs^24^.

Based on the mentioned causes of AD, medicinal chemists are in a continuous journey to find a multitarget ligand that is able to; not only relieve the symptoms; but also slow down the progression of the disease^25^. In the last decade, our research group has focussed on the synthesis of novel heterocyclic and homocyclic compounds, which have phosphorus moieties; these derivatives have industrial and biological importance^26–31^.

**Figure 2.**
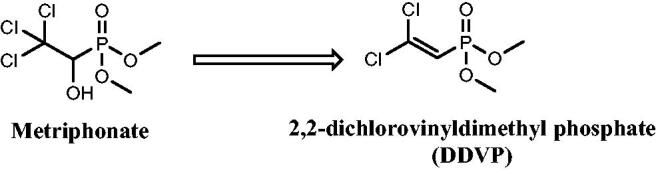
Non-enzymatic activation of metriphonate.

There are numerous uses for organophosphorus chemicals in medicine for the treatment of AD, phosphonic acid derivatives were found to be extremely effective with an IC_50_ range of 0.01–0.2 µM^32^. Along with the nonenzymatic conversion of Metriphonate, a long-acting irreversible organophosphate AChE inhibitor, to the active ingredient Dichlorvos; (Figure 2), clinical trials were started to treat AD patients because of its capacity to improve cholinergic neurotransmission in the CNS. But, in a 6-months study on AD treatment, respiratory paralysis and problems with neuromuscular transmission were seen^33^^,^^34^.

**Figure 3.**
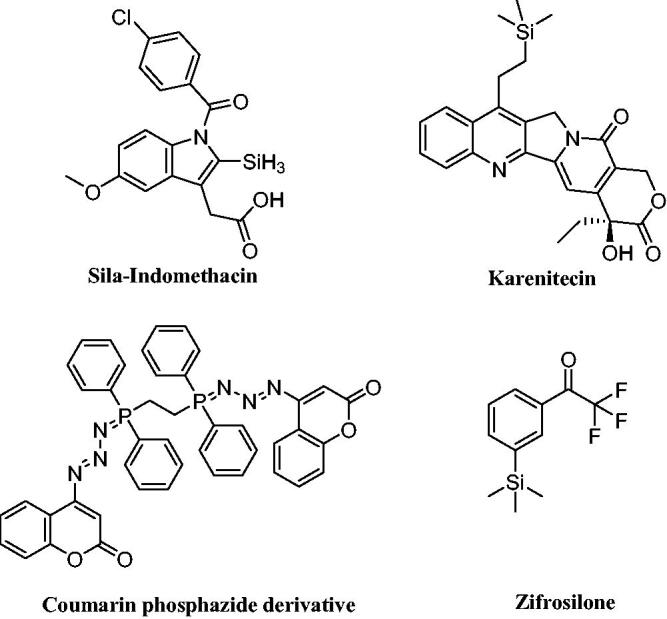
Reported silicone-containing compounds and coumarin phosphazide derivative.

Because silicon-containing molecules, such as those with methylsilyl, silanol, and silandiol groups, differ from those with carbon–carbon bases in terms of their chemical properties, organosilicon compounds have a variety of interesting applications, particularly in medicine as anti-inflammatory, antibacterial, and anticancer agents^35^. Through the silyl group, silicon can be incorporated into organic molecules to offer them special features like enhancing lipophilicity, which promotes cell and tissue penetration and consequently increases potency and selectivity of organosilicon rather than carbon-based molecule^36^. In addition, several medications have the silyl moiety, including the non-steroidal anti-inflammatory medicine, Sila-indomethacin^37^ and the anticancer organosilicon drug, BNP-1350 (Karenitecin)^38^. A silicon-containing compound called zifrosilone underwent clinical testing to treat Alzheimer’s disease (Figure 3)^39^.

Furthermore, we previously reported the discovery of a new coumarin phosphazide derivative as a selective and potent AChE and β-amyloid aggregation inhibitor^40^. In conjunction with this work and based on the previous facts about the importance of phosphorus compounds as inhibitor for the Alzheimer’s disease, we have synthesised some novel triphenylphosphoranylidenesilol-ylidene aniline, triphenylphosphoranylidene-silolone, triphenylphosphoranylidenebutanoates, and cyclobutenes, as AChE inhibitors. Besides, the most potent compound was assessed for its AChE/BuChE selectivity, self-induced Aβ aggregation and MMP-2 inhibition ability. In addition, the most potent compound **(8c)** was studied for AChE inhibition kinetics. Moreover, it was studied for neuroblastoma toxicity and its ability to ameliorate scopolamine-induced cognitive impairment in rats. Then, it was subjected to a molecular docking simulation to establish its binding affinity and mode of action in the enzyme’s binding site. Finally, drug-likeness prediction was utilised to analyse the pharmacokinetic properties.

## Results and discussion

### Chemistry

**Scheme 1.**
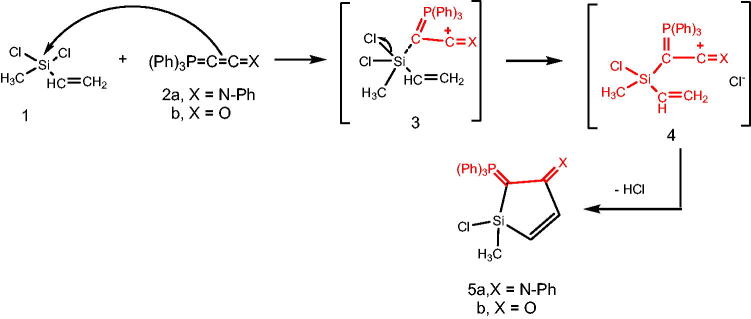
Synthesis of compounds 5(a & b) in THF.

Reacting (N-phenyliminovinylidene)triphenylphosphorane (**2a**) with dichloromethylvinylsilane (**1**) in tetrahydrofuran (*THF*), afforded *N*-(1-chloro-1-methyl-2-((triphenylphosphoranylidene)methylene)-1*H*-silol-3(2*H*)-ylidene)aniline (**5a**). Chlorosilanes are generally recognised to be the most popular substrates for displacement reactions that produce high yields of substitution products, even with weak nucleophilic reagents under mild conditions^41^. So, it is believed that addition of the nucleophilic phosphacumulene (**2a**) to the vinyl silane (**1**) yields the intermediates (**3)** then (**4)**. Cyclisation of the salt (**4)** afforded the phosphoranylidene silol ylidene aniline (**5a)**. The structure of (**5a**) was substantiated by its spectral data. The ^31^P-NMR spectrum recorded a chemical shift for (**5a**) at δ 17.30 ppm. In the mass spectrum, the M^+^ appeared at 484. Reaction of (2-oxovinylidene)triphenylphosphorane (**2b**) with vinylsilane (**1**) afforded the corresponding 1-chloro-1-methyl-2-(triphenylphosphoranylidene)-1H-silol-3(2H)-one (**5b**). Its ^31^P NMR spectrum showed a peak at δ 19.40 ppm and in its mass spectrum, the M^+^ appeared at 406 (Scheme 1).

**Scheme 2.**
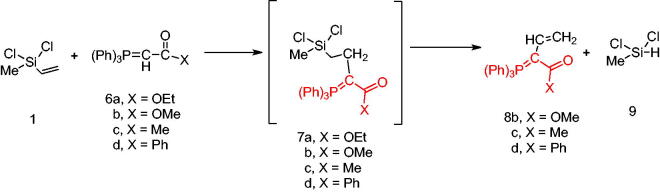
Synthesis of compounds 8 (a–c) in THF.

Next, the behaviour of the stabilised phosphonium ylides (**6a–d**) towards the vinylsilane (**1**) was also investigated in order to identify the place of the attack. It was found that, the ethoxycarbonyl-(**6a**), methoxycarbonyl-(**6b**), acetyl-(**6c**) or benzoyl-methylene triphenylphosphoranes (**6d**) reacted with vinylsilane (**1**) in *THF* yielding the phosphoranylidene butanoate derivatives (**7a–d**), which were isolated when the ethoxycarbonyl methylene triphenylphosphorane (**6a**) was used only. But in case of (**6b–d**), expulsion of the good leaving group dichloro(methyl)silane ^42^ gave the corresponding triphenylphosphoranylidene derivatives (**8b–d**) by good yield. The structure of compound (**7a**) was established based on spectroscopic data. Also, the structure of compounds (**8b–d**) was elucidated on the basis and assignments of elemental and spectroscopic analysis such as in compound (**8d),** the ^31^P NMR spectrum shift was recorded at δ 21.66 ppm (Scheme 2).

**Scheme 3.**
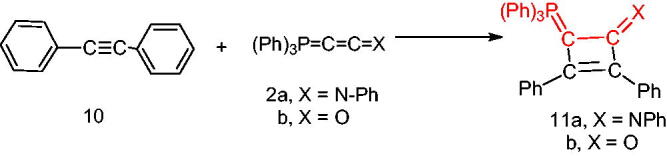
Synthesis of compounds 11 (a & b) in the presence of PdCl_2_.

Furthermore, we examined the reaction of 1,2-diphenylethyene (**10**) with the phosphacumulenes (**2a,b**). The reaction was carried out in boiling toluene in the presence of PdCl_2_ affording the phosphoranylidene cyclobutene structures (**11a**, **11b**), which were substantiated by their ^31^P NMR and MS spectral data. The ^31^P spectrum of (**11a**) revealed a signal at δ 22.25 ppm and in the MS, the *m/z* (%) appeared at 556 (M^+^). The ^31^P NMR spectrum of (**11b**) showed a signal at δ 33.94 ppm and the mass spectrum showed a molecular ion peak at *m/z* 481 (M^+^; Scheme 3).

**Scheme 4.**
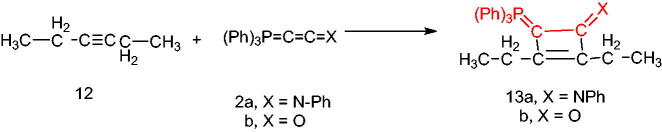
Synthesis of compounds 13 (a & b) in boiling toluene in the presence of PdCl_2_.

On the other hand, by analogous procedure, N-[2,3-diethyl(triphenylphosphoranylidene)cyclobut-2-en-1-ylidene)aniline (**13a)** was obtained from the reaction of hex-3-yne (**12**) with the (N-phenyliminovinylidene)triphenylphosphorane (**2a**), in boiling toluene in the presence of PdCl_2_. The ^1^HNMR spectrum of (**13a**) showed signals at δ 1.75 (t, 3H, CH_3_), 2.00 (q, 2H, CH_2_) and its mass spectrum indicated the presence of the molecular ion peak at *m/z* 458 (M^+^). Furthermore, the reaction of the hexyne (**12**) with the phosphacumulene (**2b**) was performed to give the phosphoranylidene cyclobutenone (**13b**). A signal at δ 28.31 was observed in its ^31^P NMR spectrum (Scheme 4).

**Scheme 5.**
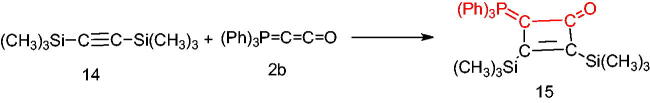
Synthesis of compound 15 in refluxing toluene.

Finally, treatment of bis(trimethylsilyl)acetylene (**14**) with the phosphacumulene (**2b**) in refluxing toluene, afforded the corresponding 2,3-bis(trimethylsilyl)-4-(triphenylphosphoranylidene)cyclobut-2-enone (**15**) (Scheme 5).

### In vitro *biological evaluation*

#### In vitro *AChE, BuChE, and MMP-2 inhibition assays*

**Figure 4.**
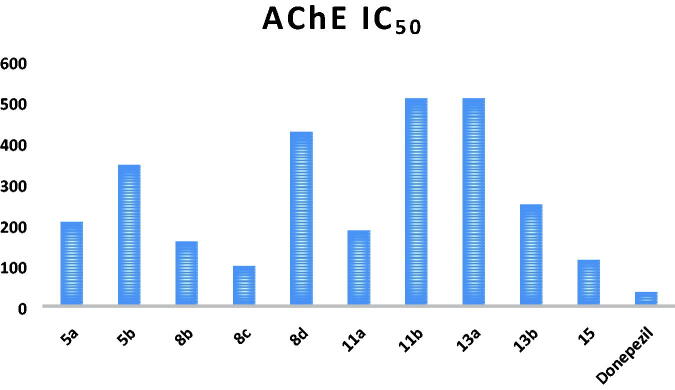
AChE IC_50_ of the tested compounds compared to that of donepezil.

First, AChE inhibitory activity was tested for all the synthesised final compounds using the improved Ellman spectrophotometric method^43^ and donepezil was used as the reference compound. The tested compounds showed IC_50_ values ranging from 97.04 to 508.03 nM compared to that of donepezil (34.42 nM; Table 1 and Figure 4). The triphenylphosphoranylidene silyl derivatives (**5a** and **5b**) showed relatively high IC_50_ values (206.25 and 345.04 nM, respectively), and it can be seen that replacing the aniline moiety in **5a** with carbonyl substitution as in compound **5b** causes a notable decrease in the AChE inhibitory activity. On the other hand, the other triphenylphosphoranylidene derivatives (**8c and 8b**) have relatively low IC_50_ (97.04 and 157.78 nM, respectively) especially the acetyl derivative (**8c**). On the contrary, compound **8d** shows high IC_50_ (426.35 nM) indicating that benzoyl substitution dramatically decreases the inhibitory activity. Cyclobutene derivatives **11a** and **11b** showed extremely variable results as the aniline derivative (**11a**) showed a moderate inhibitory activity with IC_50_ of 184.73 nM while the oxo derivative (**11b**) showed the highest IC_50_ value among all the tested compounds (508.03 nM). Other cyclobutene derivatives with ethyl substitution (**13a** and **13b**) showed also variable results but on the contrary the oxo derivative (**13b**) revealed moderate activity (IC_50_ of 248.82 nM) while the aniline derivative (**13b**) showed high IC_50_ value of 507.05 nM. Finally, bis(trimethylsilyl)cyclobutenone derivative (**15**) has a good inhibitory activity with IC_50_ of 113.05 nM.

**Table 1.** IC_50_ values of the tested compounds and the reference drug donepezil against AChE enzyme.

Compound	IC_50_ (nM)^a^ ± SD	Compound	IC_50_ (nM)^a^ ± SD
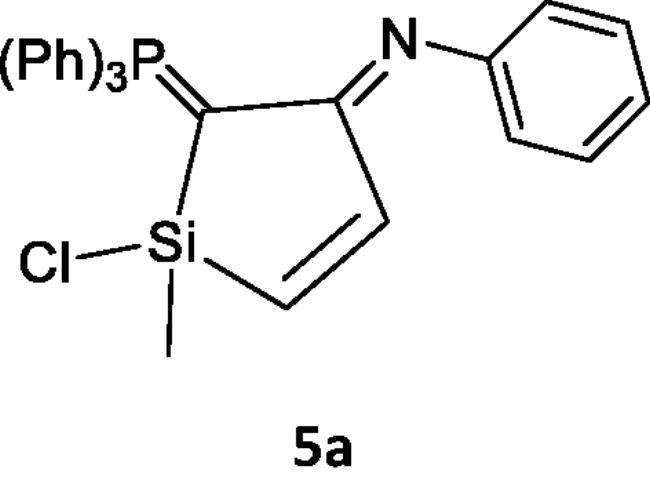	206.25 ± 7.33	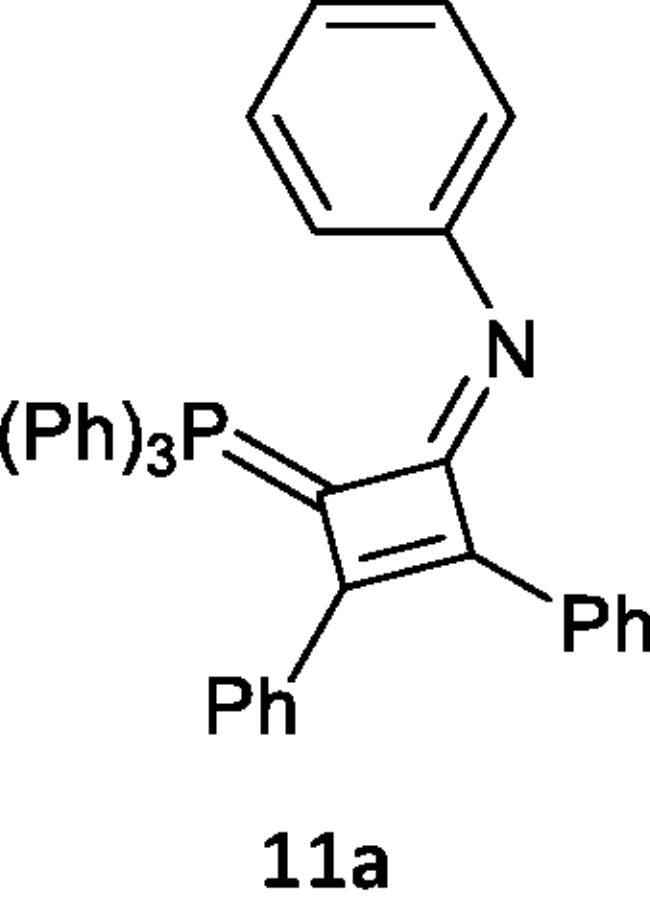	184.73 ± 9.22
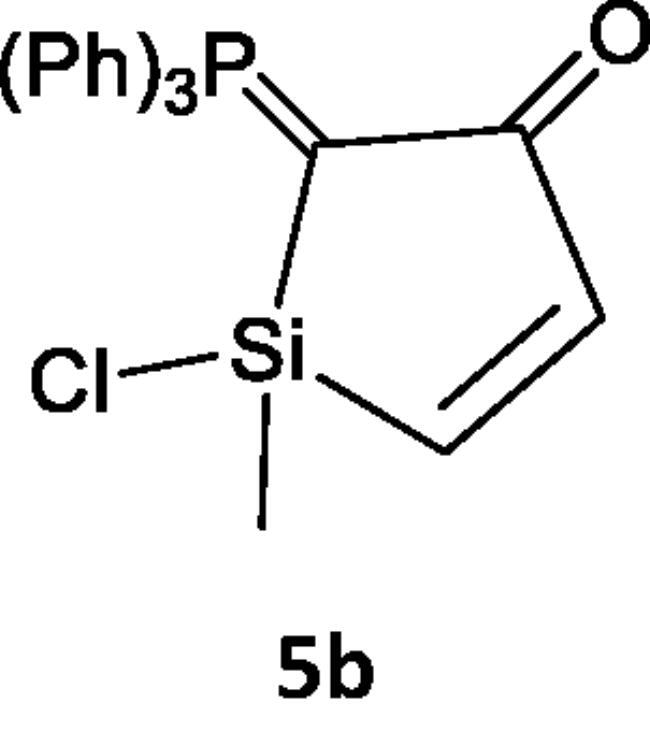	345.04 ± 12.3	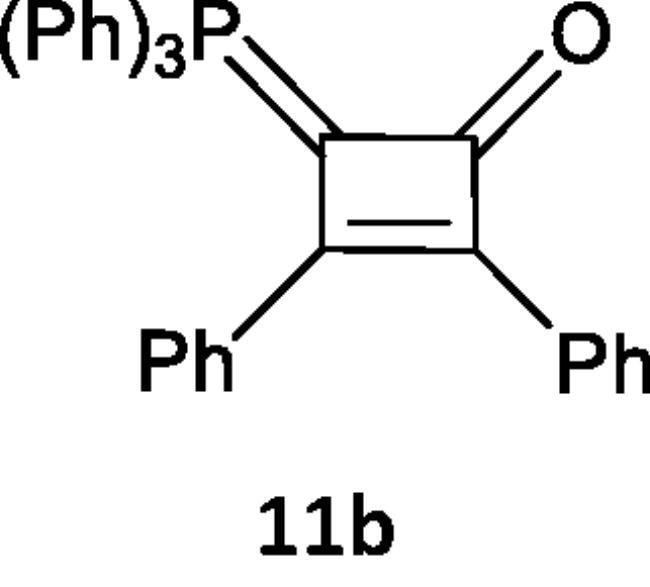	508.03 ± 16.5
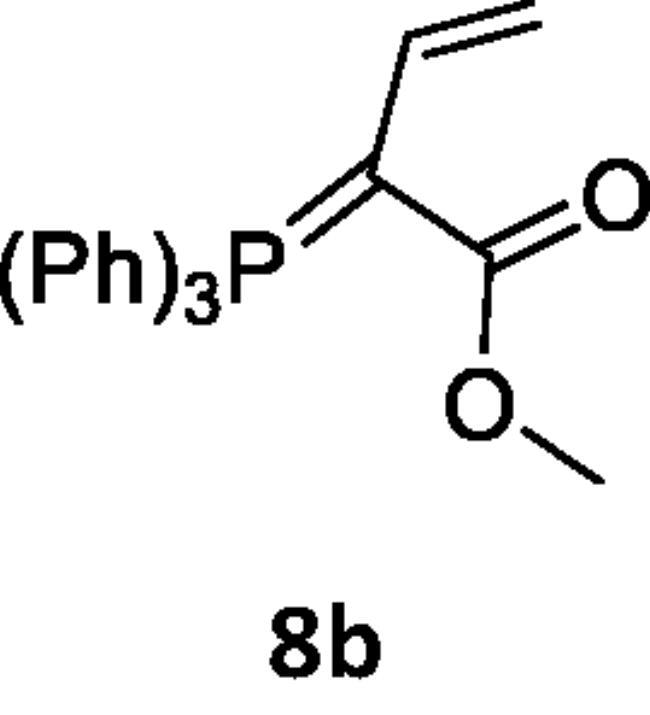	157.78 ± 4.28	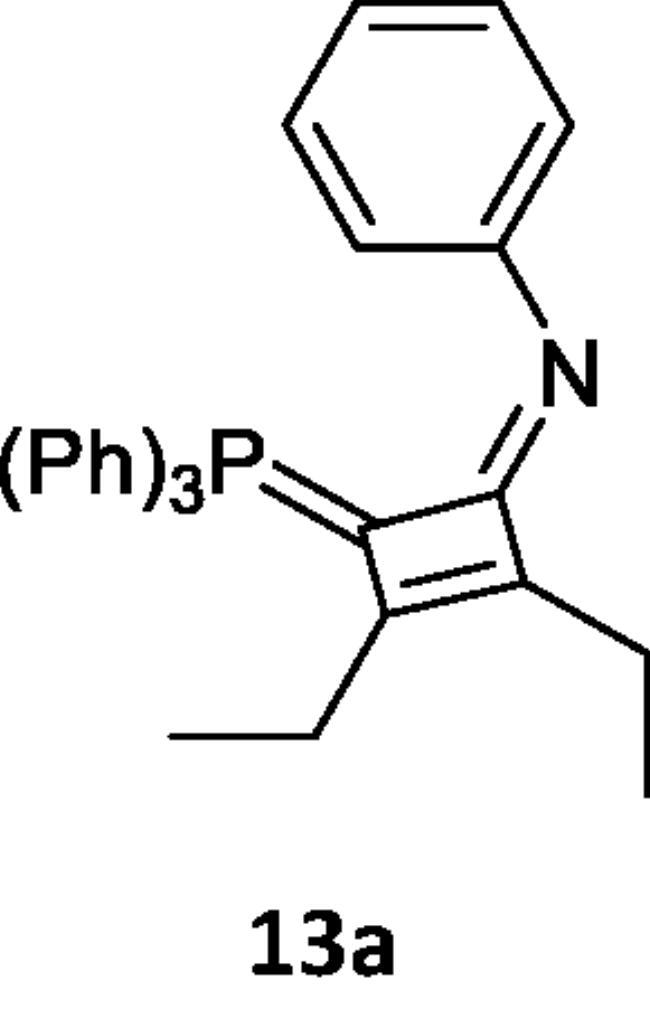	507.05 ± 22.1
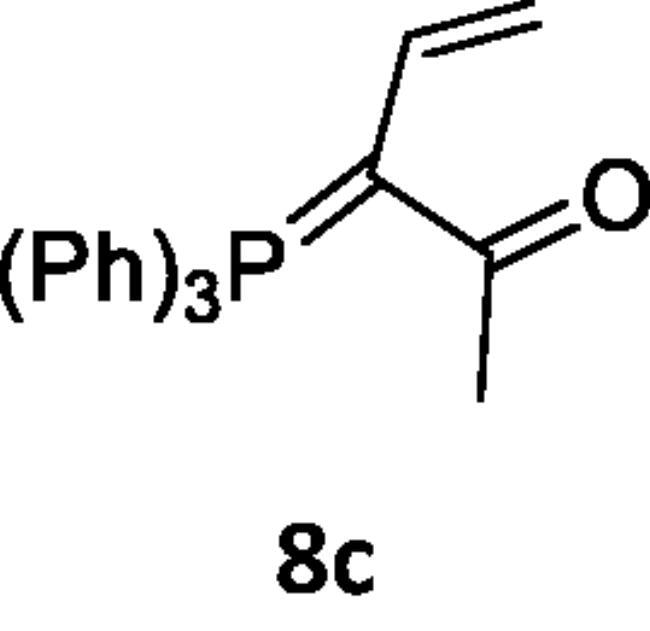	97.04 ± 2.93	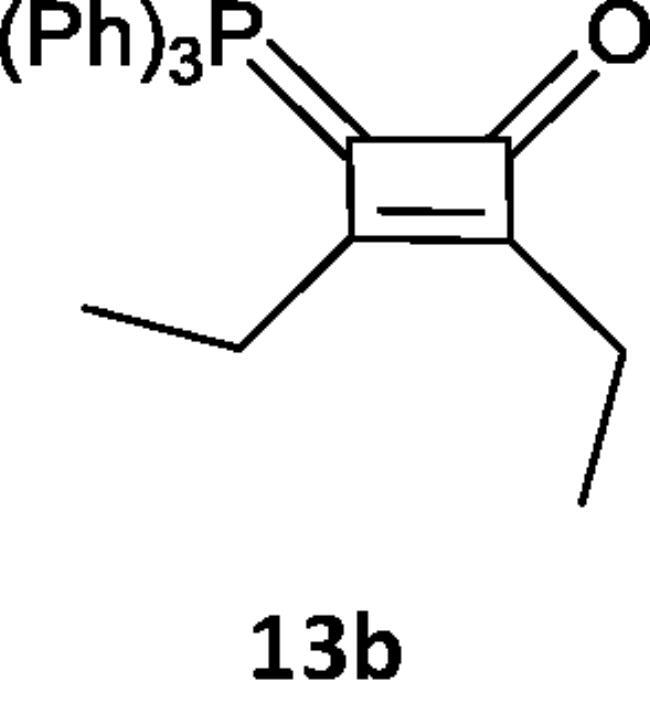	248.82 ± 10.4
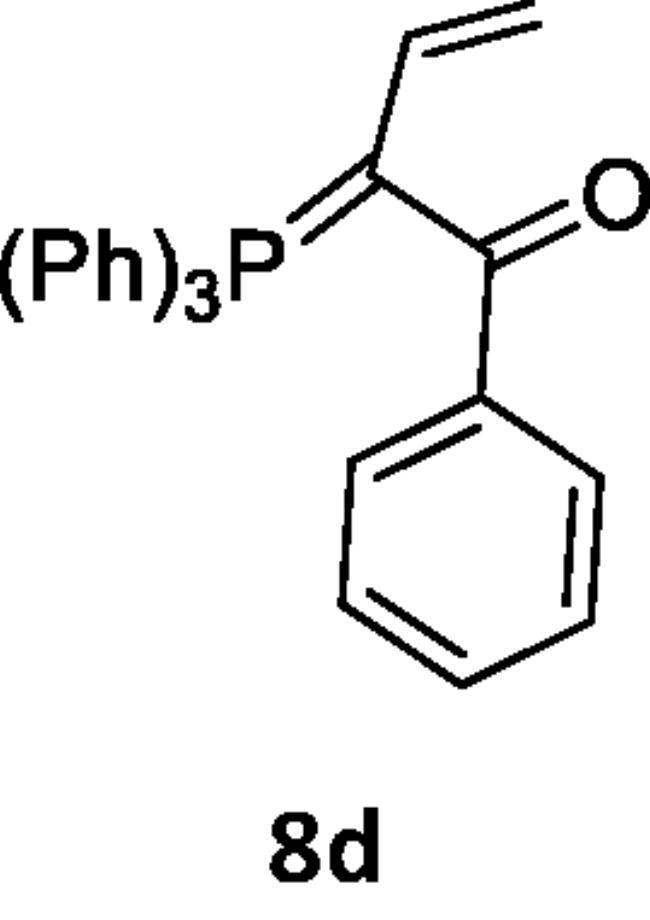	426.35 ± 21.5	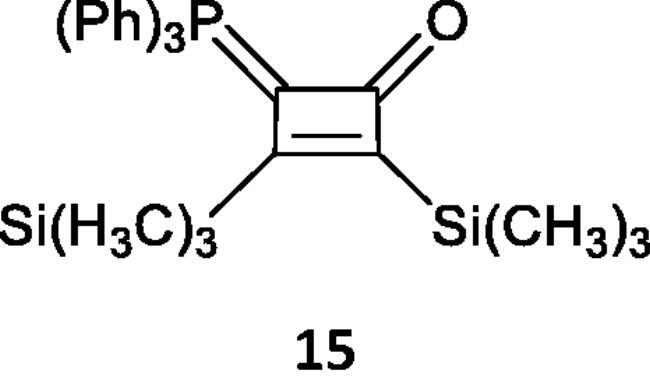	113.05 ± 4.82
**Donepezil**	34.42 ± 1.39		

^a^Average of triplicate determination.

Based on the obtained results, compound **8c** showing the highest activity against AChE enzyme was subjected for further investigation (Table 1).

In order to test the selectivity of the selected compound, the inhibitory activity of compound **8c** against BuChE enzyme was tested and compared to that of the reference compound, donepezil. This was carried out by using the modified Ellman spectrophotometric method. The results are summarised in (Table 2). As can be seen, compound **8c** did not show a remarkable selectivity for AChE enzyme more than BuChE and thus it can be considered as non-selective cholinesterase inhibitor.

**Table 2.** BuChE IC_50_, selectivity index, MMP-2 IC_50_, and self-induced Aβ_1–42_ aggregation IC_50_.

Compound	BuChE IC_50_ (nM) ± *SD*	Selectivity index	MMP-2 IC_50_ (nM) ± *SD*	Self-induced Aβ_1–42_ aggregation IC_50_ (nM) ± *SD*
8c	84.83 ± 2.91	0.87	724.19 ± 16.5	302.36 ± 11.41
Donepezil	66.47 ± 2.51	1.93	–	115.94 ± 3.87
Imatinib	–	–	328.76 ± 12.1	–

Selectivity index = (BuChE IC_50_)/(AChE IC_50_).

Matrix Metalloproteinase-2 (MMP-2) is known for its impact on neurological disorders as AD^44–46^. Accordingly, compound **8c** was tested for MMP-2 inhibition and compared to imatinib as reference compound, but it showed IC_50_ of 724.19 nM, which is relatively higher than that for imatinib (328.76 nM) showing a weaker inhibition ability (Table 2).

Eventually, self-induced Aβ_1–42_ aggregation was tested for **8c** derivative, which showed IC_50_ of 302.36 nM while the reference compound donepezil exhibited IC_50_ of 115.94 nM (Table 2).

From the above-mentioned results, it can be concluded that compound **8c** is a non-selective AChE/BuChE inhibitor with moderate activity against Aβ_1-42_ aggregation and weak activity against MMP-2 enzyme.

#### Kinetic study for AChE inhibition of compound 8c

**Figure 5.**
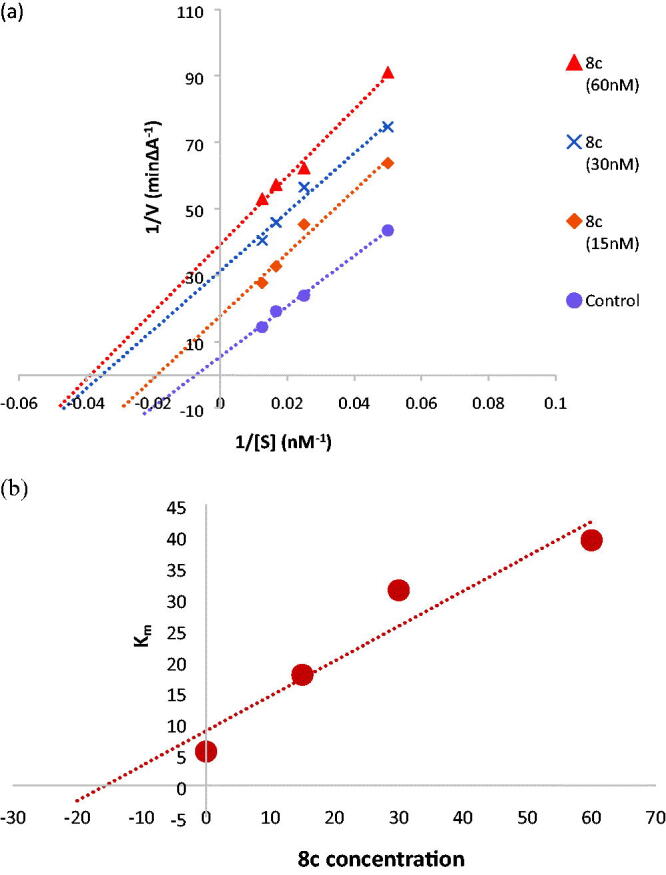
(a) Kinetic study on the mechanism of AChE inhibition by compound **8c**, Overlaid Lineweaver-Burk reciprocal plots of AChE initial velocity at increasing substrate concentration (15–60 nM) in the absence and in the presence of different concentrations of **8c.** (b) Dixon plot of compound **8c** showing the *Ki* value as negative intercept on the *X*-axis.

To further understand the mechanism of AChE inhibition, the enzyme kinetics parameters of compound **8c**, the most effective AChE inhibitor, were examined. When the concentration of the inhibitor was increased, the Lineweaver-Burk double reciprocal plot (1/*V* vs. 1/*S*) revealed decreasing *K_m_* and decreasing *V*_max_ values for both inhibited and uninhibited enzymes (Figure 5(a)). The Lineweaver-Burk plot and *V*_max_ and *K_m_* patterns suggested an uncompetitive type of inhibition. Moreover, based on the Dixon plot with the *K_i_* value as a negative intercept on the *X*-axis, the plot of the *K_m_* versus different concentrations of **8c** produced an estimate of the inhibitory constant, *K_i_*, of 15.7 nM (Figure 5(b)).

#### SH-SY5Y neuroblastoma cell toxicity of compound 8c

**Figure 6.**
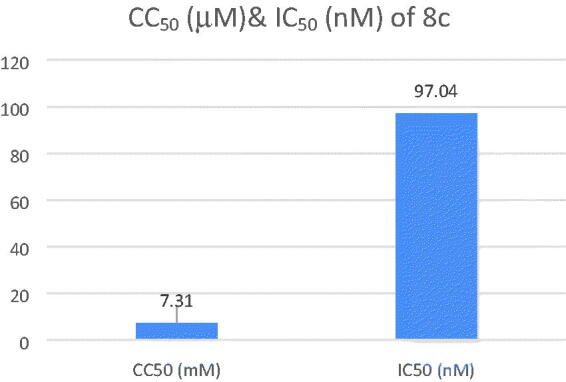
CC_50_ and IC_50_ of compound **8c**.

The safety of compound **8c** was assessed by testing its cytotoxicity effect on human neuroblastoma cell line SH-SY5Y. The cell viability was determined through using MTT assay. Compound **8c** showed a low cytotoxicity with CC_50_ value of 7.31 ± 0.26 µM with high AChE inhibition activity (IC_50_ = 97.04 nM) and so, resulting in a high selectivity index (SI, CC_50_ (nM)_/_IC_50_(nM)) of 75.33, proving its safety on normal cell lines (Figure 6).

### Behavioural studies

The most important criterion for assessing anti-AD medication candidates is cognitive improvement. Compound **8c’**s investigation in AD animal models was driven by promising *in vitro* results. In order to test potential anticholinergic medication candidates, the *in vivo* scopolamine model was employed to cause memory impairment in mice. Scopolamine can inhibit the cholinergic system by antagonising the muscarinic receptor^47^. Then, using donepezil as a positive control, the effects of compound **8c** on cognitive improvement were assessed using Y-Maze and passive avoidance tests.

#### Y-maze test

**Figure 7.**
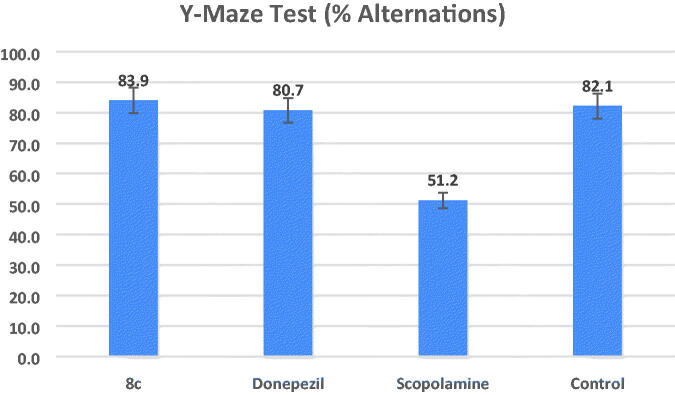
Effects of compound **8c** on the % of spontaneous alternations in the Y-maze test compared to the reference drug donepezil, the data shown are mean ± SD (*n* = 5). ^#^*p* < 0.01 vs. control group.

Animal working memory is assessed using the hippocampal-dependent Y-maze test. Working memory is measured using the spontaneous alternation score^48^. Figure 7 demonstrates that the proportion of alternations was considerably lower in the scopolamine model group than in the control group (percent alternations = 51.2 and 82.1, respectively, at ^#^*p* = 0.01). At a dose of 1 mg/kg for each, the mice group treated with compound **8c** demonstrated an increase in the percent alternations (percent alternations = 83.9 (*p = 0.01)) that is non-significantly different from the increase in the donepezil-treated group (percent alternations = 80.7 (*p = 0.01)), but this increase in the percentage of alternations was significantly different to the percentage of the model group.

#### Passive avoidance test

**Figure 8.**
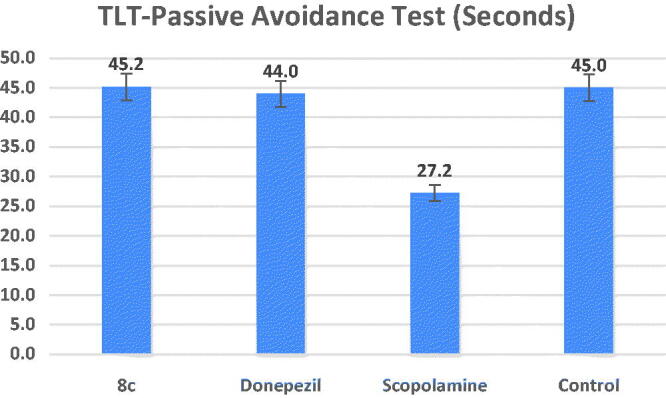
Effects of compound **8c** on the TLT in seconds in the step-down passive avoidance test by the scopolamine-induced cognitive impairment compared to the reference drug donepezil, the data shown are mean ± SD (*n* = 5). ^#^*p* < 0.01 vs. control group.

The central cholinergic neurons have a role in passive avoidance learning, while the hippocampus is principally in charge of controlling the learning response^49^. The step-down passive avoidance test was used to assess compound **8c’**s ability to guard against memory loss. Figure 8 shows that the transfer latency time (TLT) of the scopolamine-treated group was substantially shorter than that of the control group (27.2 s, at ^#^*p* = 0.01). Comparable to donepezil at a dose of 1 mg/kg for each, treatment with compound **8c** (TLT = 45.2 s) demonstrated a substantial increase in TLT compared to the model group (*p = 0.01). These *in vivo* results demonstrated that compound **8c** could cross the blood–brain barrier (BBB), hence enhancing scopolamine–induced cognitive impairment.

### In silico studies

#### In silico prediction of BBB permeability of compound 8c

**Figure 9.**
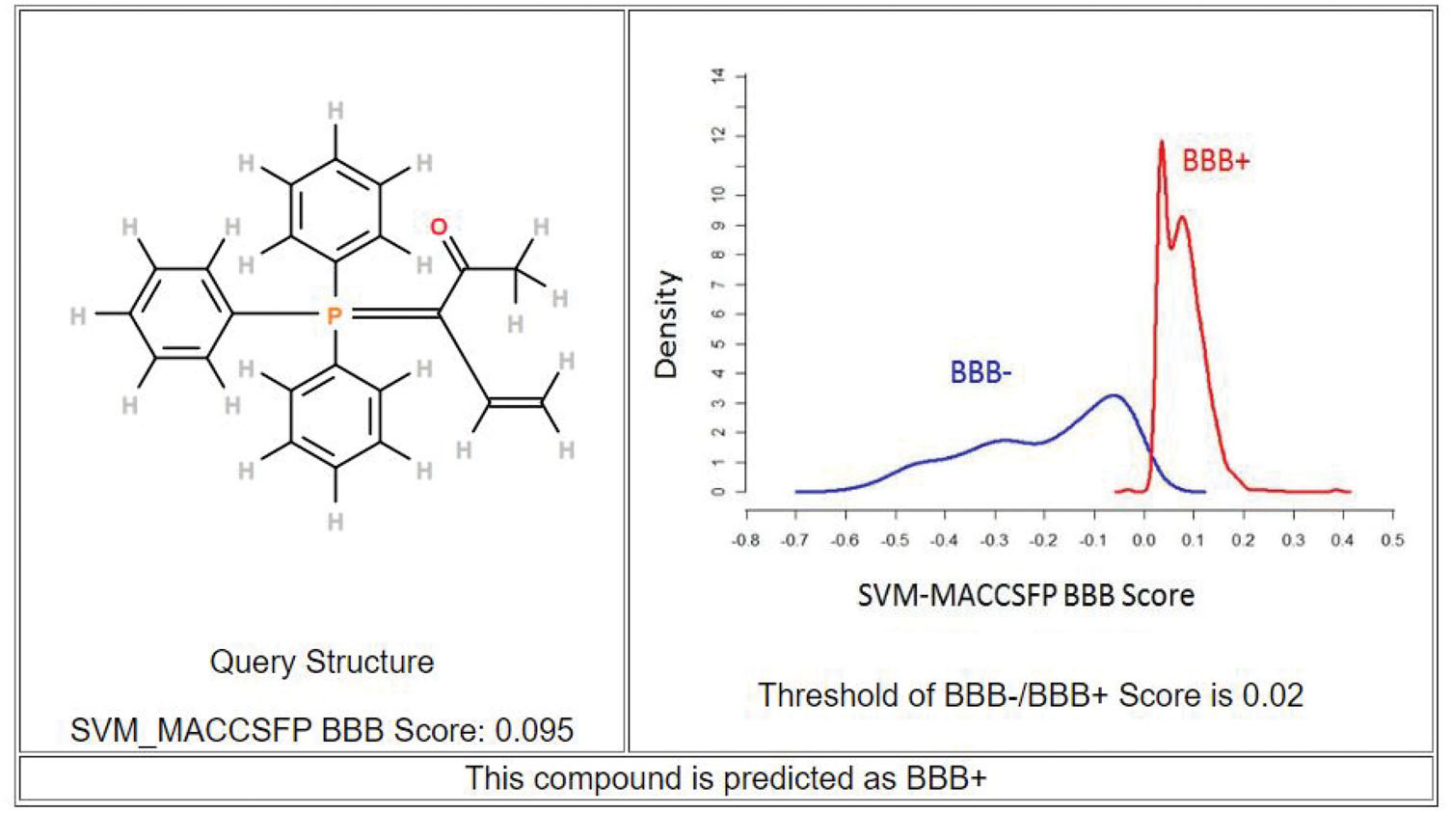
Prediction of BBB permeability (pkCSM webserver).

**Figure 10.**
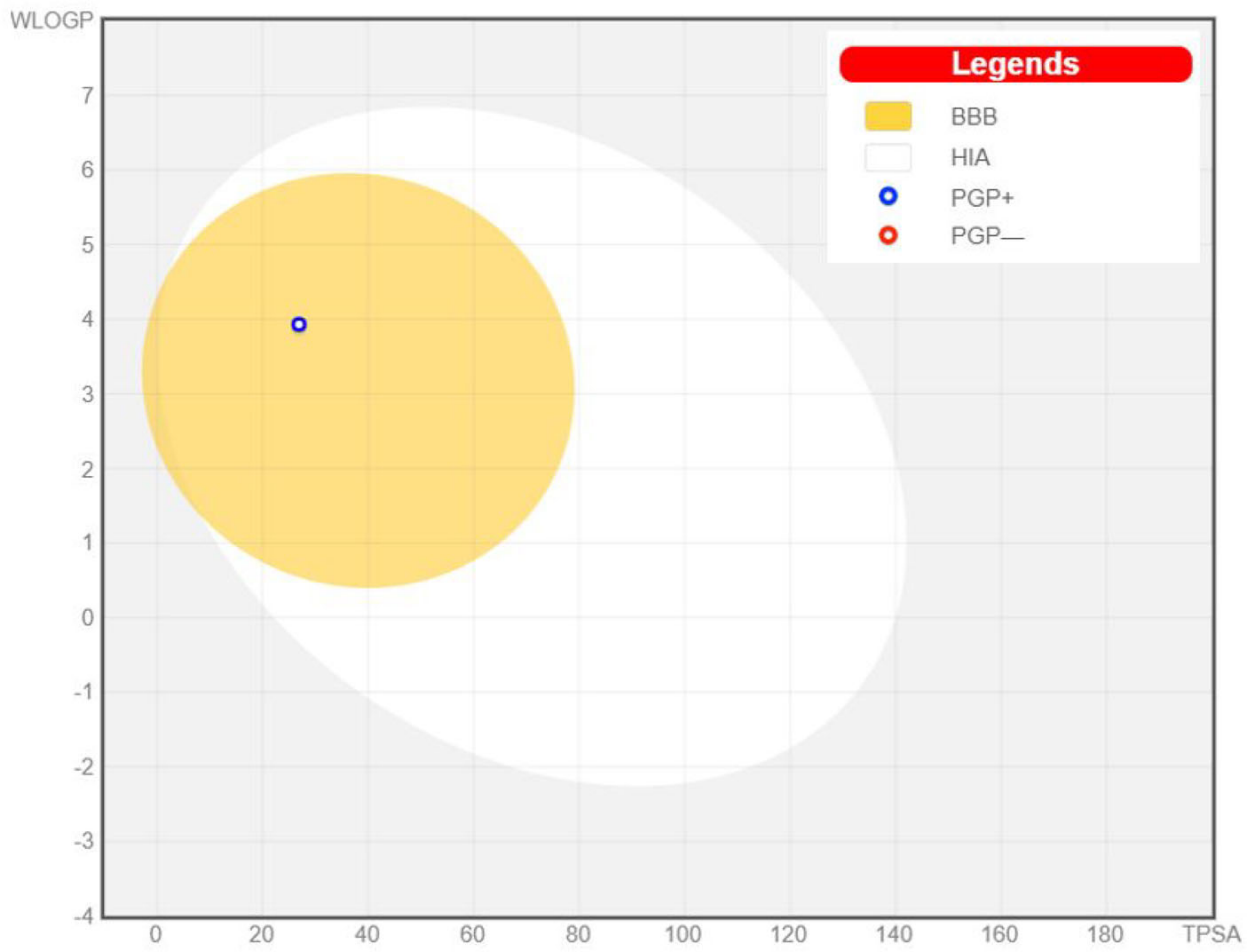
Prediction of BBB permeability (SwissADME webserver).

Drugs that target the CNS must first cross the BBB. The inability of therapeutic molecules to penetrate the BBB is a key obstacle for CNS drug candidates and needs to be addressed quickly in the drug development process. Predicting BBB permeability of new CNS drugs is therefore crucial^50^. Two webservers were used to predict the BBB permeability, pkCSM^51^ and SwissADME^52^. Both servers confirm that compound **8c** can penetrate BBB and this facilitates its action inside the CNS for treating AD (Figures 9 and 10).

#### Molecular docking

The molecular docking analysis was conducted using the X-ray crystallographic structure of human AChE in contact with (–)-galantamine, which was downloaded from protein data bank (PDB) (ID: 4EY6)^53^. The protein structure of human BuChE in complex with its co-crystallized ligand from PDB ID; 7AIY^54^.

The co-crystallized ligand (galantamine) was re-docked into the same binding site as part of the validation stage in order to confirm the tested compounds’ actual binding mechanism. The binding energy result for galantamine was −14.99 kcal./mol. The validity of the docking process is demonstrated by the RMSD value of 0.78. An aromatic gorge, catalytic triad (CT), PAS, omega loop (OL), oxyanion hole (OH), anionic subsite (AS), and acyl binding pocket are some of the key regions of the AChE active site. Ser203, His447, and Glu334 make up the CT side, while Gly120, Ala204, and Gly121 make up the OH side. Finally, AS has Gly448, Glu202, Ile451, Tyr133, and Trp86. PAS has residues at Trp286, Tyr124, Asp74, Ser125, Tyr341, and Tyr337^55^.

**Figure 11.**
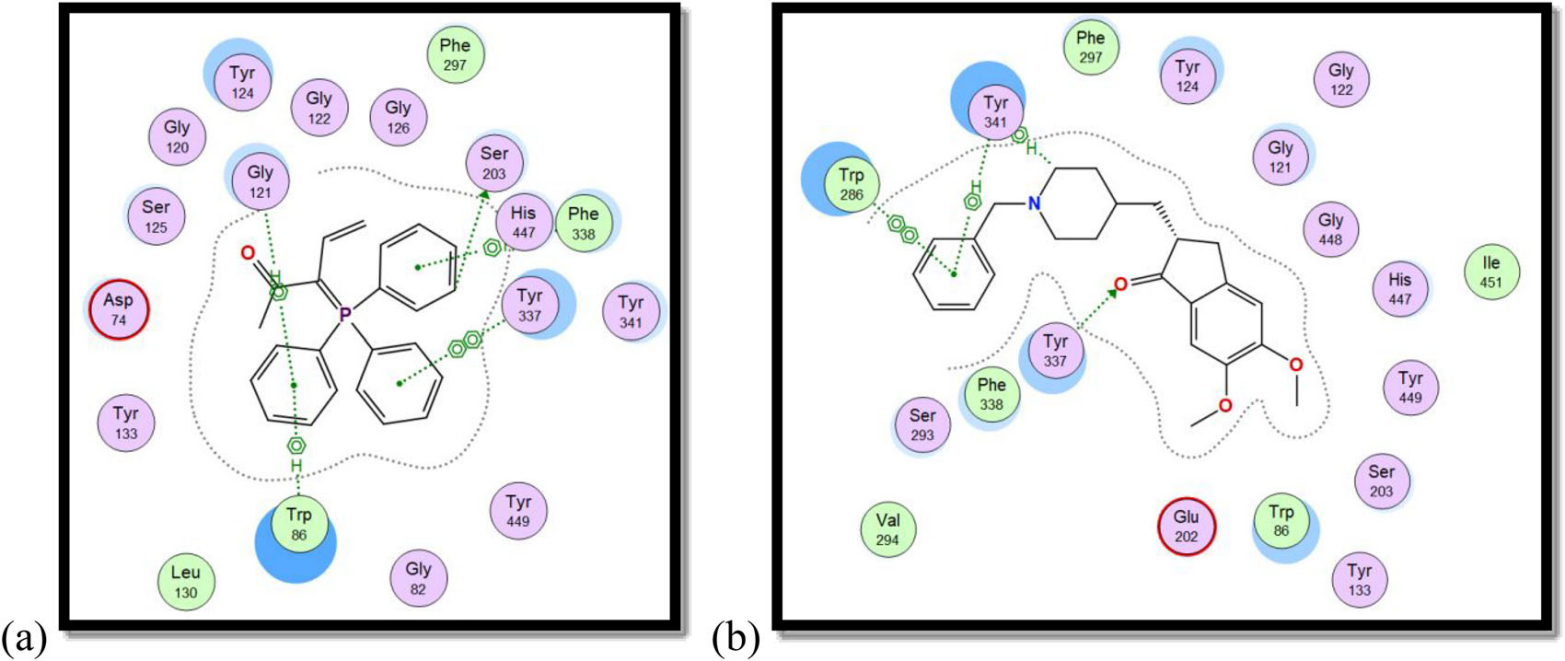
2D interaction of compound **8c** (a) versus donepezil (b) with AChE active site.

**Figure 12.**
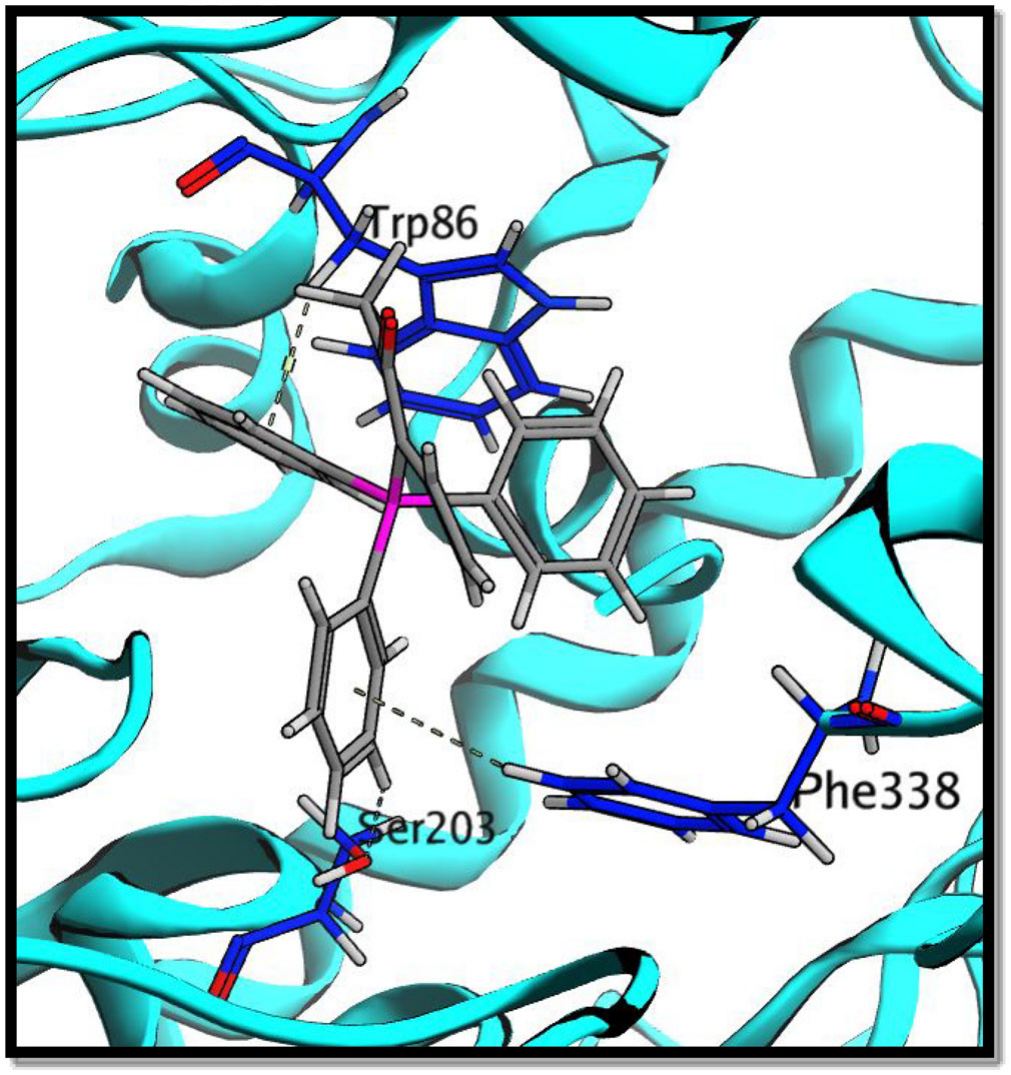
3D interaction of compound **8c** with AChE active site.

Compound **8c** showed a good binding score (–14.81 kcal/mol) compared to that of donepezil (–15.23 kcal/mol); the reference AChE inhibitor. **8c** derivative with three phenyl substitutions could bind through Arene-H interaction with Trp86 (AS), Gly121 (OH site), and Phe338 residues. Moreover, **8c** binds through arene–arene interaction with Tyr337 (PAS) and non-classical hydrogen bond with Ser203 (CT). On the other hand, donepezil showed hydrogen bond interaction with Tyr337 (PAS) through its carbonyl oxygen in addition to arene interactions with Tyr341 (PAS) and Trp286 (AS), Figures 11 and 12.

**Figure 13.**
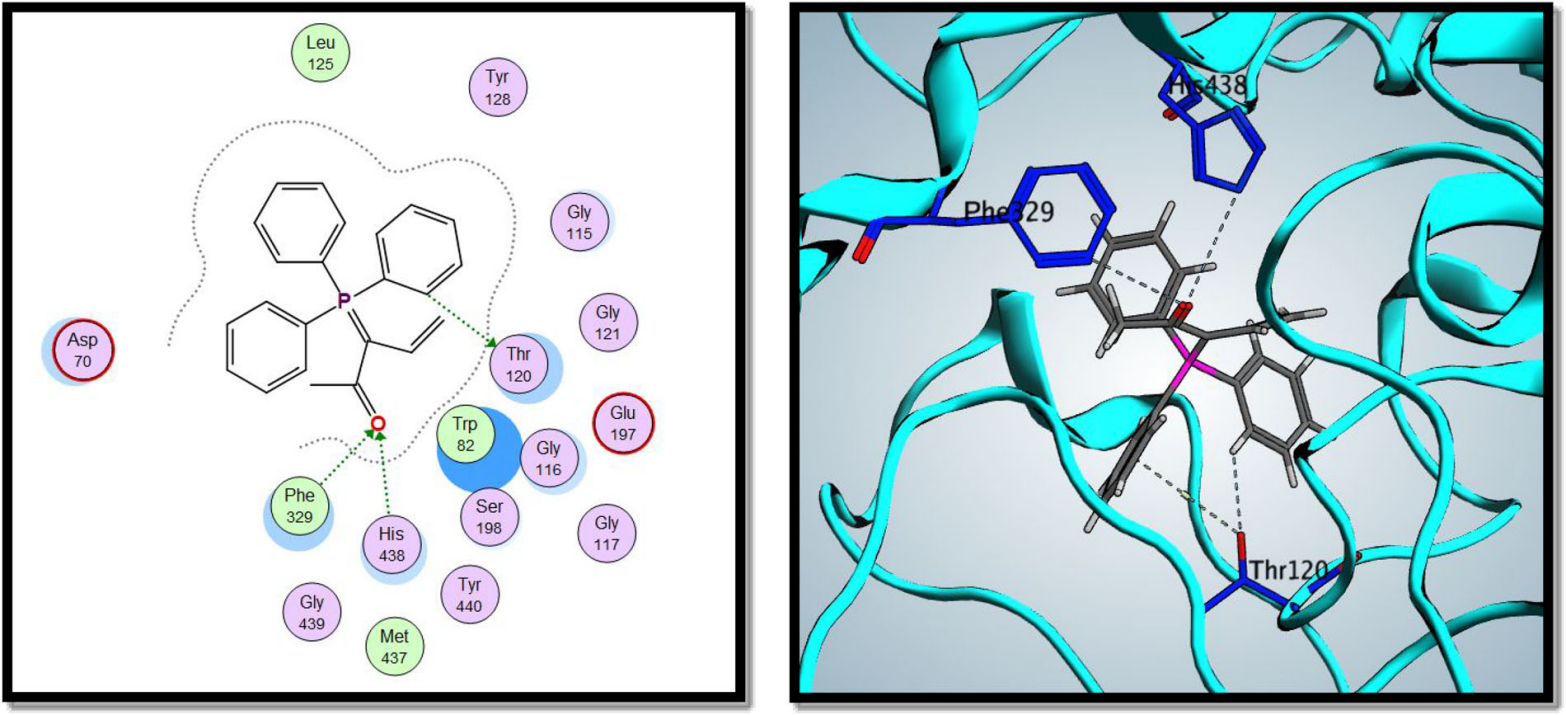
2D and 3D interactions of compound **8c** with BuChE active site.

On the other hand, compound **8c** showed good binding energy score with BuChE active site (–10.24 kcal/mol) with hydrogen bonding with Thr120, Phe329, and His438 amino acid residues (Figure 13). So, it was clear from the docking study that compound **8c** showed good binding affinity to AChE and BuChE enzymes, which confirms its good biological activity against both enzymes.

## Conclusion

Searching for new chemical compounds that target one of the major neurodegenerative diseases; AD; is always a major concern for chemistry researchers. Focussing on phospharnylidene derivatives which had been proved to have good anti-Alzheimer activity and synthesising ten new derivatives in a hope to find a good candidate for AD. Biological evaluation of the synthesised derivatives testing their anti-AChE inhibitory activity showed good results particularly for the acetylphrosporanyl derivative **8c** which showed also promising results either in *in vivo* behavioural tests or in *in silico* studies makes it a newly discovered candidate for AD therapy that can be furtherly improved in future works.

## Experimental

### Chemistry

#### Reaction of dichloro-methyl-vinyl-silane (1) with phosphacumulenes (2a) or (2 b)

At room temperature, dry THF (30 mL) containing 0.14 g (0.001 mol) of compound 1 and 0.001 mol of phosphacumulenes (**2a**) or (**2b**) was stirred for 6 h. For (**2a)** compound, the time was extended to 8 h while for (**2b)**, the stirring was continued until no starting material was detected by TLC. Under reduced pressure, THF was distilled off and then the formed residue was purified by using silica gel with an elution system composed of pet. Ether (heated to 60–80 °C)/ethyl acetate (v:v, 80:20) giving (**5a**) or (**5b**), respectively.

***N-(1-chloro-1-methyl-2-(triphenylphosphoranylidene)-1H-silol-3(2H)-ylidene)aniline (5a),*** yellow crystals, yield 50%, mp 164 °C, IR (ν, cm^−1^): 1573 (C = N), 1438 (C = P). ^1^HNMR (500.1 MHz, CDCl_3_): δ 2.01 (s, 3H, CH_3_), 5.87 (d, 1H, *J*_HH_ = 10 Hz, CH), 5.95 (d, 1H, *J*_HH_ = 10 Hz, CH), 6.75–7.81 (m, 20H, arom.). ^13^C NMR (125 MHz, CDCl_3_): 16.11 (CH_3_), 117.03 (CH), 120.69 (CH), 124.30, 129.13, 129.29, 129.43, 130.21, 131.88, 132.08, 132.48, 132.51, 132.68, 133.81, 135.63, 136.80 (C – aromatics), 148.00 (C = P), 151.31 (C = N). ^31^P NMR (202.4 MHz, CDCl_3_): δ 17.30. MS: *m/z*: 484 (M^+2^). Anal. Calcd for C_29_H_25_ClNPSi (482.03): C, 72.26; H, 5.23; N, 2.91; P, 6.43. Found C, 72.00; H, 4.98; N, 2.68; P, 6.11.

***1-Chloro-1-methyl-2-(triphenylphosphoranylidene)-1H-silol-3(2H)-one (5 b),*** yellow crystals, yield 35%, mp 190 °C, IR (ν, cm^−1^): 1701 (C = O), 1435 (C = P). ^1^HNMR (500.1 MHz, CDCl_3_): δ 1.89 (s, 3H, CH_3_), 3.43 (d, 1H, *J*_HH_ = 10 Hz, CH), 6.81 (d, 1H, *J*_HH_ = 10 Hz, CH), 7.25–7.84 (m, 15H, CH aromatics). ^13^C NMR (125 MHz, CDCl_3_): 10.95 (CH_3_), 117.77(CH), 118.65 (CH), 119.78 − 135.31 (C–aromatics), 135.31 (C = P), 165.95 (C = O). ^31^P NMR (202.4 MHz, CDCl_3_): δ 19.40. MS: *m/z*: 406 (M). Anal. Calcd for C_23_H_20_ClOPSi (406.92): C, 67.89; H, 4.95; P, 7.61. Found C, 67.57; H, 4.67; P, 7.39.

#### Reaction of dichloro-methyl-vinyl-silane (1) with stabilised phosphonium ylides (6a–6d)

A solution of dichloro-methyl-vinyl-silane (**1**) (0.14 g, 0.001 mol) in dry THF (20 mL) was added to a solution of ethoxycarbonyl-(**6a**) (0.33 g, 0.001 mol) methoxycarbonyl-(**6b**) (0.34 g, 0.001 mol) acetyl-(**6c**) (0.31 g, 0.001 mol) or benzoyl-methylenetriphenylphosphoranes (**6d**) (0.38 g, 0.001 mol) in dry THF (20 mL). The reaction mixture was stirred for 12 h. The solvent was removed under reduced pressure and the remained residue was chromatographed on silica gel using pet. ether (60–80 °C)/ethyl acetate.

***Ethyl 4-(dichloro(methyl)silyl)-2-(triphenylphosphoranylidene)butanoate (7a),*** was isolated by using pet. ether (60–80 °C)/ethyl acetate (v:v, 60:40) as eluent, white crystals, yield 45%, mp 137–139 °C, IR (ν, cm^−1^): 1732 (C = O), 1438 (C = P). ^1^HNMR (500.1 MHz, CDCl_3_): δ 1.03 (s, 3 H, CH_3_), 2.51(t, 3 H, CH_3_), 4.01 (t, 2 H, CH_2_), 5.57 (q, 2H, CH_2_), 7.68–7.89 (m, 15 H, aromatics). ^13^C NMR (125 MHz, CDCl_3_): 14.22 (Si–CH_3_), 33.01 (CH_2_–CH_3_), 63.30 (CH_2_–CH_3_), 118.05 (CH_2_–CH_2_), 118.93 (CH_2_–CH_2_), 130.71 − 135.65 (C-aromatics), 165.04 (C = O). ^31^P NMR (202.4 MHz, CDCl_3_): δ 21.03. MS: *m/z*: (M^+^-TPPO). Anal. Calcd for C_25_H_27_Cl_2_O2PSi (489.45): C, 61.35; H, 5.56; P, 6.33. Found C, 59.89; H, 5.48; P, 6.25.

***Methyl 2-(triphenylphosphoranylidene)but-3-enoate (8b),*** was isolated by using pet. ether (60–80 °C)/ethyl acetate (v:v, 50:50) as eluent, white crystals, yield 65%, mp 168–170 °C, IR (ν, cm^−1^): 1720 (C = O), 1430 (C = P). ^1^HNMR (500.1 MHz, DMSO): δ 3.60 (s, 3H, OCH_3_), 5.49 (d, 2 H, *J*_HH_ = 20 Hz, CH_2_), 7.74 − 8.06 (m, 16 H, aromatics and CH). ^13^C NMR (125 MHz, CDCl_3_): 53.74 (CH_3_), 118.05 (CH = CH_2_), 119.23 (CH = CH_2_), 130.49, 130.66, 134.11, 134.25, 135.51 (C- aromatics), 135.55 (C = P), 166.02 (C = O). ^31^P NMR (202.4 MHz, CDCl_3_): δ 21.02. MS: *m/z*: 198 (M+ – TPP). Anal. Calcd for C_23_H_21_O_2_P (360.39): C, 76.65; H, 5.87; P, 8.59. Found C, 76.31; H, 5.50; P, 8.22.

***3-(Triphenylphosphoranylidene)pent-4-en-2-one (8c),*** was isolated by using pet. ether (60–80 °C)/ethyl acetate (v:v, 70:30) as eluent, yellow crystals, yield 40%, mp 249–251 °C, IR (ν, cm^−1^): 1705 (C = O), 1428 (C = P). ^1^HNMR (500.1 MHz, DMSO): δ 2.36 (s, 3H, CH_3_), 5.84 (d, 2H, *J*_HH_ = 20 Hz, CH_2_), 7.68 − 7.89 (m, 16 H, CH and aromatics). ^13^C NMR (125 MHz, DMSO): 32.06 (CH_3_), 118.78 (CH), 119.95 (CH_2_), 130.40 − 135.17 (C-aromatics), 135.21 (C = P), 201.63 (C = O). ^31^P NMR (202.4 MHz, DMSO): δ 20.67. MS: *m/z*: 183 (M^+^-TPP). Anal. Calcd for C_23_H_21_OP (344.39): C, 80.21; H, 6.15; P, 8.99. Found C, 80.19; H, 6.09; P, 8.98.

***1-Phenyl-2-(triphenylphosphoranylidene)but-3-en-1-one (8d),*** was isolated by using pet. ether (60–80 °C)/ethyl acetate (v:v, 60:40) as eluent, yellow crystals, yield 55%, mp 272–273 °C, IR (ν, cm^−1^): 1654 (C = O), 1434 (C = P). ^1^HNMR (500.1 MHz, DMSO): δ 6.44 (d, 2H, *J*_HH_ = 25 Hz, CH_2_), 7.58 − 8.26 (m, 21 H, CH aromatics and CH). ^13^C NMR (125 MHz, DMSO): 119.07 (CH = CH_2_), 120.25 (CH = CH_2_), 129.40, 129.66, 130.36, 130.53, 134.09, 134.23, 135.10, 135.14, 135.35, 135.57 (C-aromatics), 135.65 (C = P), 193.10 (C = O). ^31^P NMR (202.4 MHz, DMSO): δ 21.66. Anal. Calcd for C_28_H_23_OP (406.46): C, 82.74; H, 5.70; P, 7.62. Found C, 82.73; H, 5.69; P, 7.60.

#### Reaction of (N-phenyliminovinylidene)triphenylphosphorane (2a) and/or (2-oxovinylidene)triphenylphosphorane (2b) with 1,2-diphenylethyne (10)

In 20 mL toluene, compound (**2a**) (0.377 g, 1 mmol) or (**2b**) (0.302 g, 1 mmol), a solution of 1,2-diphenylethyne (**10**) (0.178 g, 1 mmol) in 20 mL of toluene was added in presence of PdCl_2_. Reaction mixture was boiled for 10 h for compound and 14 h for compound (2b), there is a change in colour from yellow to dark brown. The reaction progress was followed by TLC, toluene was distilled under reduced pressure and then the formed residue was purified on silica gel by using an elution system of petroleum ether (60–80 °C): ethyl acetate (30:70, v/v) forming (**11a**) and (**11b**) were isolated, respectively.

***N-(2,3-diphenyl-4-(triphenylphosphoranylidene)cyclobut-2-en-1-ylidene)aniline (11a),*** brown crystals, yield 40%, m.p. 2420 °C. IR (ν, cm^–1^): 1609 (C = N). ^31^P NMR (202.4 MHz, d6-DMSO, δ, ppm): 22.25. MS: *m/z*: 553 (M^−2^). Anal. Calcd for C_40_H_30_NP (555.65): C, 86.46; H, 5.44; N, 2.52; P, 5.57. Found C, 86.44; H, 5.42; N, 2.49; P, 5.38.

***2,3-Diphenyl-4-(triphenylphosphoranylidene)cyclobut-2-enone (11 b),*** brown crystals, yield 35%, m.p. 1170 °C. ^1^HNMR (500.1 MHz, DMSO): δ 6.78 − 8.00 (m, 25H, CH aromatics). ^31^P NMR (202.4 MHz, d6-DMSO, δ, ppm): 33.94. MS: *m/z*: 481 (M^+^). Anal. Calcd for C_34_H_25_OP (480.54): C, 84.98; H, 5.24; P, 6.45. Found C, 84.96; H, 5.20; P, 6.48.

#### Reaction of (N-phenyliminovinylidene)triphenylphosphorane (2a) and/or (2-oxovinylidene)triphenylphosphorane (2b) with hex-3-yne (12)

A solution of (**2a**) (0.377 g, 1 mmol) or (**2b**) (0.302 g, 1 mmol) in 20 mL of toluene was added to a solution hex-3-yne (**12**) (0.082 g, 1 mmol) in 20 mL of toluene in presence of PdCl_2_. The reaction mixture was boiled for 7 h in case of 2a and for 9 h in case of (**2b**) (the progress of the reaction was monitored by TLC). Under reduced pressure, toluene was distilled off and the residue was chromatographed using silica gel column using petroleum ether (60–80 °C): ethyl acetate (30:70, v/v) as eluent. Products (**13a**) and (**13b**) were isolated, respectively.

***N-(2,3-diethyl-4-(triphenylphosphoranylidene)cyclobut-2-en-1-ylidene)aniline (13a),*** yellow crystals, yield 65%, m.p. 275–2770 °C. IR (ν, cm^−1^): 1671 (C = N), 1555 (C = C). ^1^HNMR (500.1 MHz, CDCl_3_): δ 1.75 (t, 3H, CH_3_), 2.16 (q, 2H, CH_2_), 7.56–7.73 (20 H, aromatics). MS: *m/z*: 458 (M−). Anal. Calcd for C_32_H_30_NP (459.56): C, 83.63; H, 6.58; N, 3.05; P, 6.74. Found C, 83.00; H, 6.61; N, 3.03; P, 6.60.

***2,3-Diethyl-4-(triphenylphosphoranylidene)cyclobut-2-enone (13b),*** brown crystals, yield 58%, m.p. 1080 °C. ^1^HNMR (500.1 MHz, CDCl_3_): δ 1.23 (t, 3H, CH_3_), 2.09 (q, 2H, CH_2_), 7.45–7.72 (15 H, aromatics). ^31^P NMR (202.4 MHz, CDCl_3_, δ,ppm): 28.31. MS (*m/z*, %): 384 (M). C_26_H_25_OP (384). Anal. Calcd for C_26_H_25_OP (384.45): C, 81.23; H, 6.55; P, 8.06. Found C, 81.20; H, 6.50; P, 8.03.

#### Reaction of bis(trimethylsilyl)acetylene(14) with phosphacumulene (2b)

Compound **(14)** (0.17 g, 0.001 mol) was added to phosphacumulene (**2b**) (0.001 mol) and refluxed for 4 h in 30 mL of dry toluene, this was proceeded until no more of the starting material could be detected by TLC. Toluene was distilled off under reduced pressure and the remained residue was purified on silica gel using pet. ether (60–80 °C)/ethyl acetate (v:v, 20:80) as eluent.

***2,3-Bis(trimethylsilyl)-4-(triphenylphosphoranylidene)cyclobut-2-enone (15),*** yellow crystals, yield 50%, m.p. 115–1170 °C. IR (ν, cm^–1^): 1729 (C = O), 1586 (C = C). ^1^HNMR (500.1 MHz, CDCl_3_): δ 2.02, 2.06 (d, 18H, 2 Si(CH_3_)_3_), 7.28–7.78 (m, 15H, aromatics). ^31^P NMR (202.4 MHz, CDCl_3_, δ, ppm): 14.37. MS (*m/z*, %): 474 (M^+2^). Anal. Calcd for C_28_H_33_OPSi_2_ (472.71): C, 71.14; H, 7.04; P, 6.55. Found C, 70.79; H, 7.00; P, 6.60.

### In vitro *biological evaluation*

#### In vitro *AChE, BuChE and kinetic studies for AChE inhibition of compound 8c*

For AChE and BuChE inhibition experiments, Ellman *et al.* method’s was applied with a few minor adjustments^43^. According to the manufacturer’s recommendations, the QuantiChromTM AChE Inhibitor Screening Kit (IACE-100), Bioassays System, and the Cholinesterase (ChE) Activity Assay Kit, LifeSpan BioSciences, Inc., USA, were used to measure the inhibition of AChE and BuChE, respectively. The assay works by measuring the yellow hue that results from the reaction of 5,5′-dithiobis(2-nitrobenzoic acid) with thiocholine, which is created by the activity of AChE, at 412 nm. The relationship between product colour and enzyme activity after inhibition is established. Substrates included butyrylthiocholine iodide and acetylthiocholine iodide. The experiments were carried out using donepezil as the reference drug in the kit’s supplied buffer (pH 7.5). The AChE kinetics study used four different substrate doses between 12.5 and 100 nM, while synthetic **(8c)** compound was used in three concentrations between 15 and 45 nM. To study the mechanism of enzyme inhibition, line weaver-Burk reciprocal linear regression plots were created. The Dixon approach was used to calculate the Ki value by charting the slope of the lines from the double reciprocal Line-Weaver-Burk plot as a function of the test compound **(8c)** concentration^56^.

#### MMP-2 inhibition assays for compound 8c

MMP-2 inhibitor screening assay kit (Fluorometric), Abcam^®^ (ab139447) was used with different concentrations (0.1–1000 nM) of compound **8c** and imatinib as reference compound according to manufacturer’s instructions. This assay kit uses a fluorescence resonance energy transfer (FRET) peptide as a generic MMP activity indicator. In the intact FRET peptide, the fluorescence of one part is quenched by another. After cleavage into two separate fragments by MMPs, the fluorescence is recovered. Its signal can be easily read by a fluorescence microplate reader at Ex/Em = 490/525 nm.

#### Self-induced Aβ_1–42_ aggregation inhibition assay

The effectiveness of compound **8c** to prevent Aβ_1_*_–_*_42_ aggregation was evaluated using the beta amyloid 1–42 (Aβ_42_) Ligand Screening Assay Kit (Fluorometric) (K570-100), which was acquired from BioVision’s, USA. The assay relies on Thioflavin T binding to an aggregated amyloid peptide’s beta sheets to produce a strong fluorescent product (Ex/Em: 440/490 nm), which is abolished in the presence of an Aβ_1-42_ ligand and results in a reduction in fluorescence or complete loss of fluorescence.

#### Sh-SY5Y neuroblastoma cell toxicity of compound 8c

On human neuroblastoma SH-SY5Y cells, compound **8c’**s cytotoxicity was assessed using the MTT colorimetric technique. The American Type Culture Collection provided the cells (Rockville, MD). In RPMI-1640 media containing 10% foetal bovine serum, the SH-SY5Y cells were incubated at 37 °C. The chemical was applied to the cells at various doses (0.39–100 M) for 48 h. The MTT colorimetric assay’s described methodology was then used^57^.

### Behavioural studies (scopolamine-induced amnesia model)

In the current investigation, adult Swiss Albino mice (8–10 weeks old, weighing 25–30 g) from the National Research Centre were employed. For 1 week, animals were acclimated in a room with constant illumination, humidity, and temperature (22 °C ± 1 °C, 60%, and 12 h of light/dark cycle). Throughout the experiment, unlimited amounts of food and water were given. Mice were kept in open, opaque propylene cages with good ventilation and wide access to a variety of forages. The Research Ethics Committee of Cairo University gave its approval to all of the experimental methods used in both clinical and experimental studies.

Healthy mice were randomised into four groups of six mice each after a 1-week adaption period, at random, as follows: Group 1 is the control group, which received saline solution with 10% Tween 80. Groups II and III each received scopolamine, whereas Group IV received scopolamine + donepezil (Scopolamine plus compound **8c** group). One mg/kg of donepezil and one mg/kg of compound **8b** were given orally to the mice 30 min before scopolamine (3 mg/kg) was injected intraperitoneal to cause memory impairment. Following the injection of scopolamine, the behavioural study will be examined 30 min later.

#### Morris water maze test

The rat is placed in a sizable circular pool as part of the MWM navigation job, and it must use a variety of signals to locate an invisible or visible platform that will allow it to exit the water. The performance of the rats might be affected by a variety of circumstances, such as their sex, the environment in which they were grown, exposure to pharmaceuticals, etc. The rats can escape the labyrinth using one of three fundamental strategies: a praxic strategy (remembering the motions necessary to go to the platform), a taxic strategy (using visual clues to get where they are going), or a spatial approach (using distal cues as points of reference to locate themselves)^58^.

#### Y-maze test

In the Y-maze, a test of working memory, the animals’ capacity for spontaneous alternation was evaluated^49^. Three equally spaced horizontal arms measuring 120°, 45 cm long, and 16 cm high make up the Y-maze. Each mouse was put into one of the three arms of the maze and given free rein to wander from arm to arm. Both the number and order of arm inputs were recorded. The spontaneous alternation is derived from the following equation as a measure of memory performance: (Alternations/Total Arm Entries) − 2 × 100 = percent alternation. The student’s *t*-test was used to assess the data.

#### Passive avoidance test

The passive avoidance test was conducted in a device that had two independent compartments split by a sliding door. There was a connection between the illuminated chamber and a dark compartment with an electrifiable grid floor, which was free of electric impulses. Each mouse was initially placed in the lit chamber so that it could become accustomed to it. The sliding door is then opened, allowing access to the enclosed, gloomy space. The door was shut once the animal had entered the chamber entirely, and it was then shocked with an electric shock lasting two seconds (24 V, 0.5 mA). The latency time to enter the dark container was measured to gauge working memory (Training trial). Twenty-four hours after the training trial, a test trial was carried out without application of the electric foot shock^50^. Analysis of date were performed by using student’s t-test.

### In silico studies

#### In silico prediction of BBB permeability of compound 8c

Two webservers were used to predict BBB permeability of compound **8c**, pkCSM (http://biosig.unimelb.edu.au/pkcsm/prediction)^52^ and SwissADME (http://www.swissadme.ch/)^52^.

#### Molecular docking

In this study, molecular operating environment (MOE, 2019.0102) was operated for performing the molecular docking^59^, energy minimised structures were gained by applying MMFF94x force field till RMSD gradient of 0.1 kcal·mol^−1^Å^−1^ was achieved. The X-ray crystallographic structures of human AChE in complex with (–)-galantamine was downloaded from PDB with the following ID; 4EY6^54^, while the BuChE with PDB ID; 7AIY^55^. The co-crystallized ligand in each protein file was used to identify the essential binding features to the enzymes through a placing method named triangle matcher and London dG scoring algorithm.

## Supplementary Material

Supplemental Material
