# Supplementary material for: Dual-target ligand discovery for Alzheimer’s disease: triphenylphosphoranylidene derivatives as inhibitors of acetylcholinesterase and β-amyloid aggregation
Source: J Enzyme Inhib Med Chem. 2023 Jan 24;38(1):2166040. doi: 10.1080/14756366.2023.2166040 (PMC9879200; doi:10.1080/14756366.2023.2166040)
Supplement: Supplemental Material [file IENZ_A_2166040_SM8114.pdf]

Supporting Information file

Dual-target ligand discovery for Alzheimer's disease: Triphenylphosphoranylidene derivatives as inhibitors of acetylcholinesterase and β-Amyloid aggregation

Marwa El-Hussieny[a*], Mansoura A. Abd-El-Maksoud[a], Fouad M. Soliman[a], Marwa A. Fouad[b,c], Mohamed K. El-Ashrey[b*],

[a] *Organometallic and Organometalloid Chemistry Department, National Research Centre, 33 ElBohouth St., (Former El Tahrir) Dokki, 12622, Giza, Egypt.*

[b] *Pharmaceutical Chemistry Department, Faculty of Pharmacy, Cairo University, Kasr El-Aini St., Cairo, 11562, Egypt.*

[c] *Pharmaceutical Chemistry Department, School of Pharmacy, NewGiza University, Newgiza, km 22 Cairo-Alexandria Desert Road, Cairo, Egypt.*

S1. Experimental

S1.1. Chemistry

Melting points were determined with an electro thermal digital melting point apparatus (Electro-Thermal Engineering Ltd., Essex, United Kingdom). The IR spectra were recorded in KBr disks on a Pye Unicam SP 3300 and Shimadzu FT IR 8101 PC Infrared Spectrophotometers (Pye Unicam Ltd. Cambridge, England and Shimadzu, Tokyo, Japan, respectively). ^{1}H and ^{13}C NMR spectra were obtained from a Jeol ECA 500 MHz NMR Spectrometer (Tokyo, Japan) using deuterated dimethylsulphoxide (d6-DMSO) as a solvent and (TMS) as an internal reference at 500, 125 MHz, respectively and ^{31}P NMR spectra were obtained from a Jeol ECA 500 MHz NMR Spectrometer at 200 MHz. Mass spectra (EI-MS) were obtained with ISQ (Single Quadrupole MS, Thermo Scientific). Elemental analyses (C, H, N) results were recorded with Elementar Vario EL Germany, phosphorus was measured by spectrophotometric methods. The recorded yields are of pure isolated materials obtained by column chromatography silica gel 60 (Merck) and thin layer chromatography (TLC) which was performed on Merck Kiesel gel F254 precoated plates (Merck, Darmstadt, Germany).

S2. Copies of IR, ^{1}H NMR, ^{13}C NMR and ^{31}PNMR spectra of final compounds

Information Classification: General

^{1}H NMR , ^{13}C NMR , ^{31}PNMR and IR compound 5a

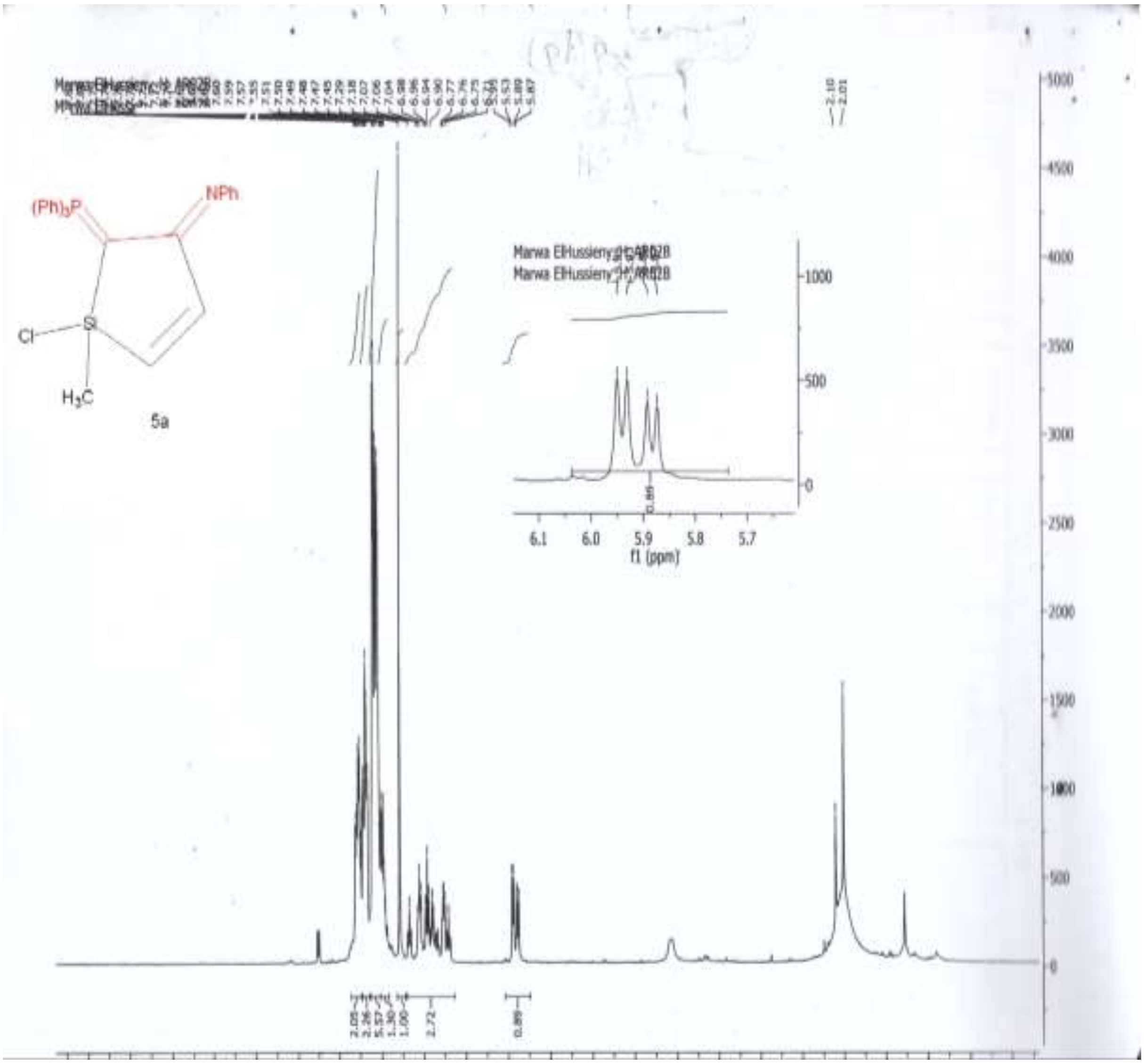

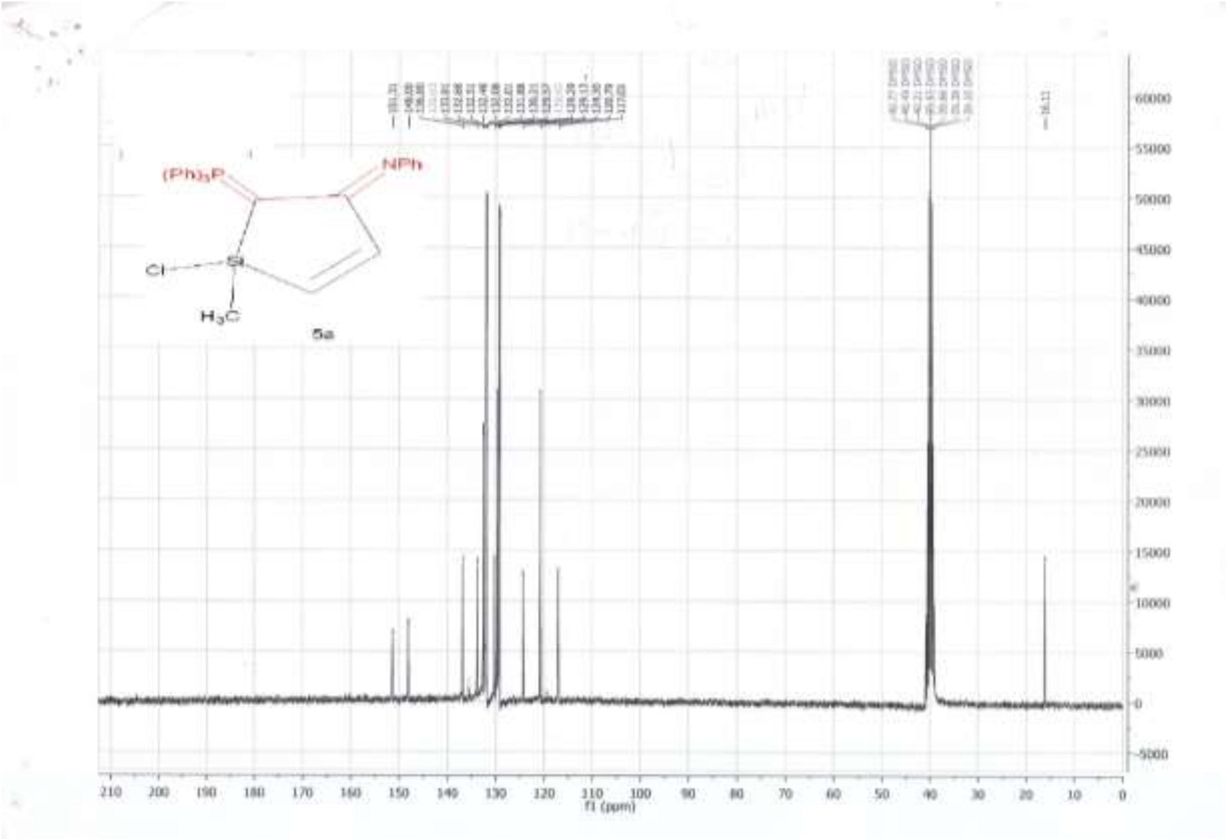

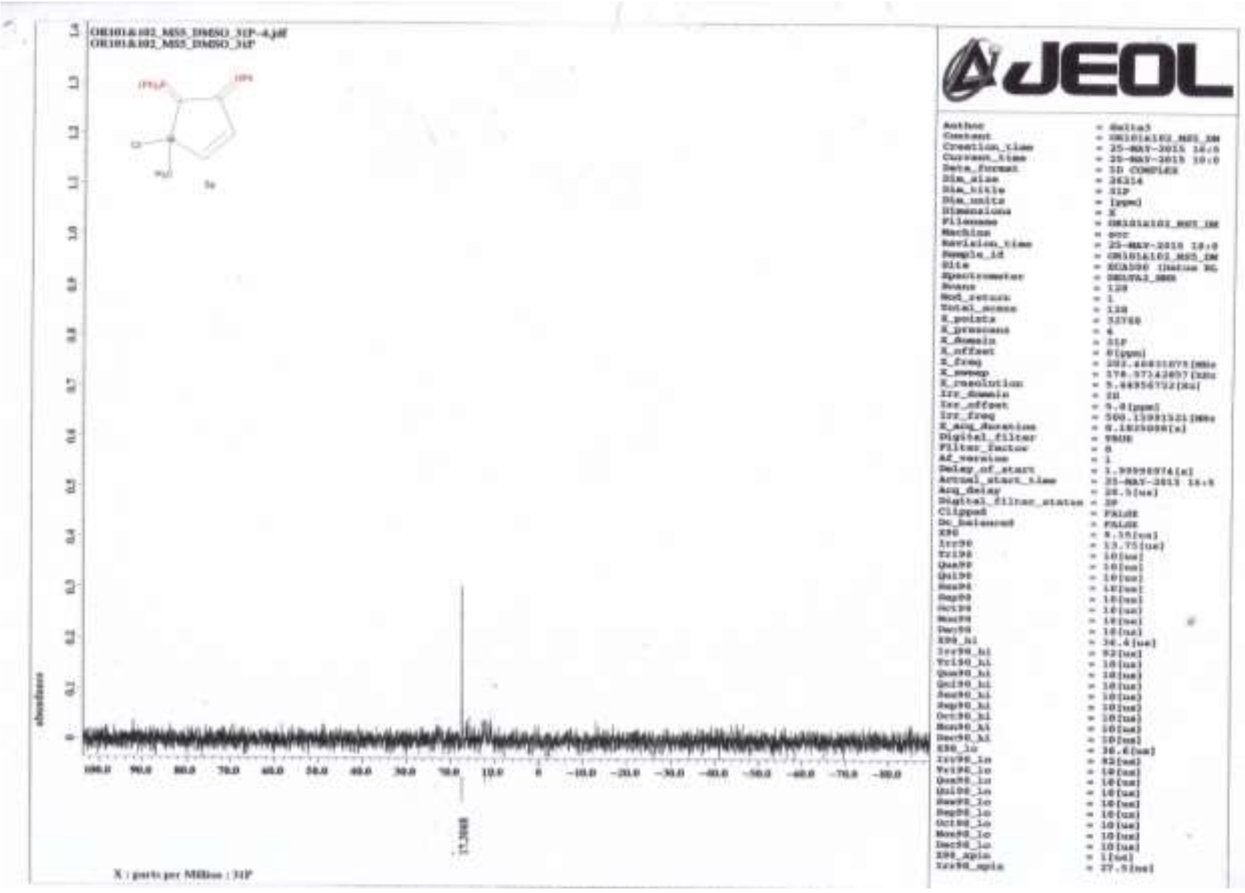

Information Classification: General

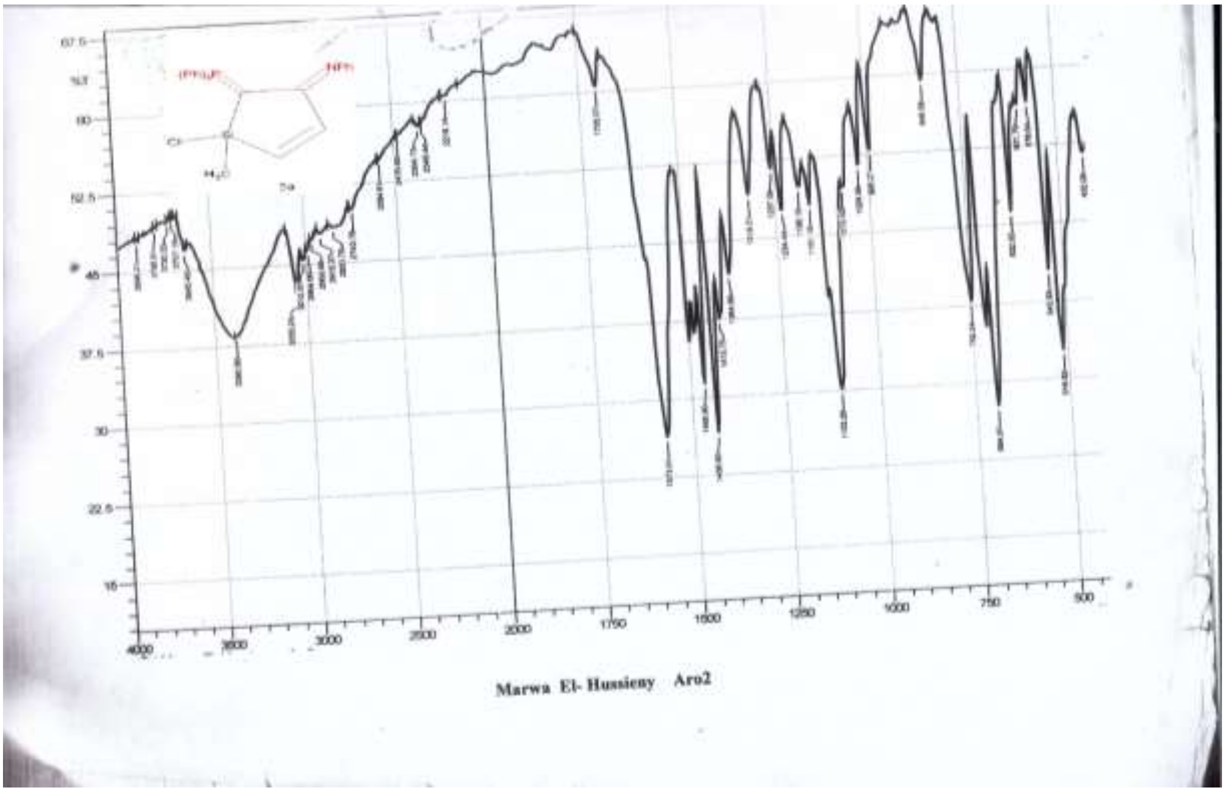

^{1}HNMR, ^{31}PNMR and IR compound 5b

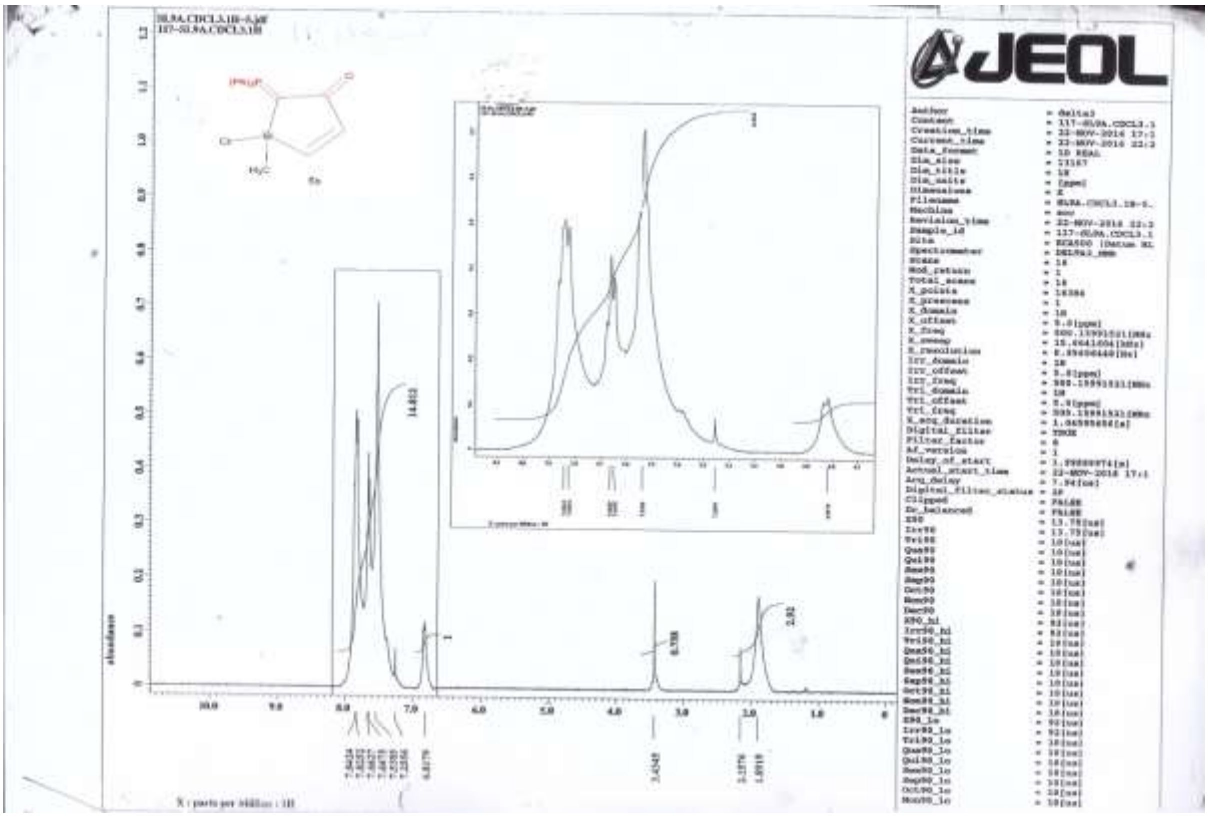

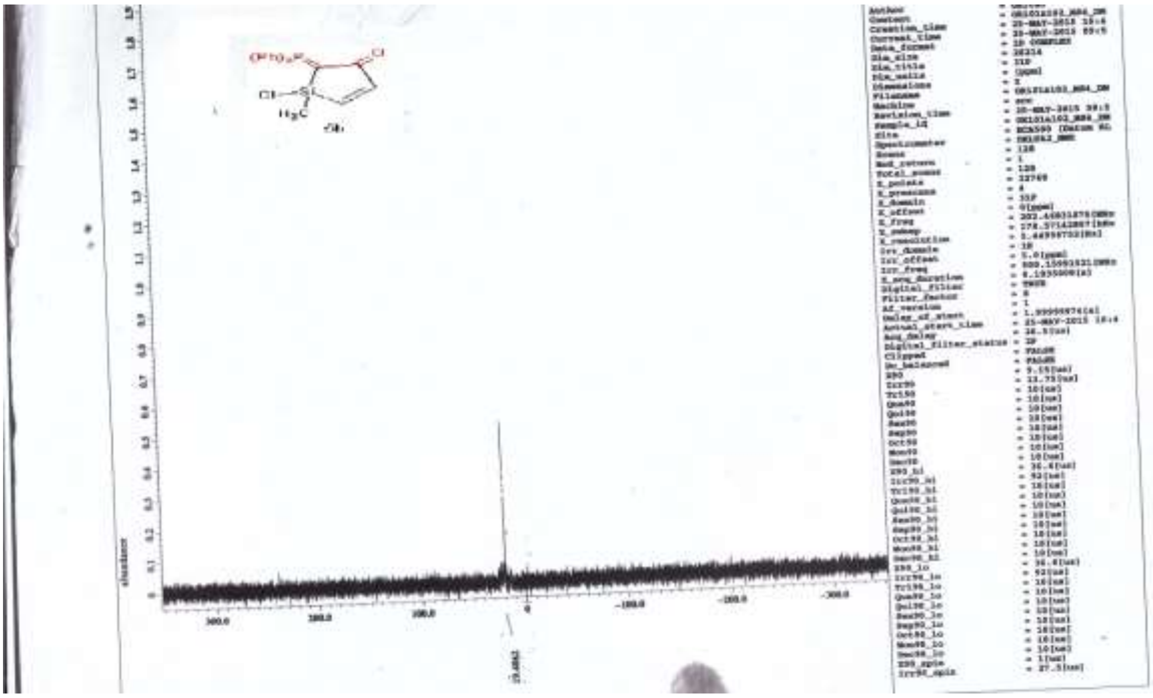

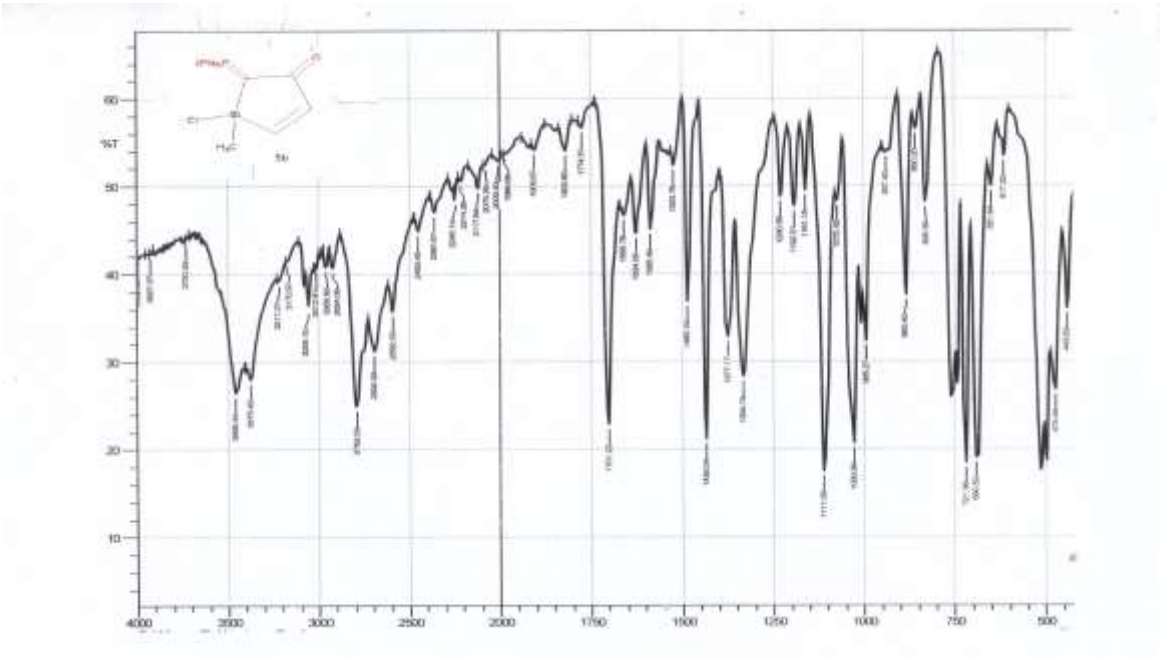

^{1}HNMR, ^{13}C NMR and IR compound 7a

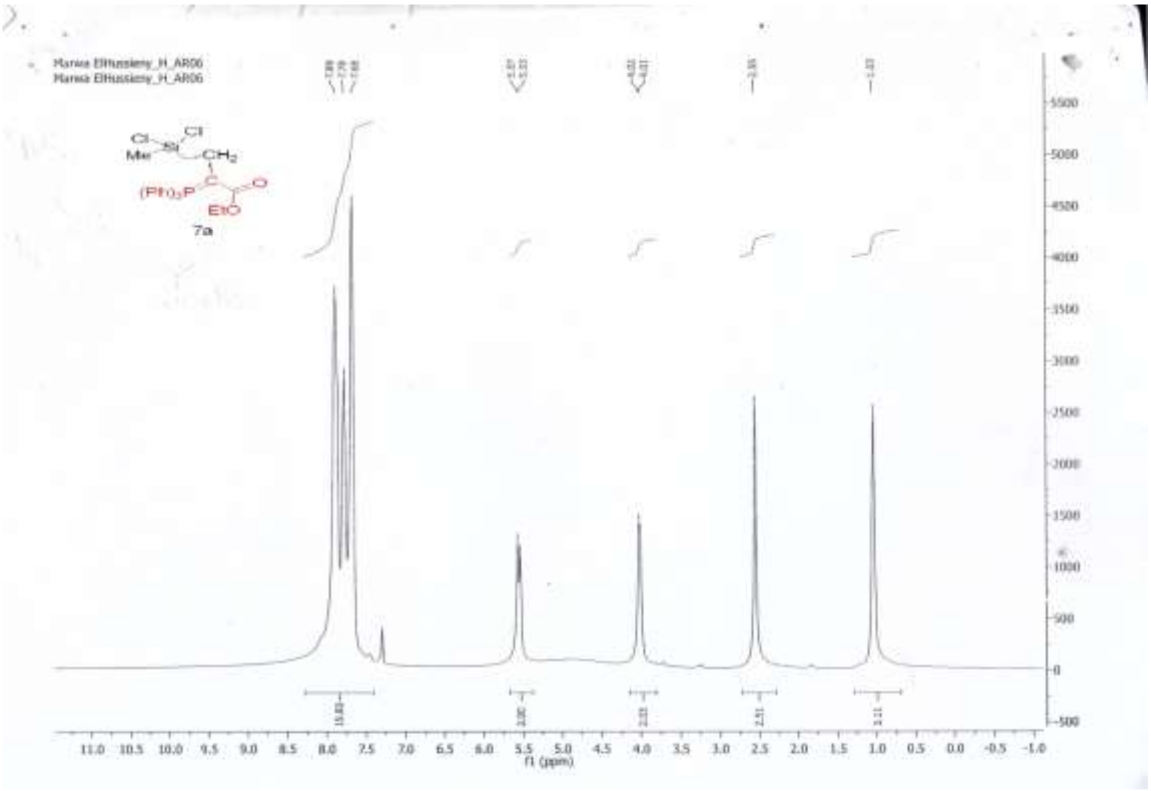
Marwa ElHussieny_H_AR06
Marwa ElHussieny_H_AR06
7a
f1 (ppm)

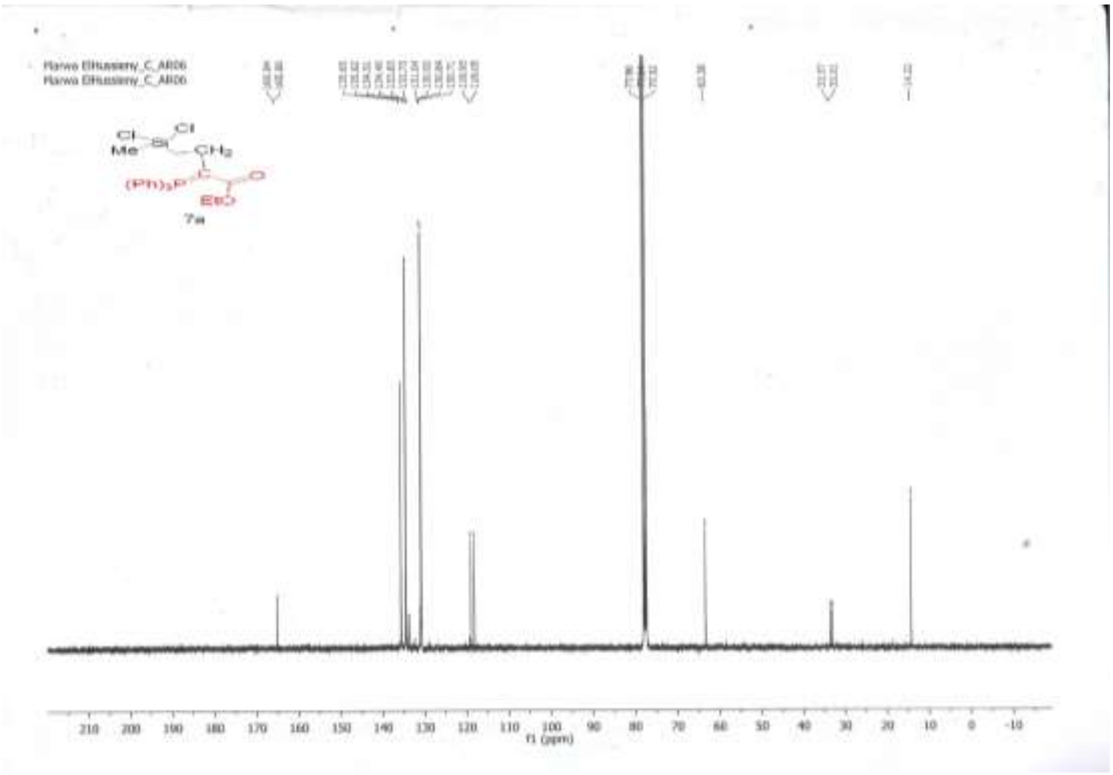
Marwa ElHussieny_C_AR06
Marwa ElHussieny_C_AR06
7a
f1 (ppm)

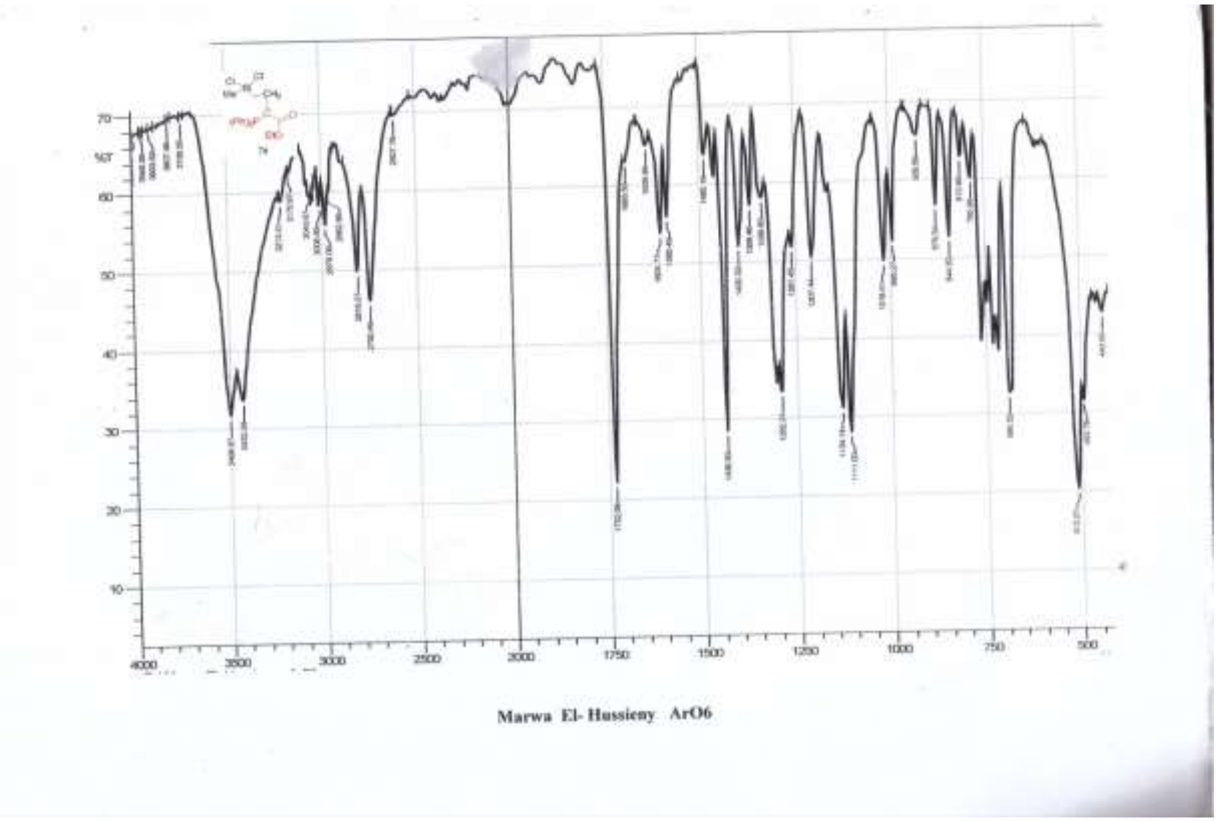

^{1}HNMR, ^{13}C NMR , ^{31}PNMR and IR compound 8b

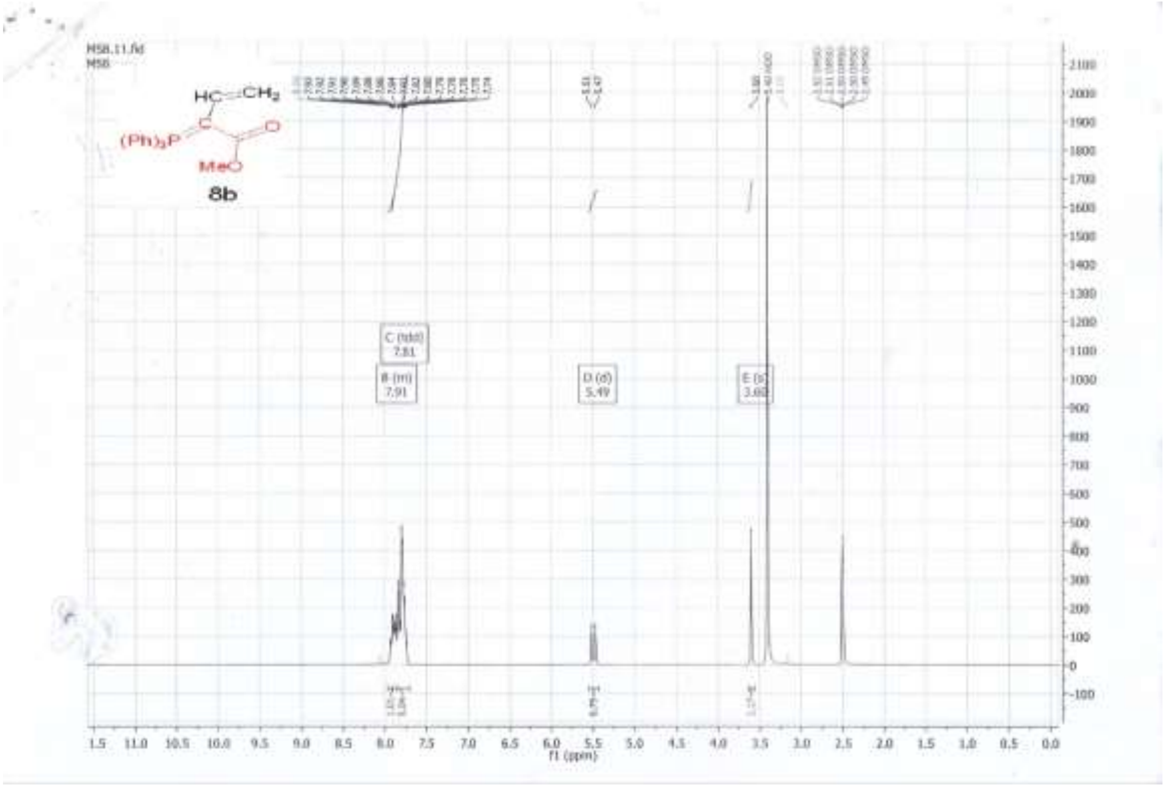

8b

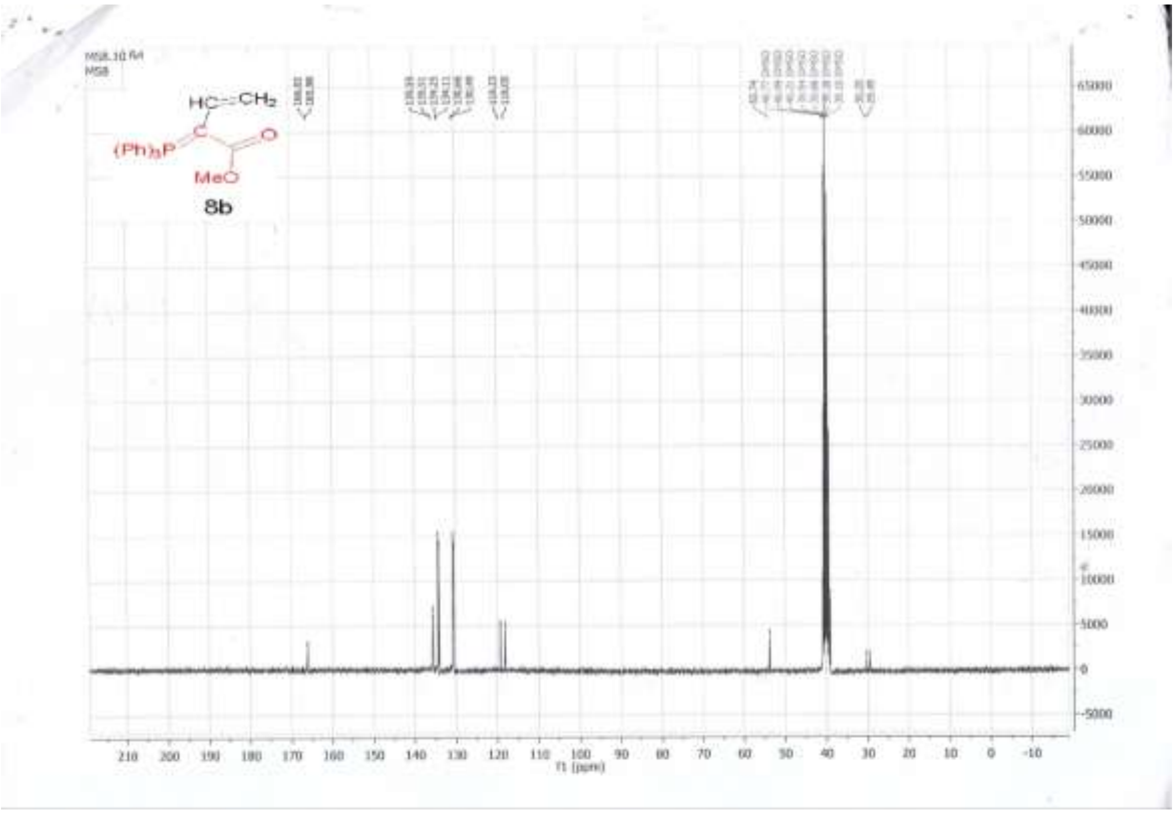

8b

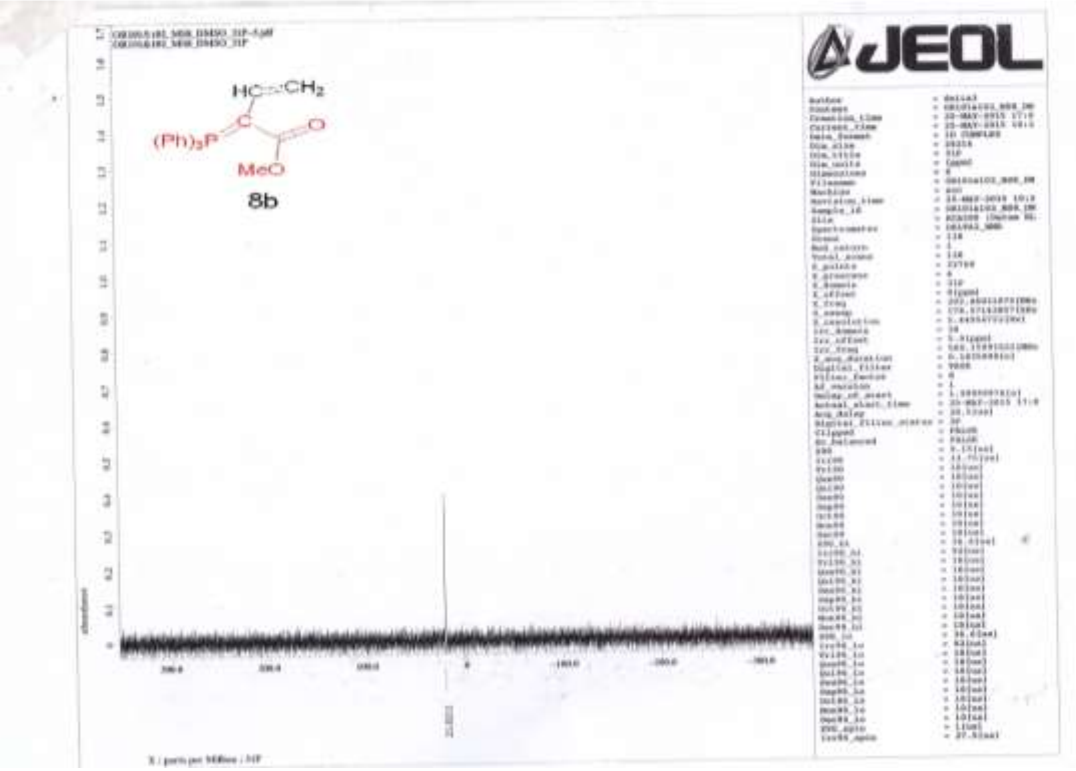

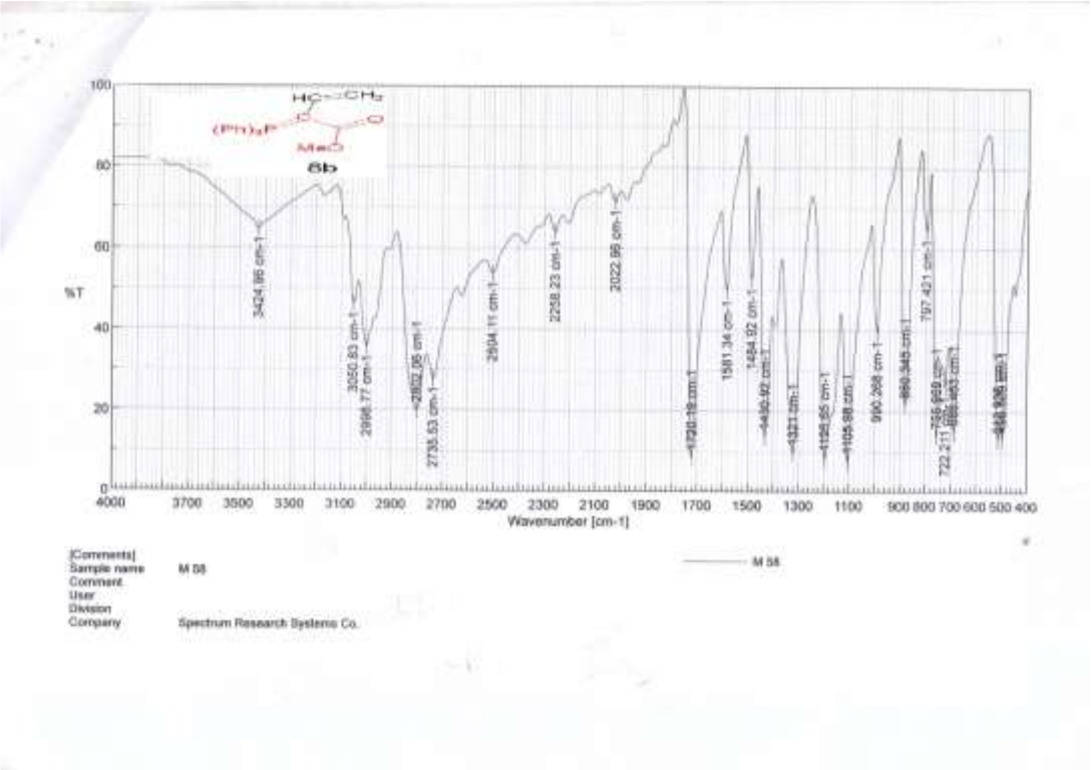

IR, 1HNMR, 31PNMR, 13C NMR Compound 8c

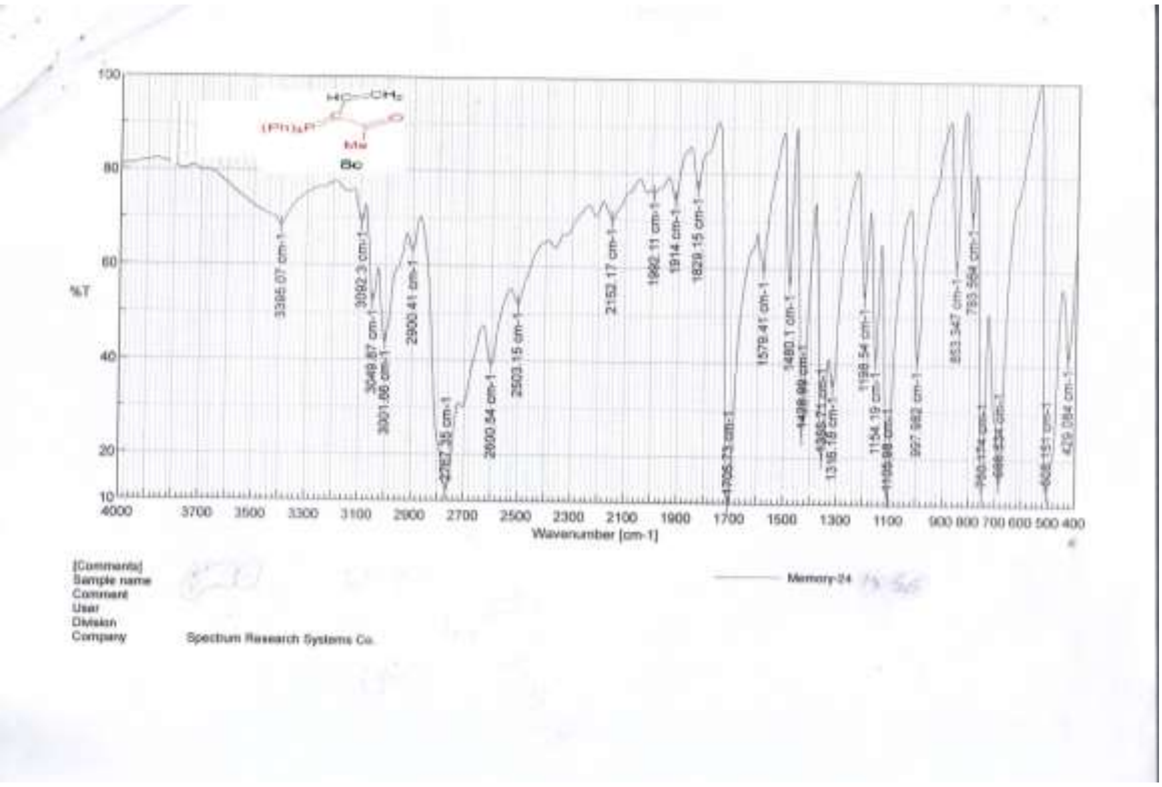
8c
%T
Wavenumber [cm-1]
3395.07 cm-1
3092.3 cm-1
3049.87 cm-1
3001.66 cm-1
2900.41 cm-1
2767.35 cm-1
2600.54 cm-1
2503.15 cm-1
2152.17 cm-1
1982.11 cm-1
1914 cm-1
1829.15 cm-1
1705.73 cm-1
1579.41 cm-1
1480.1 cm-1
1436.99 cm-1
1316.18 cm-1
1198.54 cm-1
1154.19 cm-1
997.982 cm-1
853.347 cm-1
793.564 cm-1
429.084 cm-1
[Comments]
Sample name
Comment
User
Division
Company
Spectrum Research Systems Co.
Memory-24

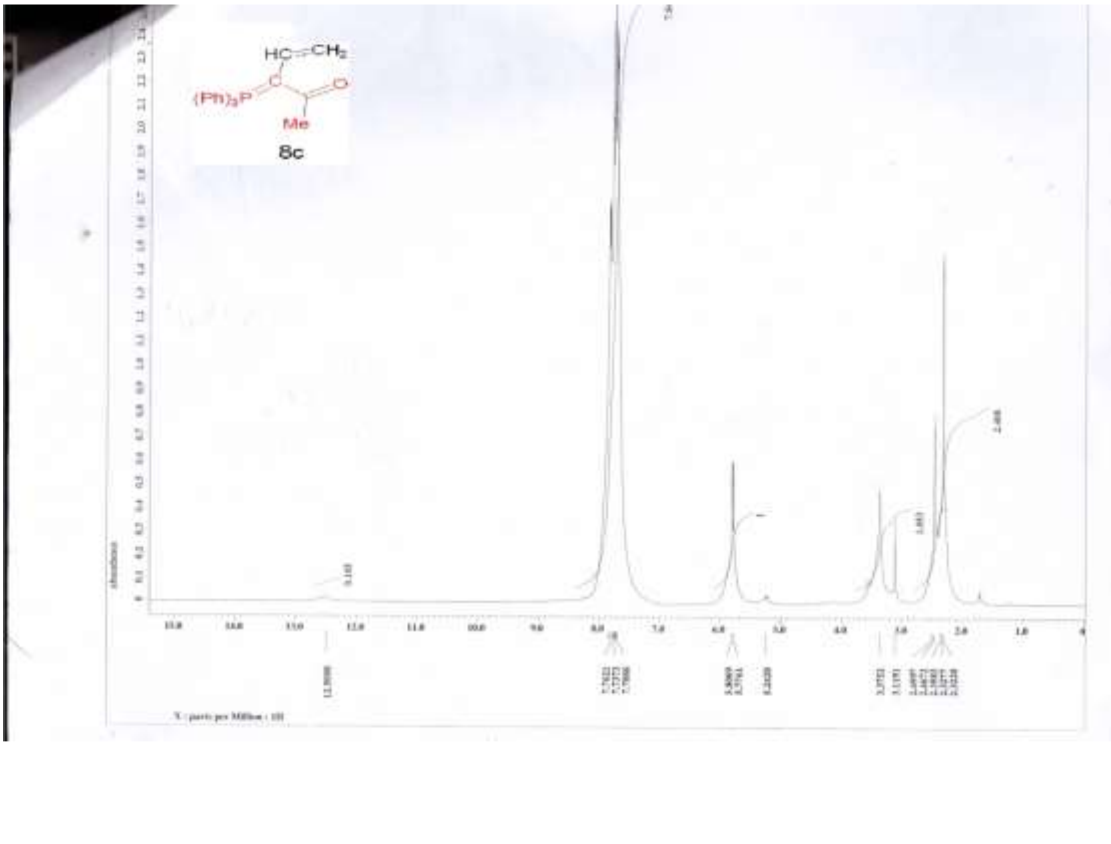
8c
12.9098
7.7622
7.7373
7.7066
3.8069
3.7761
3.2430
3.3752
3.1191
2.469
2.4472
2.3882
2.3277
2.3218
X : parts per Million : 1H

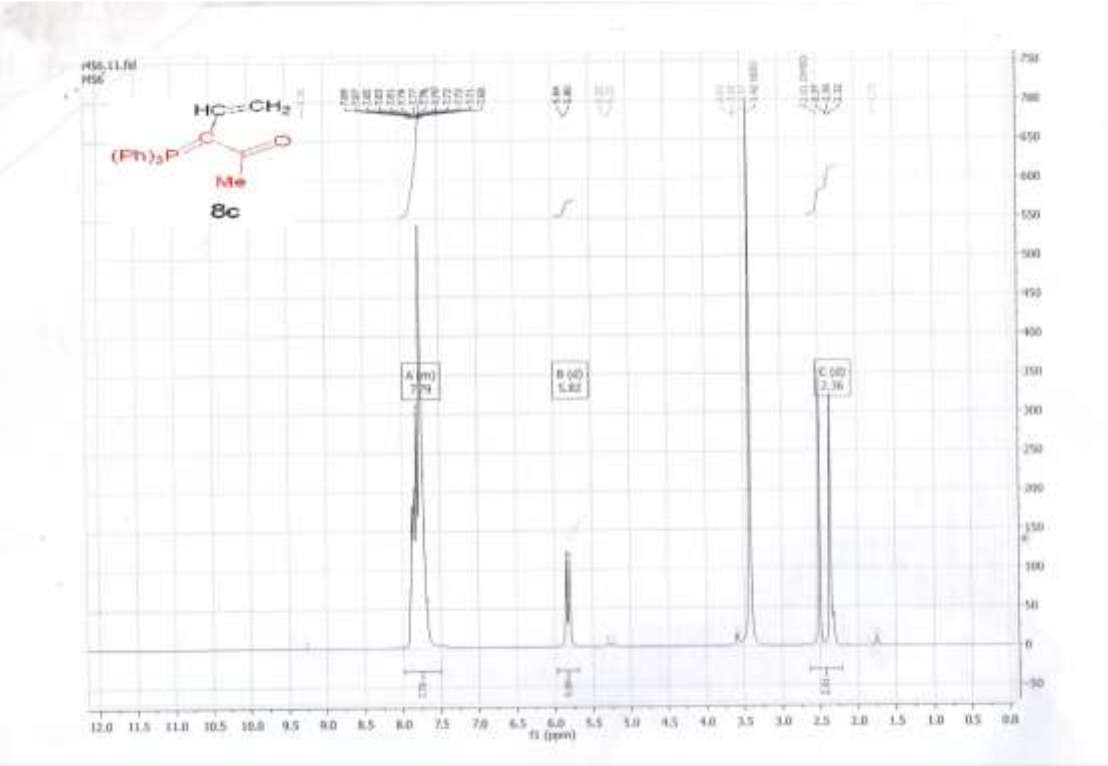

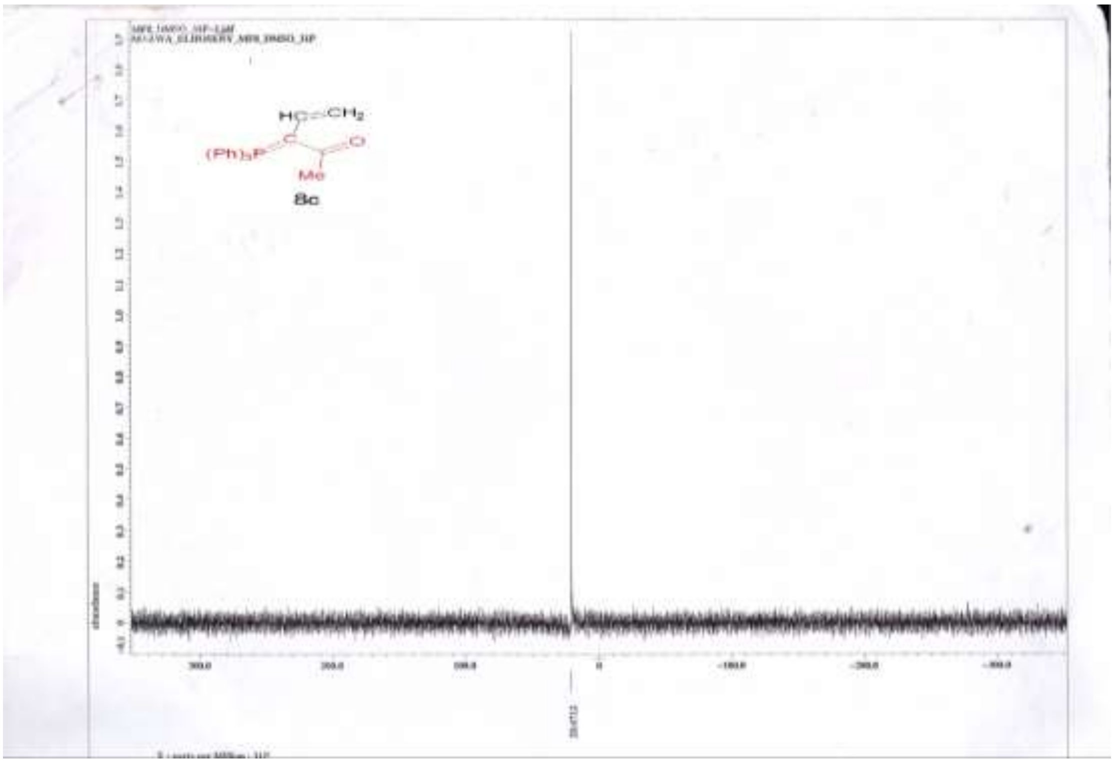

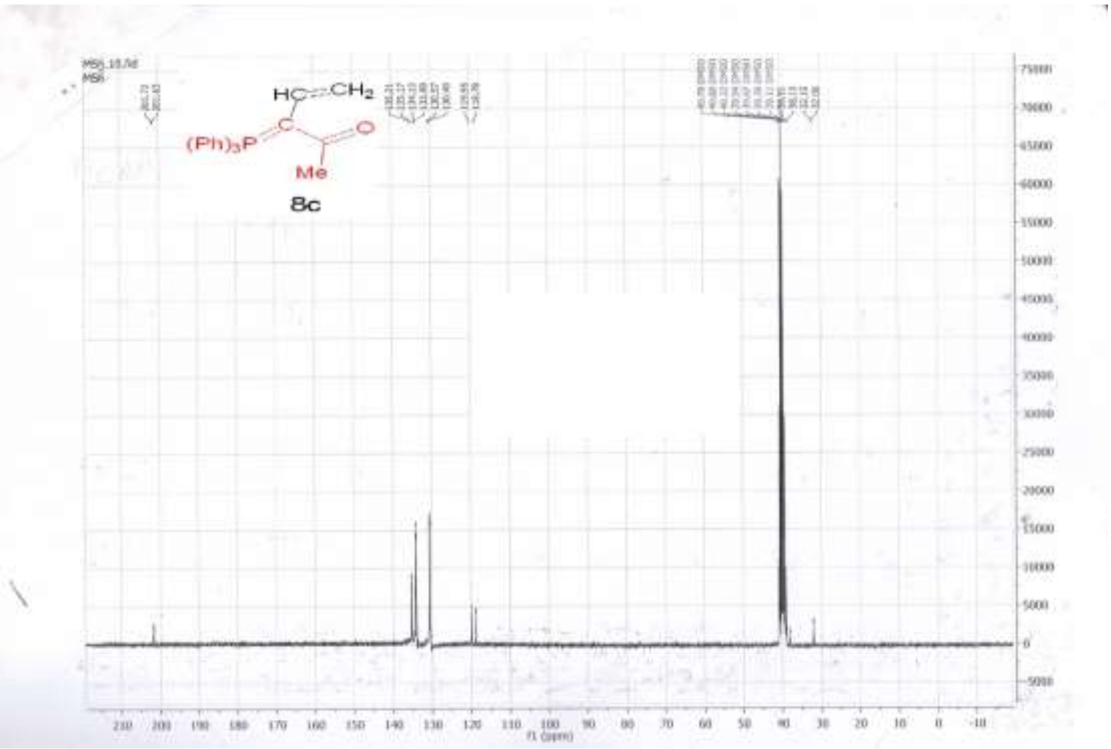

^{1}HNMR, ^{13}CNMR, ^{31}PNMR and IR compound 8d

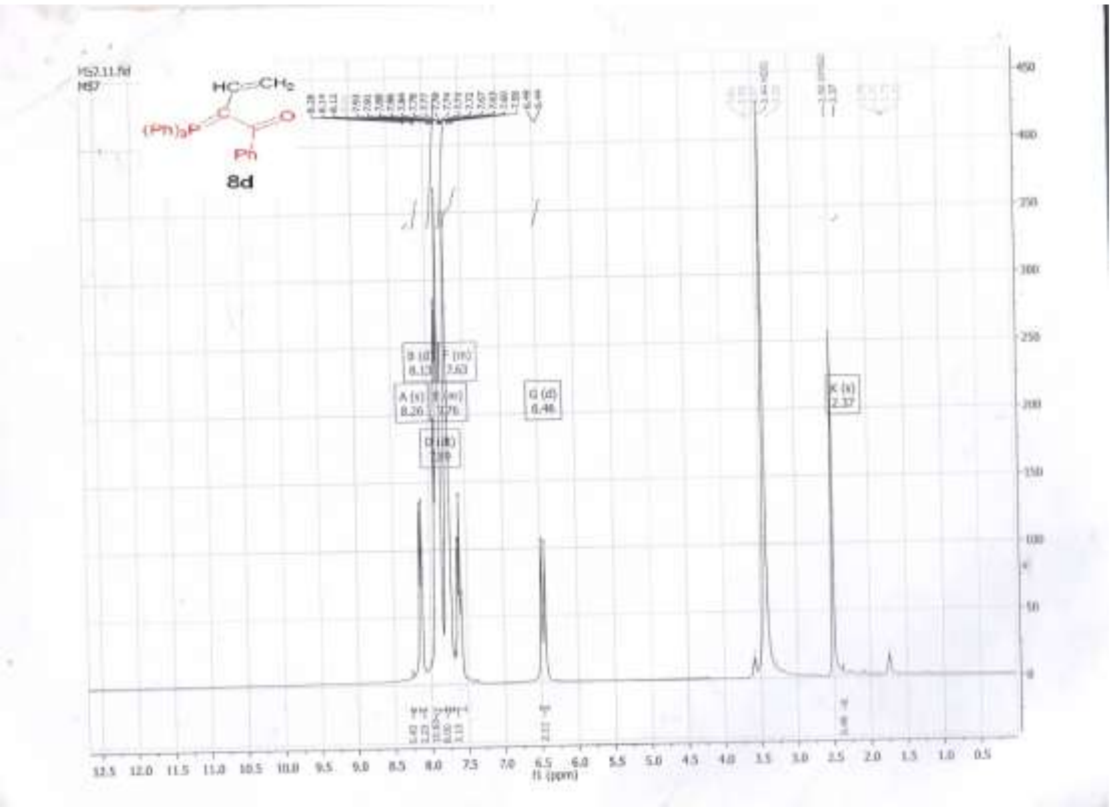

Information Classification: General

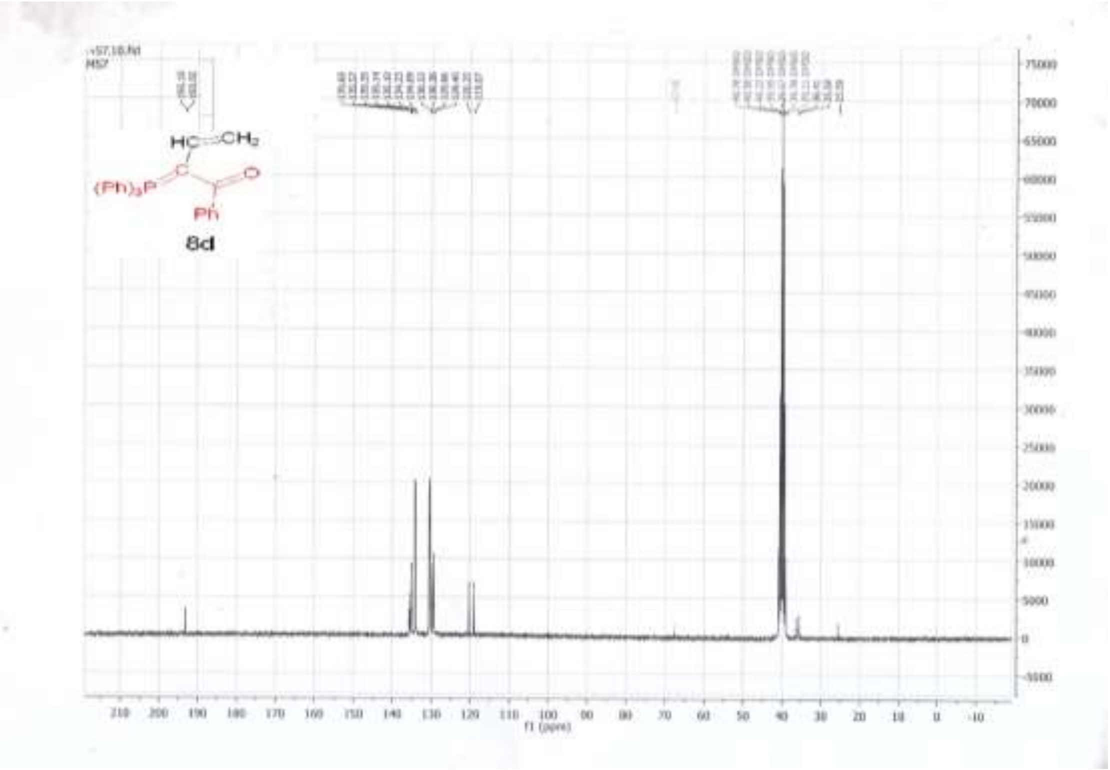
HC=CH2
(Ph)3P=C
Ph
8d
f1 (ppm)

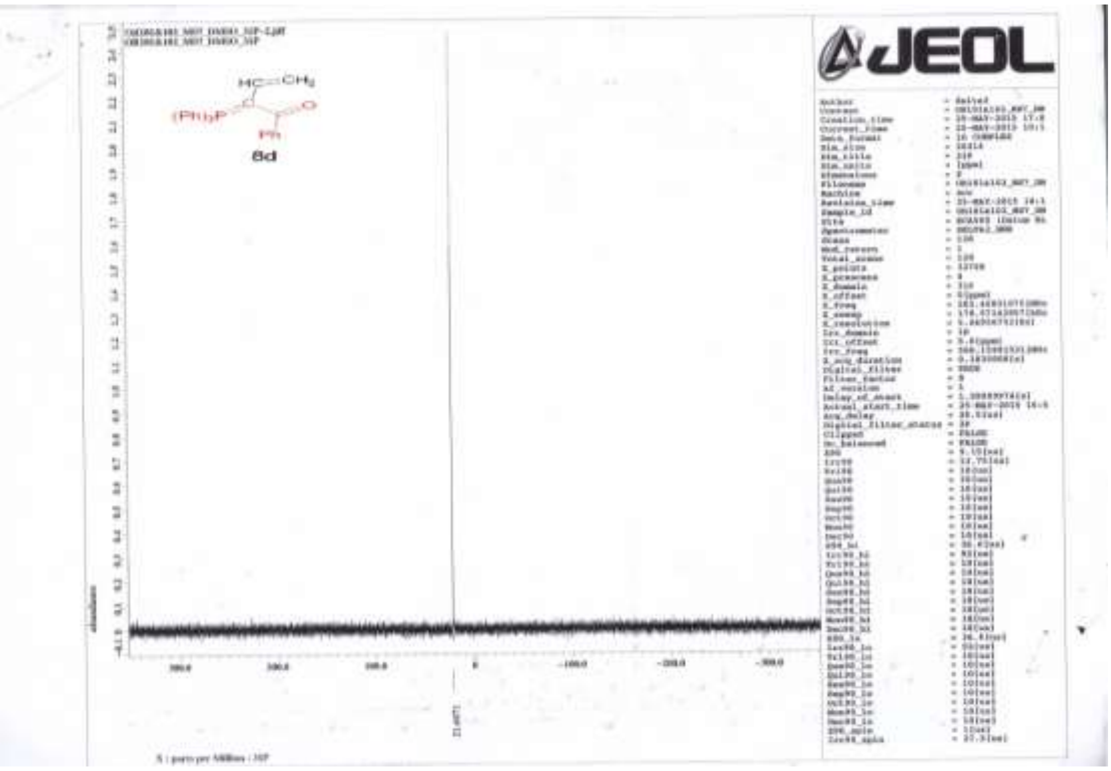
HC=CH2
(Ph)3P
Ph
8d
JEOL

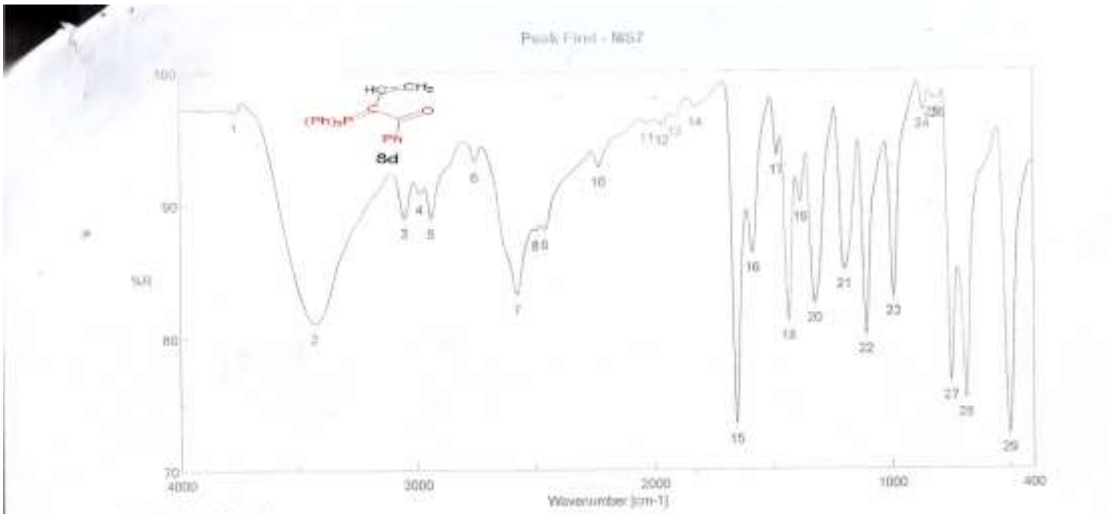

[Result of Peak Picking]

No.	Position	Intensity	No.	Position	Intensity	No.	Position	Intensity	No.	Position	Intensity
1	3770.15	96.9363	2	3431.71	81.0889	3	3050.83	88.9861	4	2986.23	90.7417
5	2936.98	88.9348	6	2763.88	93.1453	7	2578.36	83.2306	8	2499.29	88.0284
9	2459.76	88.0846	10	2233.16	92.7474	11	2024.89	96.0326	12	1963.18	95.8778
13	1910.15	96.5345	14	1819.51	97.2131	15	1604.62	73.4264	16	1585.16	86.3369
17	1481.06	93.6149	18	1434.76	81.2274	19	1382.71	90.0898	20	1323.89	82.5472
21	1198.54	85.0857	22	1100.94	80.1769	23	991.232	83.0412	24	862.989	97.0253
25	822.491	97.89	26	796.457	97.8361	27	748.245	76.631	28	686.57	75.3552
29	501.401	72.6901									

MS, ^31^PNMR, and IR compound 11a

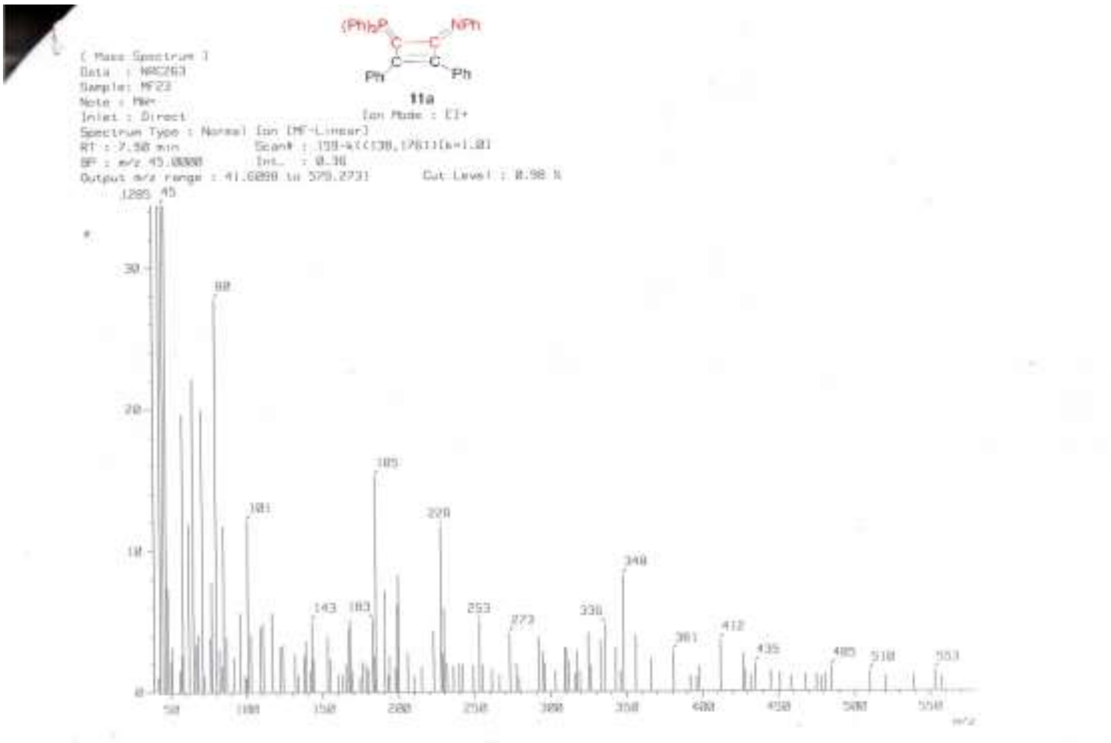

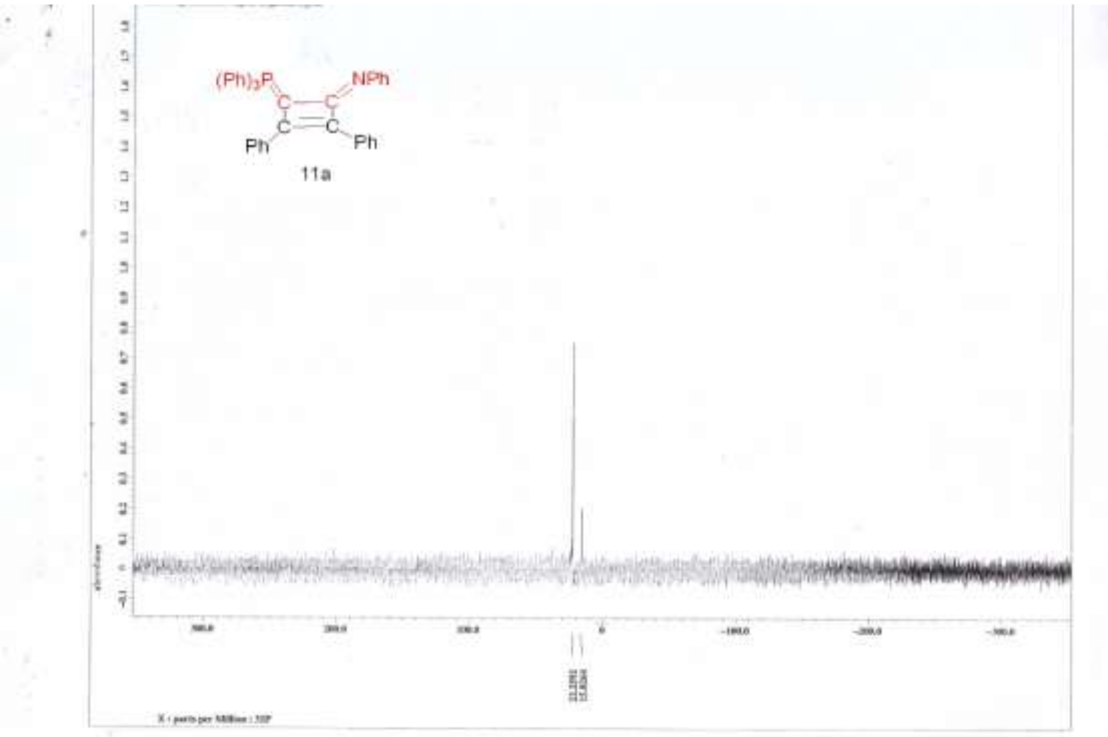

Peak Find - Memory-30

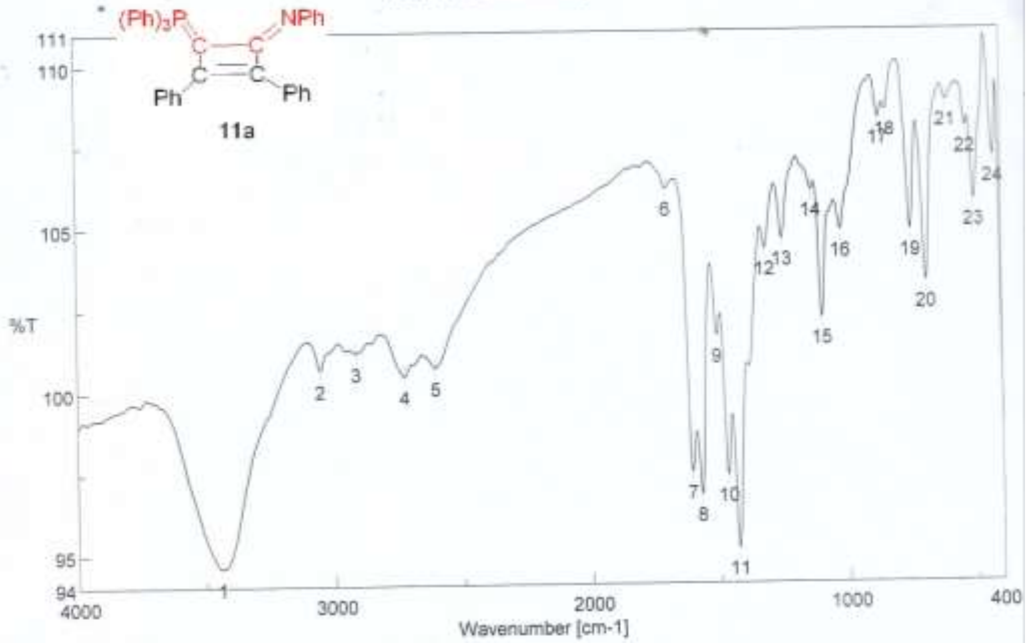

[Comments]

Sample name	MF 23
Comment	10/2019
User	IR
Division	IR
Company	MAC

[Result of Peak Picking]

No.	Position	Intensity	No.	Position	Intensity	No.	Position	Intensity
1	3439.42	94.5902	2	3053.73	100.657	3	2915.84	101.162
4	2726.85	100.45	5	2606.32	100.698	6	1705.73	106.12
7	1609.31	97.4286	8	1573.63	96.7373	9	1511.92	101.615
10	1472.38	97.3367	11	1431.89	95.077	12	1323.89	104.27
13	1257.36	104.565	14	1141.65	106.048	15	1102.12	102.15
16	1027.87	104.812	17	876.488	108.256	18	848.525	108.493
19	754.031	104.816	20	694.248	103.238	21	611.324	108.779
22	536.114	107.958	23	505.258	105.726	24	429.084	107.046

^{31}PNMR, ^{1}HNMR, IR, MS compound 11b

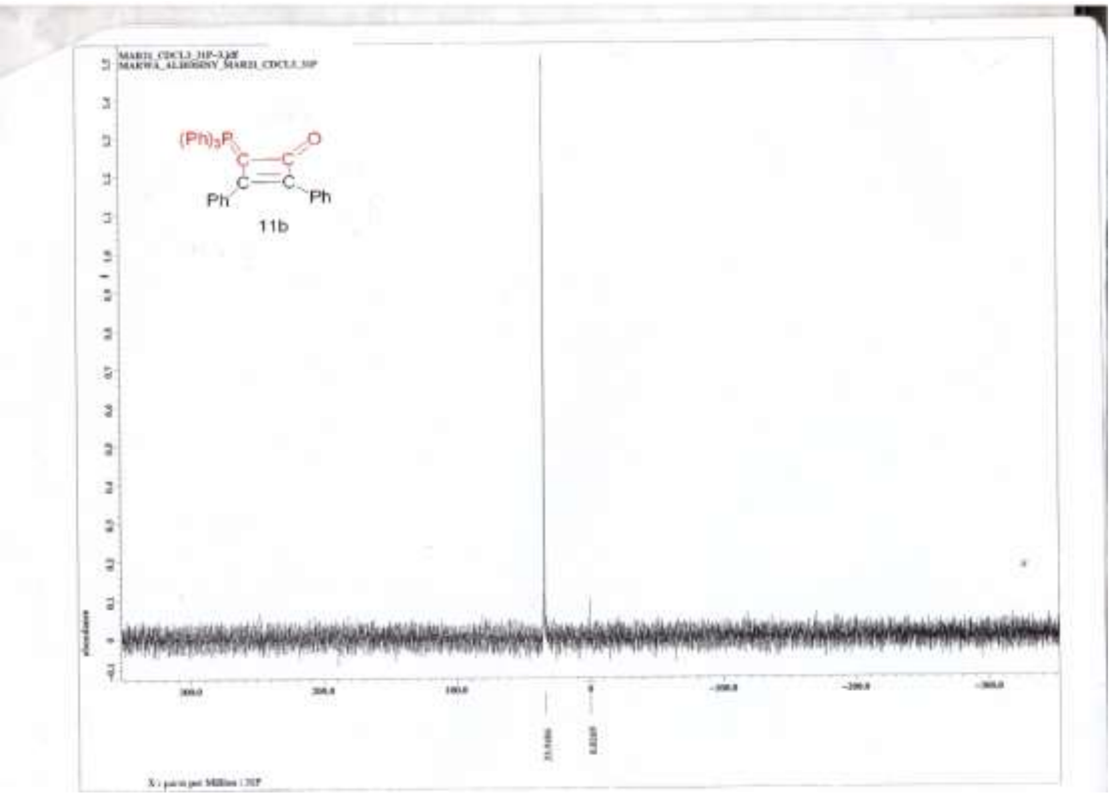

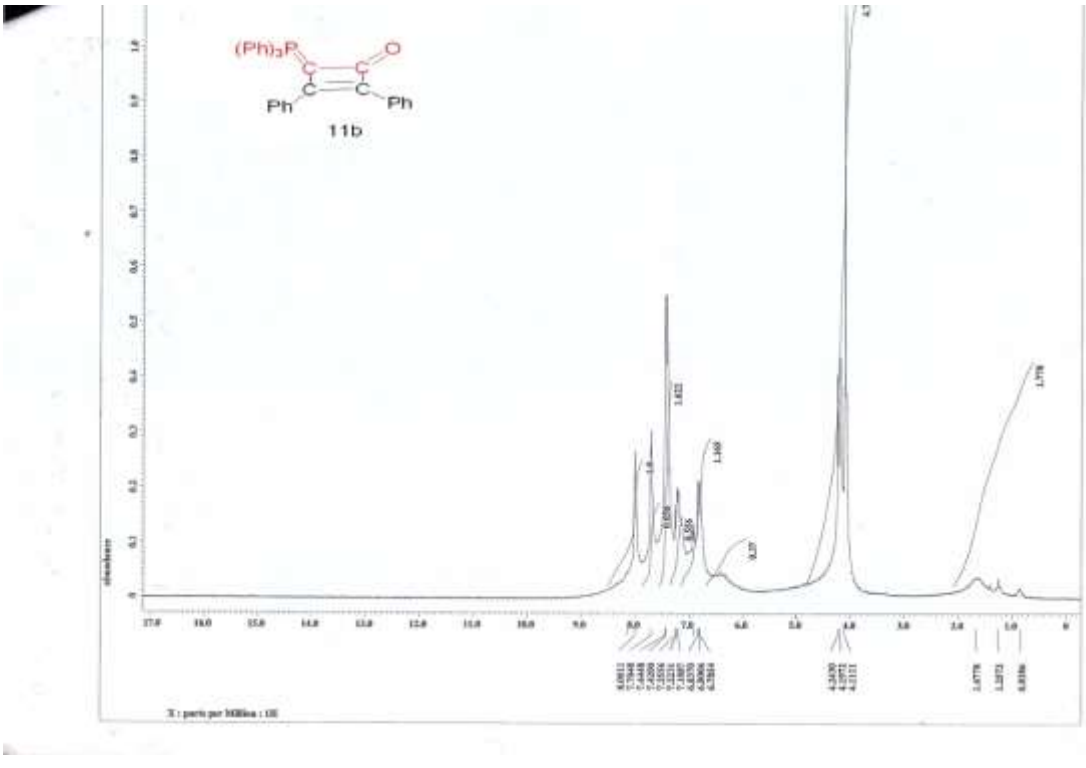

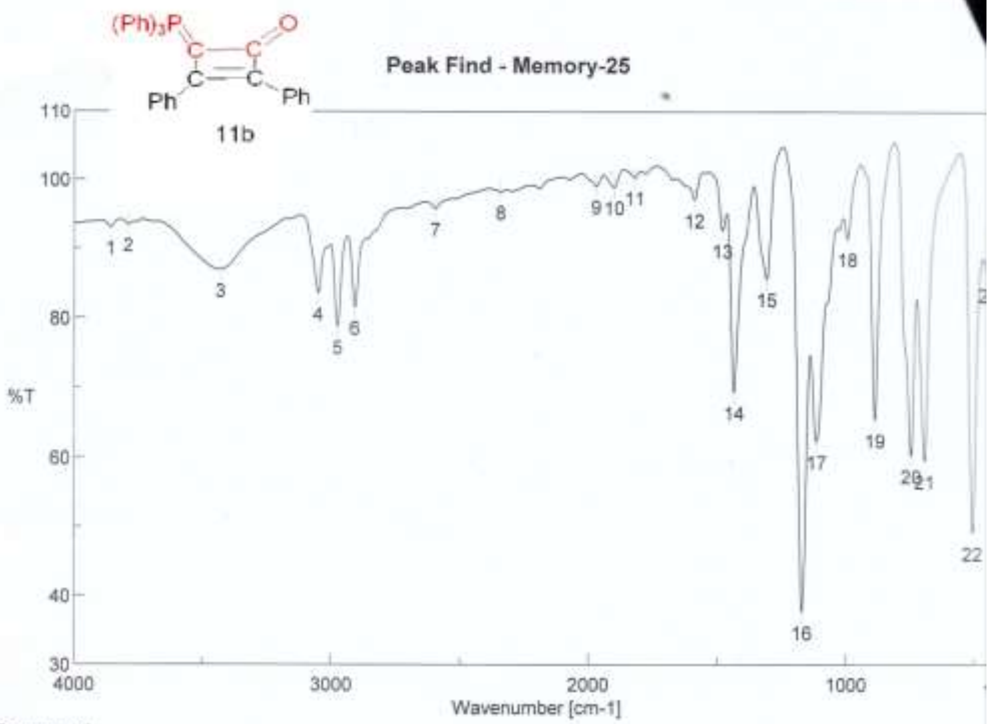

[Comments]

Sample name	MF 21
Comment	10/2019
User	IR
Division	IR
Company	MAC

[Result of Peak Picking]

No.	Position	Intensity	No.	Position	Intensity	No.	Position	Intensity
1	3856.93	93.0963	2	3787.51	93.4636	3	3429.78	86.9325
4	3051.8	83.5958	5	2977.55	78.7948	6	2910.06	81.6099
7	2596.68	95.7991	8	2342.12	98.2094	9	1969.93	99.064
10	1901.47	98.9149	11	1820.47	100.367	12	1590.02	97.0905
13	1482.03	92.706	14	1436.71	69.3914	15	1309.43	85.6952
16	1173.47	37.6221	17	1116.58	62.3573	18	996.053	91.4988
19	888.059	65.4595	20	747.281	60.325	21	694.248	59.5285
22	507.187	49.0883	23	445.476	86.3573			

Micro Analytical Center

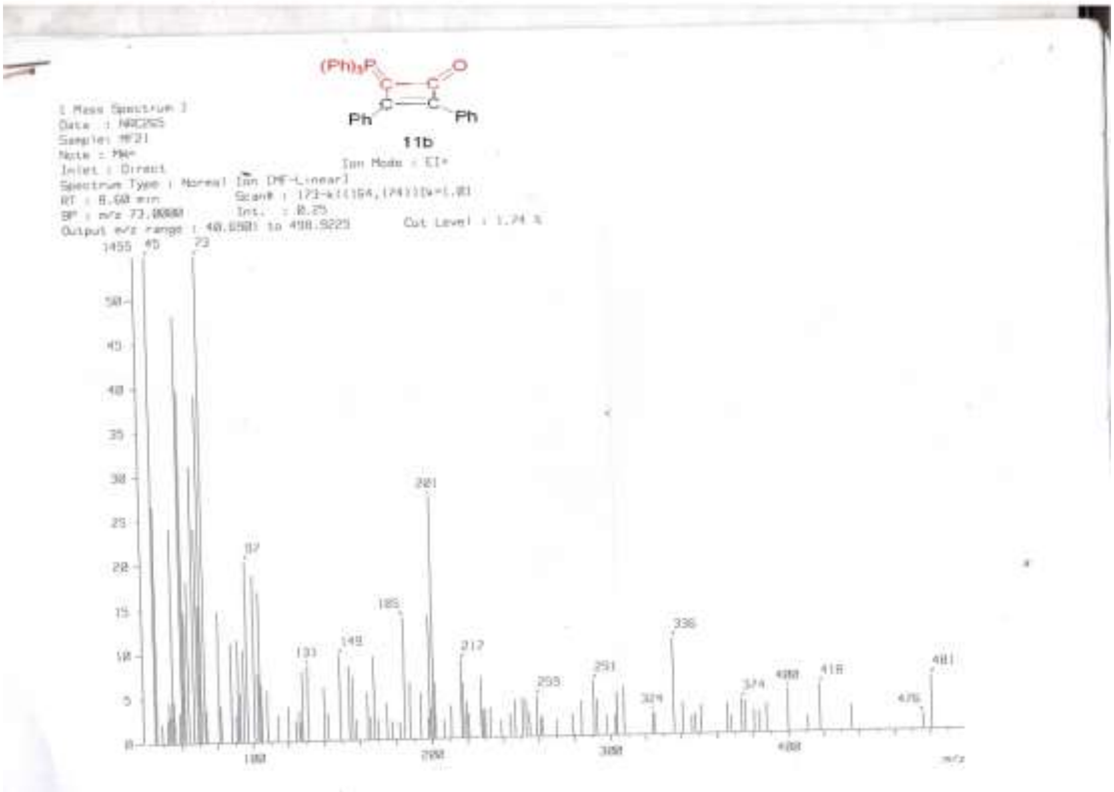

^{1}HNMR, IR, MS compound 13a

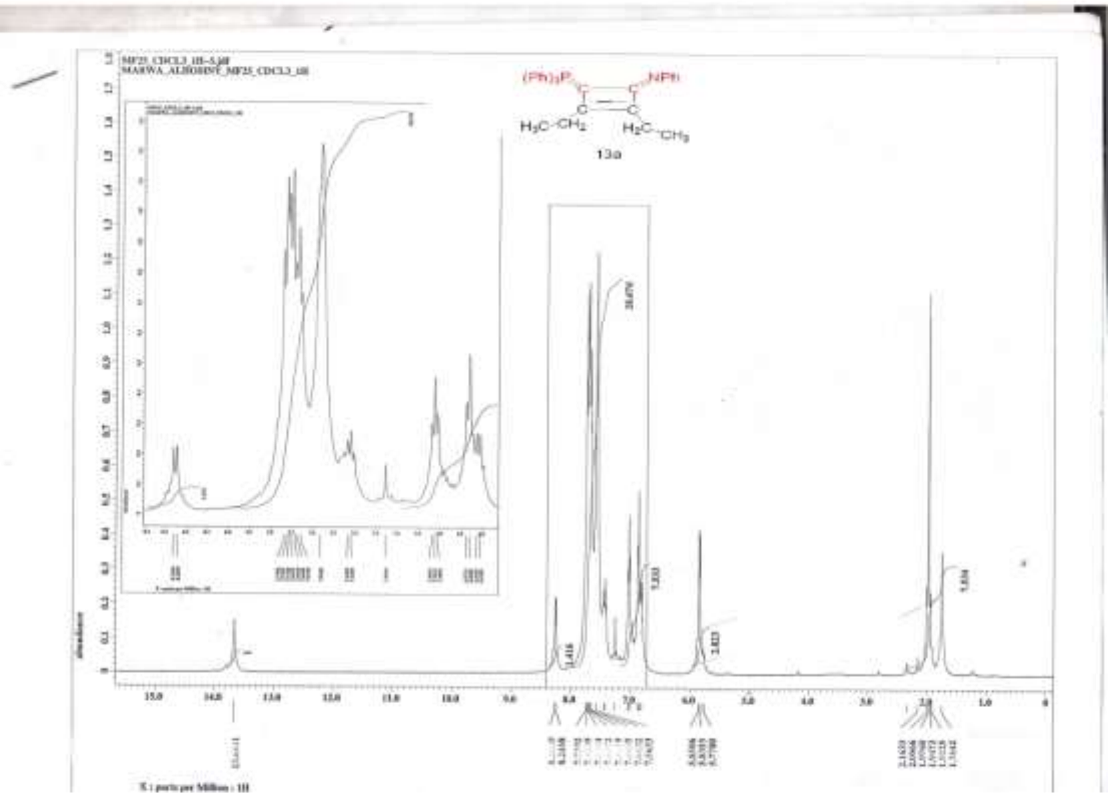

Peak Find - Memory-60

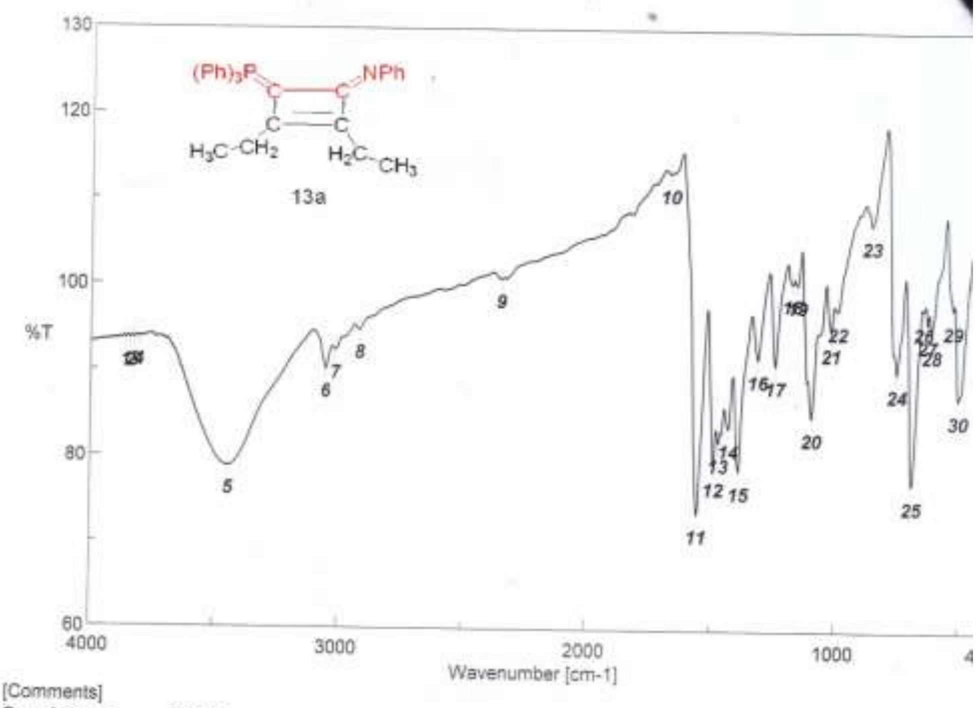

[Comments]
Sample name MF25
Comment 13/11/2013
User IR
Division IR
Company Micro Analytical Center

[Result of Peak Picking]

No.	Position	Intensity	No.	Position	Intensity	No.	Position	Intensity
1	3858.86	93.5882	2	3840.54	93.5541	3	3824.15	93.6599
4	3806.79	93.7308	5	3443.28	78.8591	6	3054.69	90.2689
7	3016.12	92.4751	8	2918.73	94.8319	9	2348.87	100.858
10	1671.02	113.302	11	1555.31	73.659	12	1490.7	79.1841
13	1471.42	81.9433	14	1430.92	83.6306	15	1389.46	78.6746
16	1314.25	91.7219	17	1244.83	91.0199	18	1178.29	100.631
19	1159.97	100.483	20	1098.26	84.9915	21	1020.16	94.8983
22	995.089	97.4497	23	860.096	107.452	24	754.031	90.2023
25	691.355	77.1458	26	647.965	97.5221	27	630.609	95.9965
28	614.217	94.8493	29	528.4	97.6579	30	505.258	87.1903
31	408.835	92.7586						

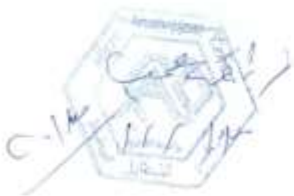

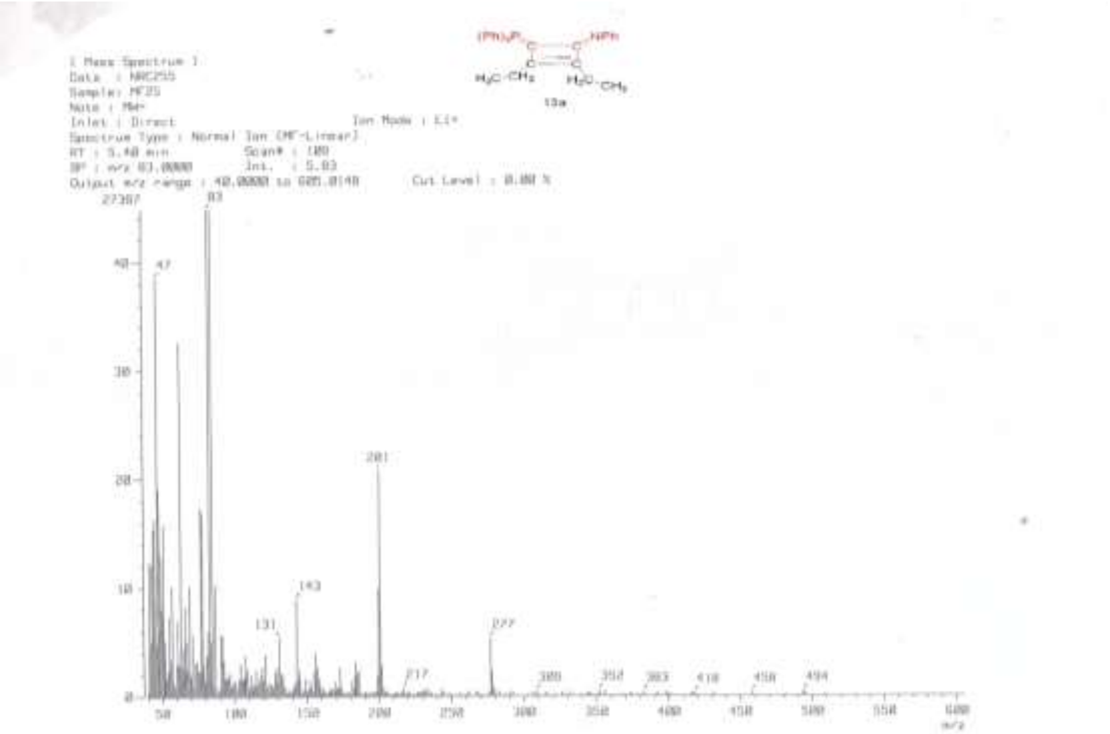

^{31}PNMR, ^{1}HNMR, MS compound 13b

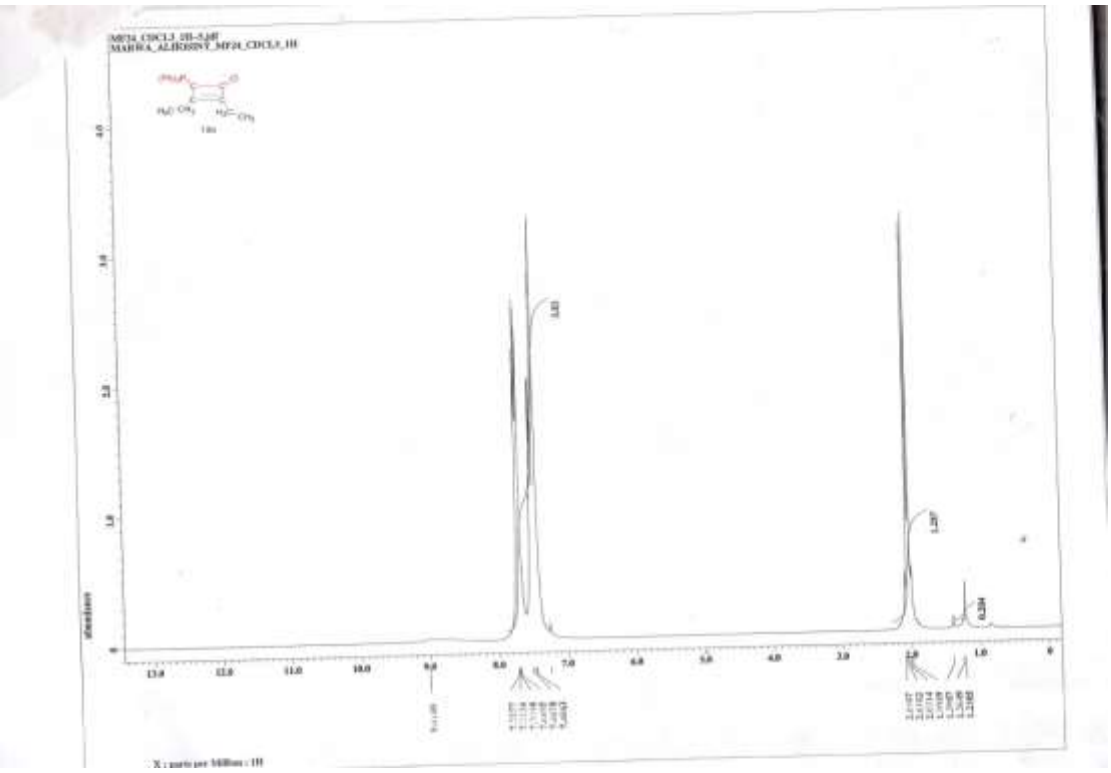

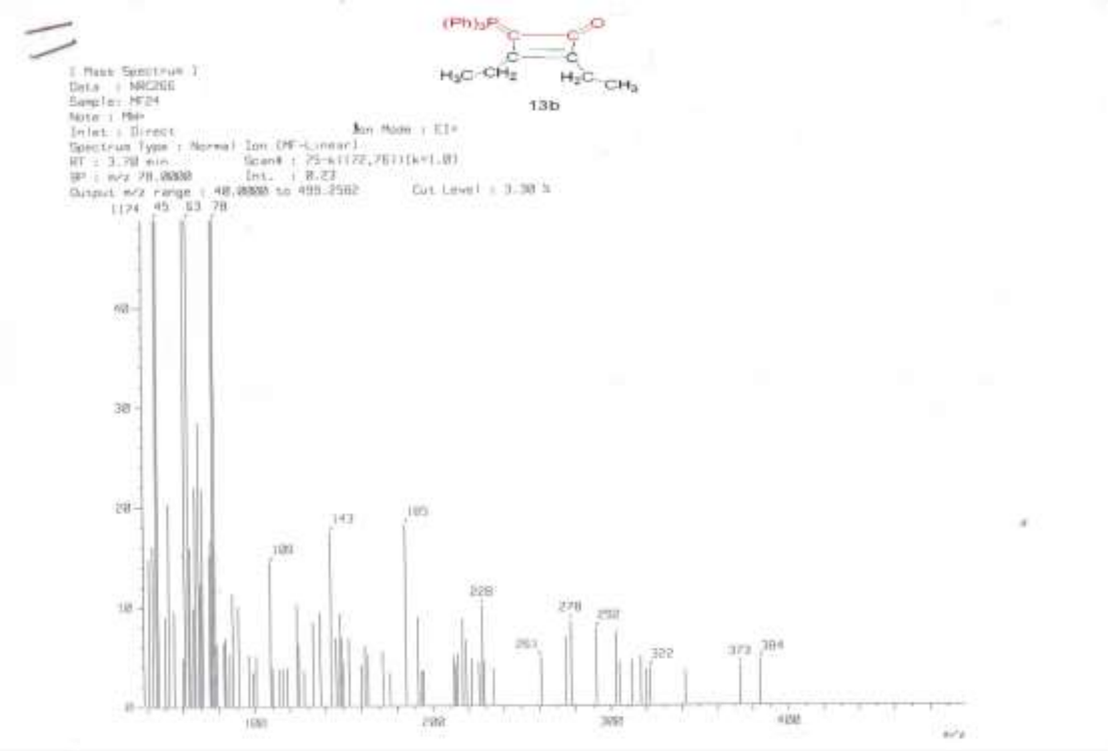

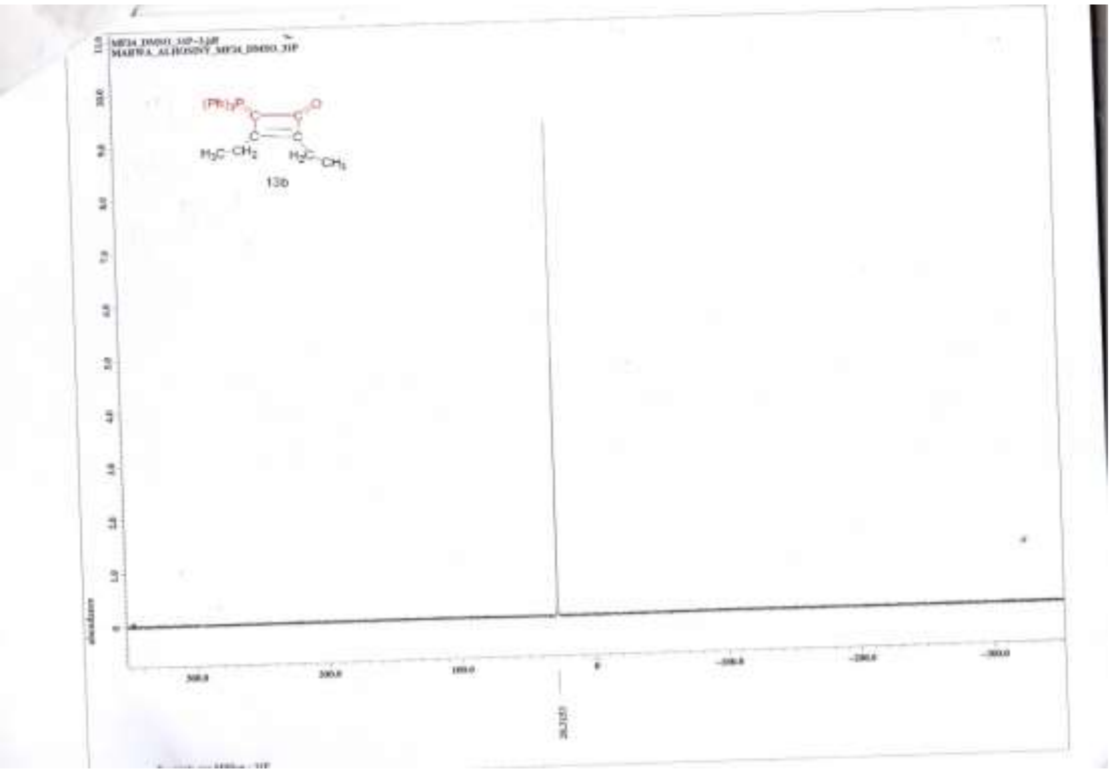

Information Classification: General

^{31}PNMR, ^{1}HNMR, IR compound 15

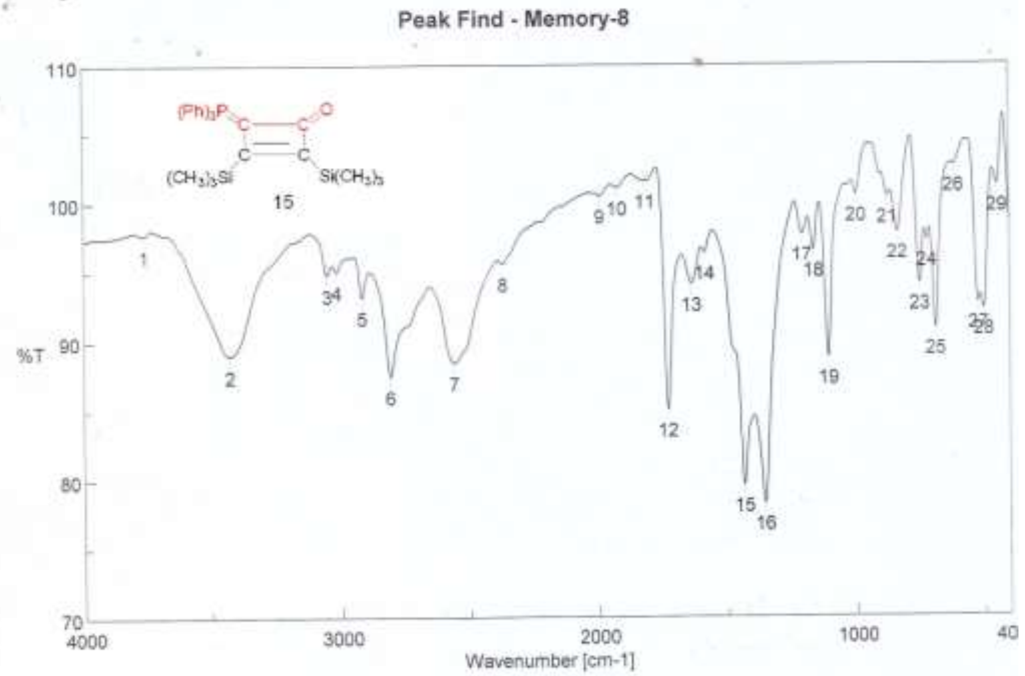

[Comments]
Sample name MS 9
Comment 10/2019
User IR
Division IR
Company MAC

[Result of Peak Picking]

No.	Position	Intensity	No.	Position	Intensity	No.	Position	Intensity
1	3761.47	97.6751	2	3427.85	88.9373	3	3052.76	94.7719
4	3015.16	95.0427	5	2917.77	93.1829	6	2807.85	87.4277
7	2560.04	88.4177	8	2369.12	95.5724	9	1990.18	100.449
10	1922.68	101.031	11	1814.69	101.533	12	1729.83	85.0021
13	1637.27	94.1003	14	1586.16	96.3653	15	1435.74	79.5247
16	1355.71	78.2032	17	1208.18	97.6819	18	1163.83	96.5398
19	1107.9	88.759	20	996.053	100.521	21	873.596	100.38
22	835.026	97.8053	23	749.209	94.1246	24	720.282	97.2415
25	688.463	90.8601	26	615.181	102.627	27	521.65	92.7346
28	500.437	92.2564	29	446.44	101.18			

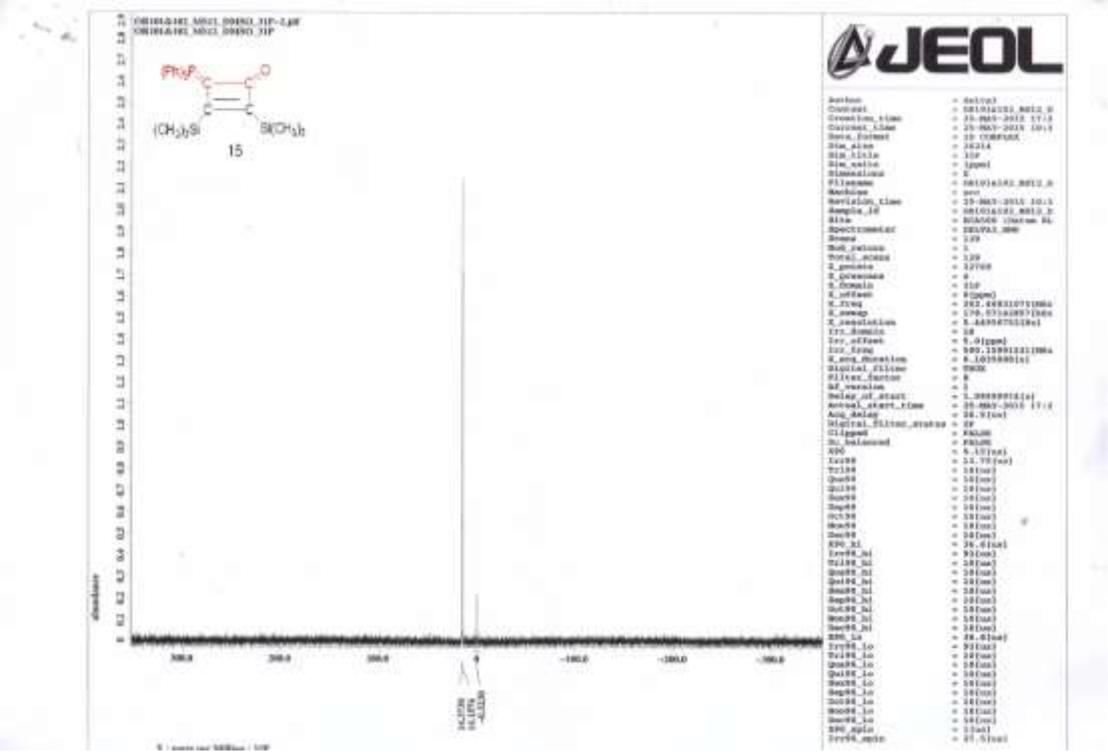

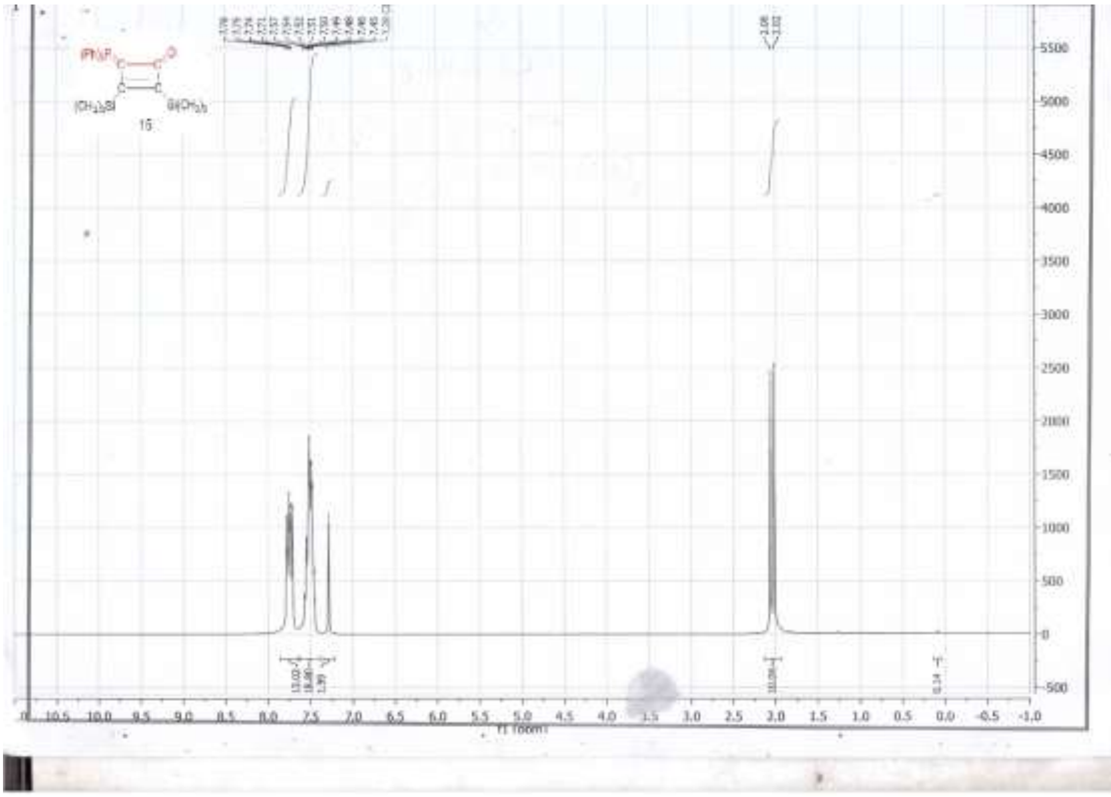

S2. Graphs for IC_{50} calculation of AChE inhibition

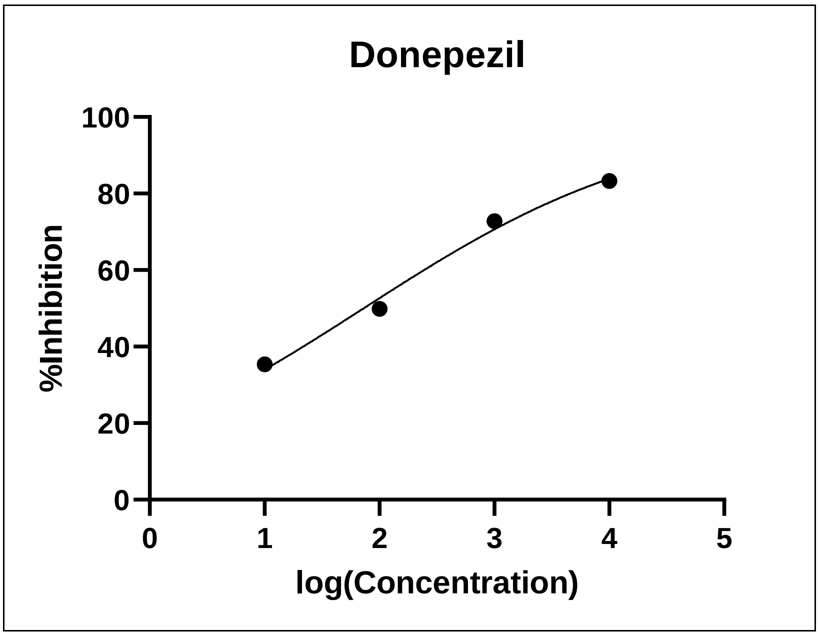

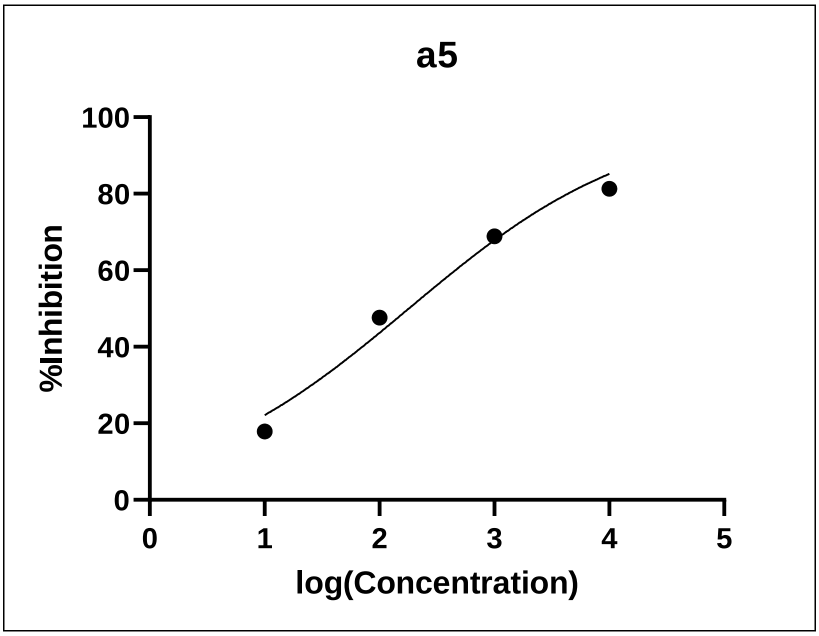

Information Classification: General

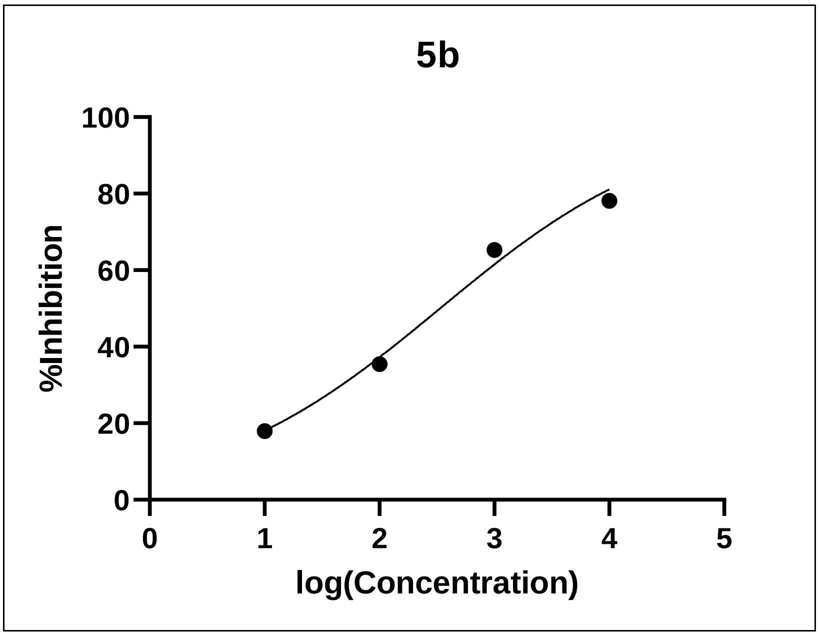

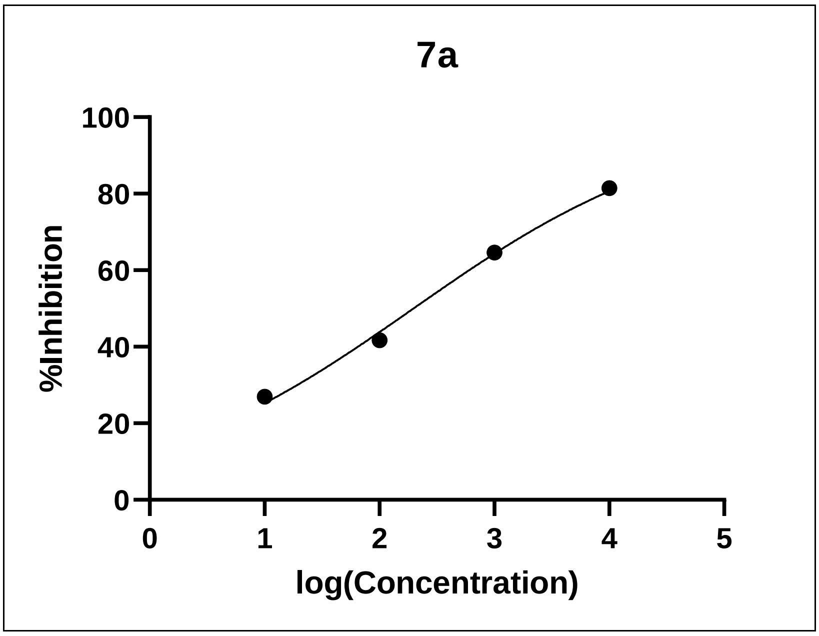

Information Classification: General

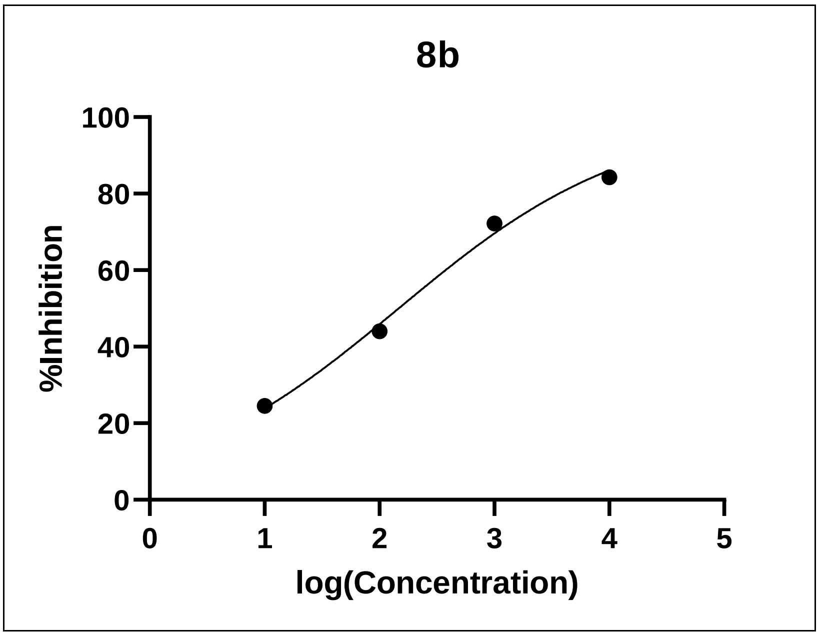

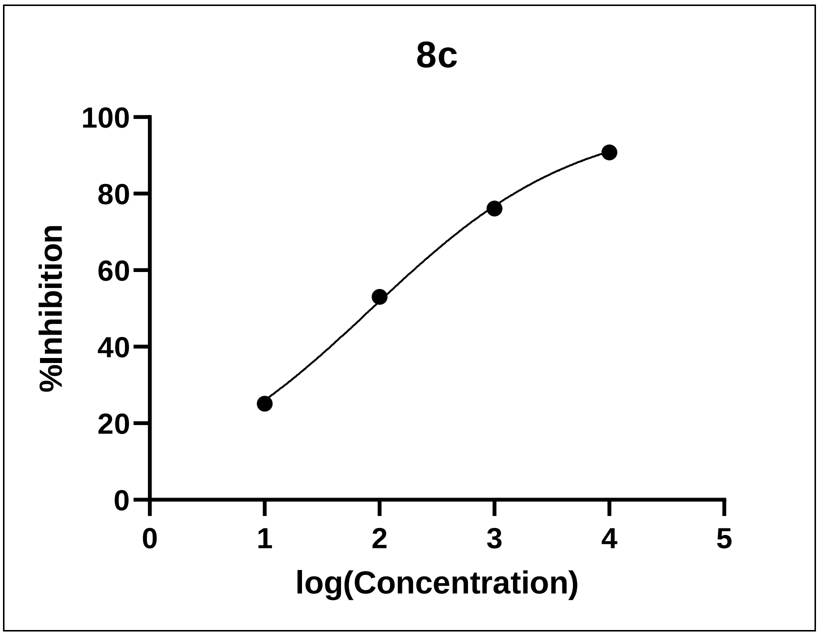

Information Classification: General

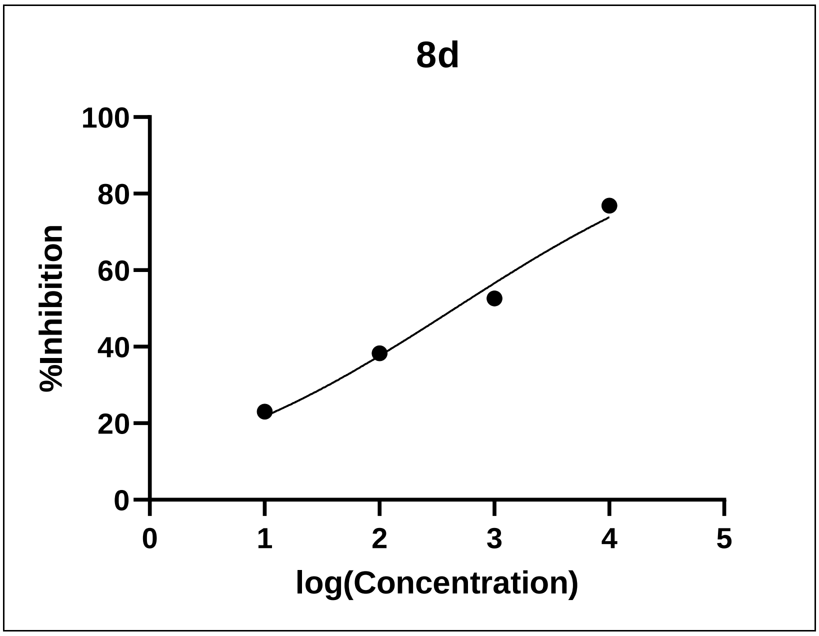

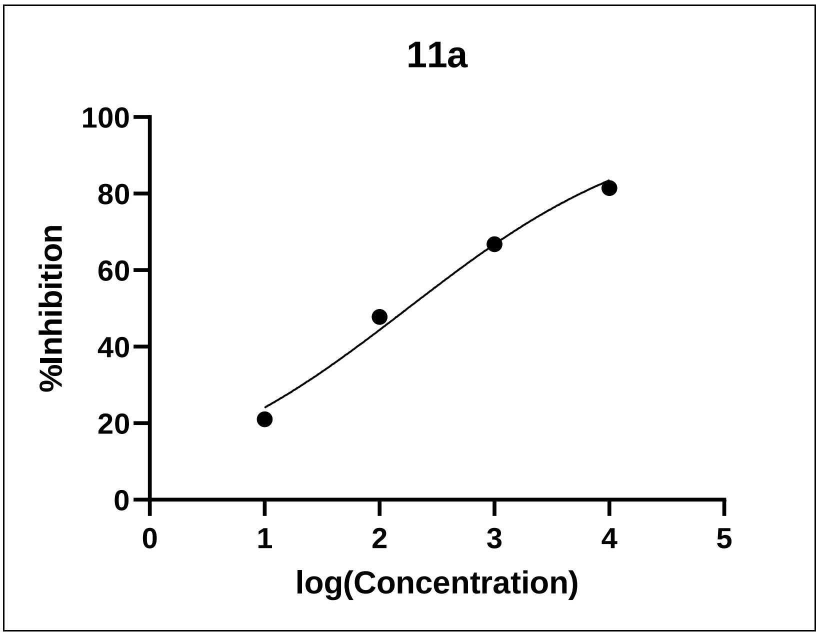

Information Classification: General

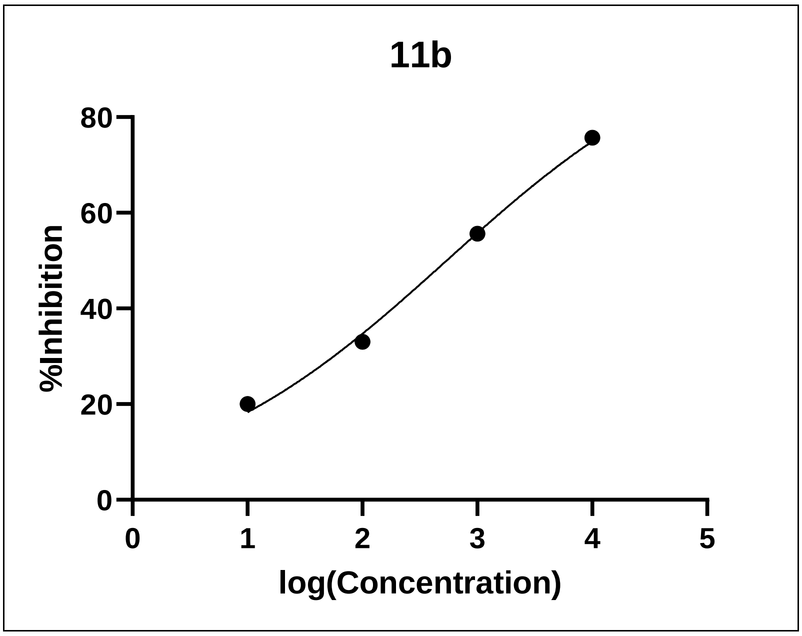

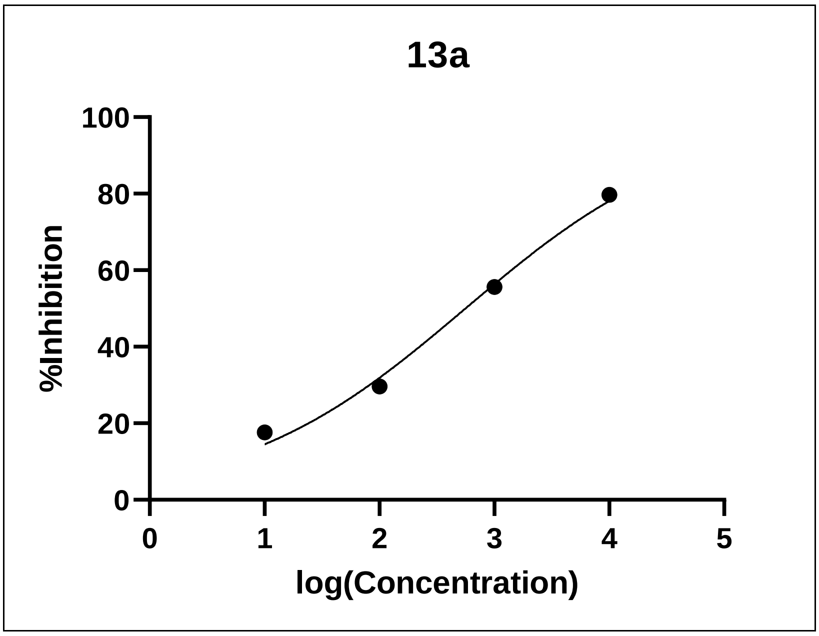

Information Classification: General

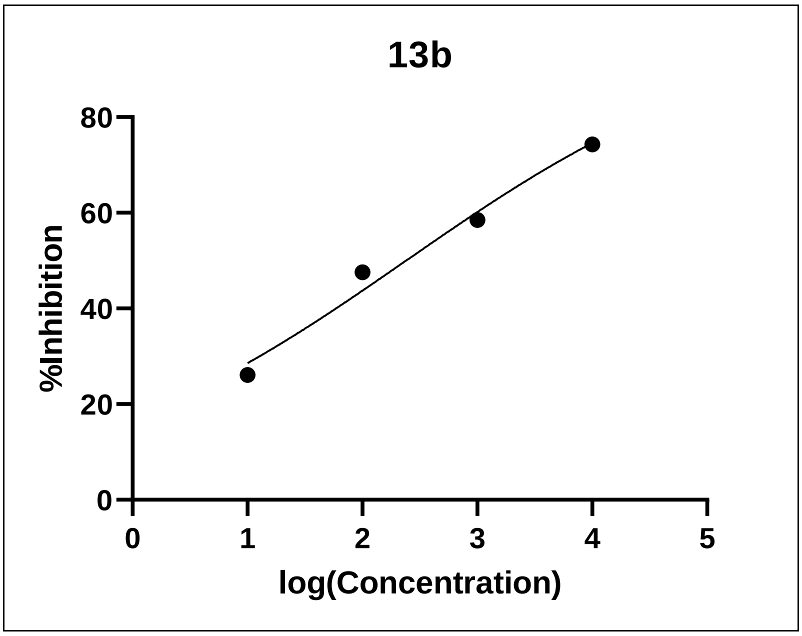
13b
80
60
40
20
0
%Inhibition
0
1
2
3
4
5
log(Concentration)

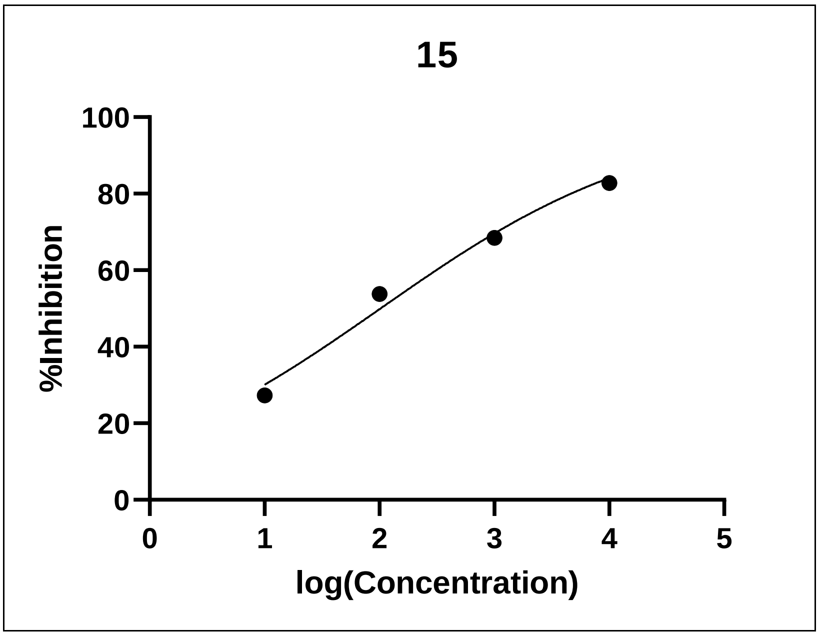
15
100
80
60
40
20
0
%Inhibition
0
1
2
3
4
5
log(Concentration)
